# Phase I/II trial of a long peptide vaccine (LPV7) plus toll-like receptor (TLR) agonists with or without incomplete Freund’s adjuvant (IFA) for resected high-risk melanoma

**DOI:** 10.1136/jitc-2021-003220

**Published:** 2021-08-19

**Authors:** Sapna P Patel, Gina R Petroni, Jason Roszik, Walter C Olson, Nolan A Wages, Kimberly A Chianese-Bullock, Mark Smolkin, Nikole Varhegyi, Elizabeth Gaughan, Kelly T Smith, Kathleen Haden, Emily H Hall, Sacha Gnjatic, Patrick Hwu, Craig L Slingluff

**Affiliations:** 1 The University of Texas MD Anderson Cancer Center, Houston, Texas, USA; 2 University of Virginia School of Medicine, Charlottesville, Virginia, USA; 3 Public Health Sciences, University of Virginia School of Medicine, Charlottesville, Virginia, USA; 4 Icahn School of Medicine at Mount Sinai, New York, New York, USA; 5 Moffitt Cancer Center, Tampa, Florida, USA; 6 Department of Surgery, University of Virginia, Charlottesville, Virginia, USA

**Keywords:** adjuvants, immunologic, melanoma, immunogenicity, vaccine, vaccination

## Abstract

**Background:**

We performed a clinical trial to evaluate safety and immunogenicity of a novel long peptide vaccine administered in combinations of incomplete Freund’s adjuvant (IFA) and agonists for TLR3 (polyICLC) and TLR7/8 (resiquimod). We hypothesized that T cell responses to minimal epitope peptides (MEPs) within the long peptides would be enhanced compared with prior vaccines with MEP themselves and that T cell responses would be enhanced with TLR agonists, compared with IFA alone.

**Methods:**

Participants with resected stage IIB-IV melanoma were vaccinated with seven long melanoma peptides (LPV7) from tyrosinase, gp100, MAGE-A1, MAGE-A10, and NY-ESO-1, each containing a known MEP for CD8^+^ T cells, plus a tetanus helper peptide (Tet) restricted by Class II MHC. Enrollment was guided by an adaptive design to one of seven adjuvant combinations. Vaccines were administered at weeks 1, 2, 3, 6, 9, 12 at rotating injection sites. T cell and IgG antibody (Ab) responses were measured with IFN-gamma ELIspot assay ex vivo and ELISA, respectively.

**Results:**

Fifty eligible participants were assigned to seven study groups, with highest enrollment on arm E (LPV7+Tet+IFA+polyICLC). There was one dose-limiting toxicity (DLT) in Group E (grade 3 injection site reaction, 6% DLT rate). All other treatment-related adverse events were grades 1–2. The CD8^+^ T cell immune response rate (IRR) to MEPs was 18%, less than in prior studies using MEP vaccines in IFA. The CD8^+^ T cell IRR trended higher for IFA-containing adjuvants (24%) than adjuvants containing only TLR agonists (6%). Overall T cell IRR to full-length LPV7 was 30%; CD4^+^ T cell IRR to Tet was 40%, and serum Ab IRR to LPV7 was 84%. These IRRs also trended higher for IFA-containing adjuvants (36% vs 18%, 48% vs 24%, and 97% vs 60%, respectively).

**Conclusions:**

The LPV7 vaccine is safe with each of seven adjuvant strategies and induced T cell responses to CD8 MEPs ex vivo in a subset of patients but did not enhance IRRs compared with prior vaccines using short peptides. Immunogenicity was supported more by IFA than by TLR agonists alone and may be enhanced by polyICLC plus IFA.

**Trial registration number:**

NCT02126579.

## Introduction

Antibodies to CTLA-4 and to PD-1 are approved for adjuvant therapy after surgery for stage IIIB-IV melanoma; however, treatment-associated toxicities can be serious,^1^ and this limits use in earlier stage patients. Thus, there is a need for immune therapies with low toxicity that can target cancer cells with specificity. Melanoma vaccines have promise as an alternative strategy to elicit active antitumor immune responses. Minimal epitope peptides (MEPs) for melanoma-reactive T cells were identified in the 1990s from shared melanoma antigens (eg, tumor associated antigens MART-1/MelanA, gp100, and tyrosinase) and from cancer testis antigens (eg, NY-ESO-1, MAGE-A1, and MAGE-A10).^2^ Clinical activity has been observed with a vaccine targeting gp100, and with adoptive cell therapies targeting MAGE-A3 and NY-ESO-1;^3–6^ however, immune responses to vaccines using short peptides can be transient and of low magnitude, limiting their long-term clinical activity.^7 8^ This may be due to inadequate vaccine adjuvants or to limitations intrinsic to short peptides. Contemporary evidence suggests that vaccination with long peptides (30-mers) may be a more effective strategy to elicit a meaningful immune response.^9^ This approach, combined with incomplete Freund’s adjuvant (IFA), has induced clinical regressions of squamous vulvar neoplasia.^10^ Thus, one goal of the present trial was to assess whether vaccination with long (30-mer) peptides encompassing MEPs for CD8^+^ T cells would be safe and effective for induction of T cell responses to those MEPs. Most prior studies with long peptides in cancer have been limited to those targeting the NY-ESO-1 protein and HPV proteins.^11–13^ The present first-in-human study reports the safety and immunogenicity of multiple long peptides from MAGE proteins and melanocytic antigens.

Vaccines require immunologic adjuvants to induce strong immune responses. A common adjuvant for peptide vaccines has been IFA, in particular Montanide ISA-51, which consists of mineral oil and an emulsifying agent and which has been active in a wide range of human clinical trials of cancer vaccines using short or long peptides.^10 12 14–16^ Murine data have challenged the use of IFA with short peptides,^8^ but our own more recent human experience supports use of IFA to enhance the magnitude and persistence of T cell responses.^17 18^ Toll-like receptor (TLR) agonists can potentiate antitumor T-cell responses.^11 12 18–20^ The TLR3 agonist polyinosinic-polycytidylic acid, stabilized with polylysine and carboxymethylcellulose (polyICLC), preferentially activates BDCA3^+^ myeloid dendritic cells (mDC), supporting the production of IFNβ, CXCL10, and IL12p70.^21^ It has been safely administered with peptides in an emulsion with IFA and has enhanced T cell and antibody (Ab) responses.^12 17^ Resiquimod is an agonist for TLR7 and TLR8 which activates plasmacytoid,^22^ supports differentiation and increased function of mDC,^23^ and reduces proliferation of regulatory T cells.^24 25^ It has been used as a topical gel formulation in murine^26^ and human studies.^24 27^ Preclinical studies support combining agonists for TLR3 and TLR7/8 for synergistic activation of both CD1c^+^ mDC and CD141^+^ mDC^28^ and for promoting differentiation of Th1 CD4^+^ T cell responses and B cell responses.^29^ Thus, there is rationale for using polyICLC and resiquimod alone or together, and with IFA, as adjuvants for long peptide vaccines.

We have assessed the safety and immunogenicity of seven long peptides plus IFA, the TLR3 agonist polyICLC, and/or a TLR7/8 agonist resiquimod in seven different adjuvant combinations, using an adaptive study design.^30^ The long peptides represent portions of melanocytic differentiation antigens and cancer-testis antigens, each 29–31 amino acids long, and each incorporating a defined MEP for CD8 T cells (table 1). A central hypothesis was that vaccination with the long peptide vaccine (LPV7) would induce stronger T cell responses to the MEPs than observed in prior vaccines with MEPs themselves.^15^ It was also anticipated that these long peptides would induce CD4^+^ helper T cell responses. A peptide from tetanus toxoid, known to induce CD4^+^ T cell responses,^15^ was included as well, both to support T cell responses to LPV7 and also to be evaluable for the impact of each adjuvant combination.

**Table 1:** 7 long melanoma peptides used in the vaccines, with the corresponding defined MEPs restricted by MHC Class I

Restricting class I MHC allele	Short peptides	Long peptide (30-mers) in LPV7 for the present proposal	
	Minimal epitope	Sequence (minimal epitope underlined)	Source (# residues)
HLA-A1	Tyrosinase _240-251S_*	FTIPYWDWRDAEKSDICTDEYMGGQHPTN	Tyrosinase _231-259 S_* (29)
HLA-A2	Tyrosinase _369-377_†	SMHNALHIYMDGTMSQVQGSANDPIFLLHH	Tyrosinase _361-390_† (30)
	gp100 _209-217-2M_‡	VPLAHSSSAFTIMDQVPFSVSVSQLRALDG	gp100 _198-227_‡ (30)
	MAGE-A10 _254-262_	VIWEALNMMGLYDGMEHLIYGEPRKLLTQD	MAGE-A10 _245-274_ (30)
HLA-A3	gp100 _17-25_	LLHLAVIGALLAVGATKVPRNQDWLGVSRQL	gp100 _9-39_ (31)
	MAGE-A1 _96-104_	SREEEGPSTSCILESLFRAVITKKVADLVG	MAGE-A1 _82-111_ (30)
HLA-B35/B51	NY-ESO-1 _94-102_	GARGPESRLLEFYLAMPFATPMEAELARRS	NY-ESO-1 _79-108_ (30)

*Substitution of S for C at residue 244.

†Post-translational change of N to D at residue 371.

‡209-2M, substitution of M for T at position 210. The minimal epitope peptides are abbreviated in figures based on the first 3–4 letters.

## Methods

### Participant selection

This was an open-label, phase I/II adaptive design study of LPV7±IFA and TLR agonists in participants with resected Stage IIB-IV melanoma. The protocol is provided as online supplemental material 1. The primary objective was to assess safety of each vaccine combination. A secondary objective was to estimate the immunogenicity of LPV7 in each of the seven adjuvant preparations, with an expectation that CD8^+^ T cell responses to the embedded short peptides would be enhanced compared with prior vaccines with short peptides themselves. A summary goal was to determine the optimal vaccine plus adjuvant combination based on safety and immunogenicity.

10.1136/jitc-2021-003220.supp3 Supplementary data

Patients were eligible if 18 years or older, with ECOG performance status of 0–1 and Stage IIB-IV melanoma (AJCC 7th edition, at original diagnosis or recurrence), rendered clinically free of disease by surgery or other therapy or by spontaneous remission within 6 months of study entry. Patients with brain metastases were eligible if they had had no more than three lesions, none >2 cm in diameter at the time of protocol entry, all completely removed or treated. Eligible patients must have had at least one intact axillary or inguinal node basin. The study was limited to patients expressing HLA-A1, A2, A3, B35, or B51. Ocular primary melanoma patients were not eligible.

### Vaccine and adjuvant selection

Six peptides from gp100, tyrosinase, MAGE-A1, and MAGE-A10 proteins were selected based on high immunogenicity in prior clinical trials using MEPs for CD8^+^ T cells^14 15 31^ restricted by HLA-A1, A2, or A3: long peptides encompassing those MEPs were constructed. Also, the NY- ESO-1 _94-102_ peptide, containing a MEP for CD8^+^ T cells restricted by HLA-B35 and B51, was selected based on immunogenicity in a prior trial and.^12^ The sequences and known immunogenic MEP for each peptide are listed in table 1.

Montanide ISA-51 was purchased from Seppic Inc (Fairfield, New Jersey, USA) as cGMP material in sterile vials. PolyICLC was provided as a clinical grade reagent (Hiltonol; Oncovir, Washington, DC, USA) by the Ludwig Institute for Cancer Research and its Cancer Vaccine Consortium. Resiquimod was provided by 3M Pharmaceuticals (St Paul, Minnesota, USA).

### Study regimen

**Figure 1:**
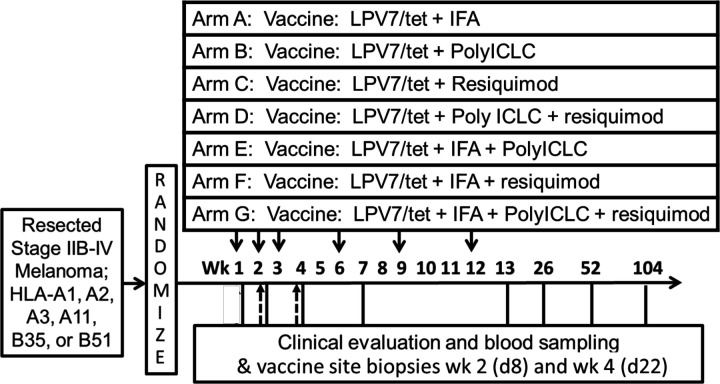
Mel60 protocol schema. Participants with resected high-risk melanoma were randomized among seven study arms in an adaptive design. Each was vaccinated at weeks 1, 2, 3, 6, 9, and 12 in an extremity (50% id/50% sc) with LPV7 plus Tet (solid arrows). PolyICLC (1 mg) was incorporated in the peptide/adjuvant emulsion or mixtures and injected at the vaccine site. Resiquimod cream was administered topically at the vaccine site, 112.5 mcg. Blood was drawn at weeks 1, 2, 4, 7, 13, 26, 52, and 104. Vaccine sites were biopsied on days 8 and 22 (dotted line arrows).

Participants were vaccinated with LPV7 (300 mcg long peptide/dose) plus Tet (200 mcg/dose) on the schedule shown in figure 1. Tet was included to provide a stimulus for helper T cell responses to support CD8^+^ T cell responses,^14 15 32^ because the extent of CD4^+^ T cell response to LPV7 was not known in advance. Participants were adaptively assigned to one of seven adjuvant combinations (figure 1 and online supplemental table 1) and were enrolled at two institutions. The adaptive assignment included equal randomization and allocation among allowable arms until a weighted allocation scheme or the modeling stage was triggered. Vaccine sites were rotated between upper arm and thigh, avoiding any extremity that had undergone lymph node removal. Each vaccine was administered in one skin site, with 1 mL volume injected into subcutaneous (sc) tissue and 1 mL volume injected intradermally (id), through the same skin puncture site. Each participant underwent three 4 mm punch biopsies of skin at the vaccine injection site at days 8 and 22. Control biopsies of normal skin were also collected at these time points for the first six participants enrolled.

10.1136/jitc-2021-003220.supp2 Supplementary data

### Safety and toxicity

Adverse events (AEs) were collected continuously from the time of first injection to study completion and the AEs were graded using NCI CTCAE V.4.03. Dose-limiting toxicities (DLTs) were defined as any unexpected, treatmenft-related AE that was ≥Grade 1 ocular AE, ≥Grade 2 allergic/autoimmune reaction, or ≥Grade 3 (any) except for Grade 3 injection site reaction (ISR) with ulceration ≤2 cm.

### Immunologic response

Peak CD4^+^ and CD8^+^ T cell responses in the peripheral blood were evaluated by direct (ex vivo) ELIspot assay for IFN-gamma, as reported.^14^ This was performed in a core laboratory dedicated to immune monitoring, with standard operating procedures and quality assurance measures.^14^ Participants were evaluable for immune response if at least one post-treatment sample was measurable for response. A minimum of 4 weeks of data was used to guide decisions about the range of optimal dose combinations. Durability of T cell responses was assessed at 26 weeks and for longer intervals when available, with the hypothesis that durability of T cell responses would be enhanced compared with prior experience with short peptides and would be improved with TLR agonists when compared with IFA alone in this study.

The short peptides (9–12 amino acids long) contained within each long peptide (table 1, underlined) represent MEPs for CD8^+^ T cells. A CD8^+^ T cell response to these peptides was defined as at least a twofold increase in IFN-gamma-secreting cells over background (maximum of 2 negative controls) and over pre-existing responses at baseline, an increase over background of at least 20 IFN-gamma secreting cells per 100,000 CD8^+^ T cells, and no overlap in SD with the negative control, as described.^14^ A CD4^+^ T cell response to the Tet was defined similarly but with at least 20 IFN-gamma secreting cells per 100,000 CD4^+^ T cells. A T cell response to the full-length LPV7 required an increase over background of at least 10 IFN-gamma secreting cells per 100,000 total peripheral blood mononuclear cells (PBMC). Immune response rate (IRR) is defined as the proportion of subjects with a T-cell immune response and is reported as a point estimate with 90% CIs.

Interassay coefficients of variation (CVs) were calculated for the ELIspot response of two normal donors to a pool of viral peptides (CEF peptide pool^33^): for the high and low responders, mean numbers of spots per 200,000 cells were 271 and 42, respectively, and CVs were 28% and 26%, respectively.

### Serum antibody responses to LPV7 long peptides

Sera were evaluated by ELISA for IgG Ab to the LPV7 peptide pool at weeks 1, 7, 13, and 26, using methods described.^12^ Briefly, 96-well half-area cluster plates (Corning Costar) were coated with 30 mcL of LPV7 peptides (pooled) diluted in carbonate/bicarbonate buffer (pH 9.4; Sigma-Aldrich) at 1.67 mcg/mL of each peptide. For quantitation of specific serum levels of anti-peptide Ab, purified IgG immunoglobulin (Fitzgerald Industries International) was prepared in coating buffer at 1 mcg/mL, serially diluted fourfold to 0.25 ng/mL, and 30 mcL of each dilution added to duplicate wells. After incubation overnight at 4°C, plates were washed with phosphate-buffered saline (PBS) with 0.1% Tween 20 (TPBS), then blocked 1 hour with 5% nonfat dry milk in TPBS (blocking 143 buffer). Beginning at 1:100, fourfold serial dilutions of participant and control sera were prepared in blocking buffer and added to individual wells. After 2 hours at room temperature (RT) and washing, secondary Ab (goat anti-human IgG AP conjugate, Southern Biotech) was added to all wells, incubated 1 hour at RT, then washed. Attophos substrate (Sigma) was added to each well for 30 min. 3N NaOH was added to stop the reaction, and fluorescence was recorded on a Molecular Devices SPECTRAmax Gemini EM Fluorescent plate reader, excitation 450 nm, emission 580 nm. The FORECAST function in Microsoft Excel was used to calculate the Ab titer of participants' sera,^12^ which was defined as the reciprocal of the serum dilution that yields a fluorescent intensity 10 times greater than the cut-off value. The cut-off value was defined as the average fluorescence obtained from the first four dilutions of serially diluted normal donor serum (negative control). Ab titers≥100 were considered positive. For participants with Ab titers that were >100 in prevaccine sera, a vaccine-associated response also required a titer increase of at least 10-fold (1 log).

### Statistical considerations

The study was designed with two stages. The initial stage accrued participants in cohorts of one per arm (randomized within a zone) until a participant experienced a DLT. The escalation plan for the first stage was based on grouping treatment combinations into three zones (online supplemental table 1), beginning with Zone 1. With this dose-escalation design, participants could be accrued and assigned to other open combinations within a zone, but escalation would not occur outside the zone until the minimum follow-up period of 3 weeks was observed for the first participant accrued to a combination. A continual reassessment method (CRM)^34 35^ directed enrollment in the second stage. This method used a selected set of possible orderings of combinations for the DLT probabilities and a working model for the DLT probabilities under each ordering. The CRM model was used to fit the working model with the accumulated data. In the event of a tie between the likelihood values of two or more orderings, then the selected order of combinations was chosen at random from among the tied orderings. The DLT probabilities defined a set of acceptable combinations with a toxicity tolerance of 33%. Assuming at least one optimal combination existed, up to 52 evaluable participants could have been accrued to determine the optimal combination. Simulation results were run to display the performance of the design characteristics, which have been reported.^36^ Immune responses were assessed overall, but differences between enrolling institutions were also explored in the context of data on PBMC function.

The study was not designed to make statistical comparisons between arms. Frequency and magnitude of treatment-related adverse events (TRAEs) were summarized by arm. IRR for defined categories was estimated as point estimates with 90% exact CIs. Graphical representations were used to present study outcomes. Fisher’s exact test was used to assess associations of maximum immune response to maximum TRAE grade and other select AEs. Disease-free survival was defined from start of treatment to recurrence/progression or death from any cause, whichever occurred first. Participants who did not experience an event were censored at date of last contact. Overall survival was defined as the time from start of treatment to time of death from any cause. Disease-free survival and overall survival distributions were estimated by the product-limit method of Kaplan and Meier.

## Results

### Participant characteristics

Total enrollment was 51 participants; however, one did not receive study treatment. Thus, demographic, safety, and immunologic data are reported for 50 participants who were enrolled and treated. These included 30 males (60%) and 20 females (40%), with 5, 7, 4, 6, 16, 6, and 6 treated on arms A-G, respectively (online supplemental file 1, CONSORT diagram). Most patients had Eastern Cooperative Oncology Group (ECOG) performance status (PS) of 0 (90%) and stage III disease at registration (82%). Details are provided in online supplemental table 2. Four patients experienced new metastatic disease on study (arms D (1), E (1), G (2)), but two of them had received all six vaccines (arms E and G): the others received three and four vaccines, respectively; so, all 50 were evaluable for toxicity, immune responses, and clinical outcomes (online supplemental file 1, CONSORT diagram).

10.1136/jitc-2021-003220.supp1 Supplementary data

### Toxicities and adverse events

There was one DLT across all groups: Group E (Grade 3 ISR, 6% in that group), after receiving all six vaccines. All other AEs were Grades 1–2; most common were ISR (90%), fatigue (62%), skin induration (56%), chills (48%), fever, myalgia, and headache (30%), and were similar across groups, but lower for Group C (online supplemental table 3). No study combinations crossed the statistical boundaries for unacceptable toxicity.

### Immune responses to vaccines

#### T cell response to LPV7

**Figure 2:**
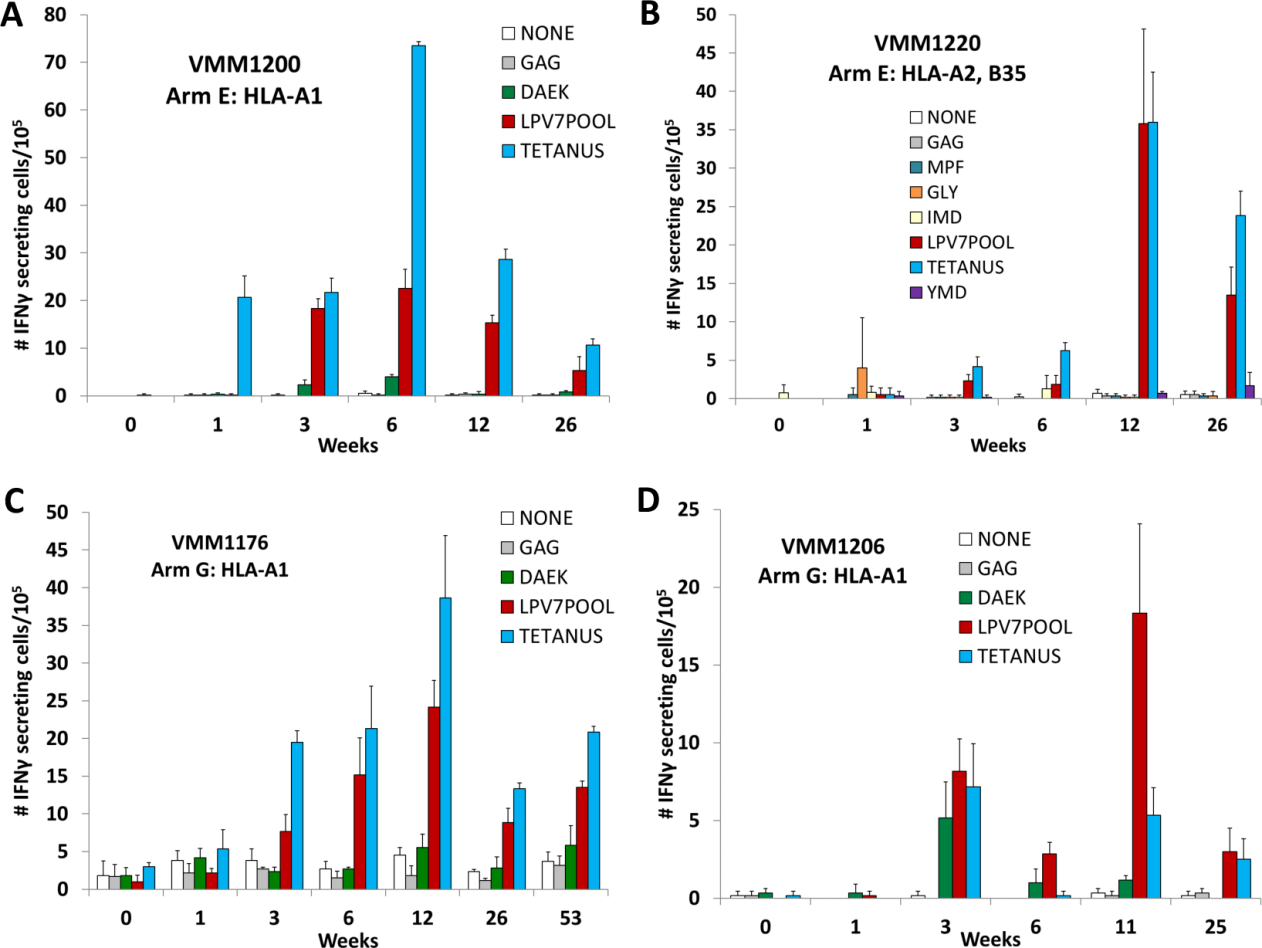
Examples of T cell responses by ex vivo ELIspot assay for two participants on arm E (A, B) and two on arm G (C, D). The number of cells secreting IFN-gamma in response to the pool of LPV7 peptides (LPV7POOL), Tetanus peptide, or individual MEPs (DAEK in A, C, D; MPF, GLY, IMD, YMD in B) are shown with bar graphs plus SD of replicate values. White and gray bars represent background (negative control) reactivity at each time point. The magnitude of response has not been adjusted for the per cent of CD8 T cells (for MEP) or for the per cent of CD4 T cells for tetanus peptide. MEP, minimal epitope peptide.

**Figure 3:**
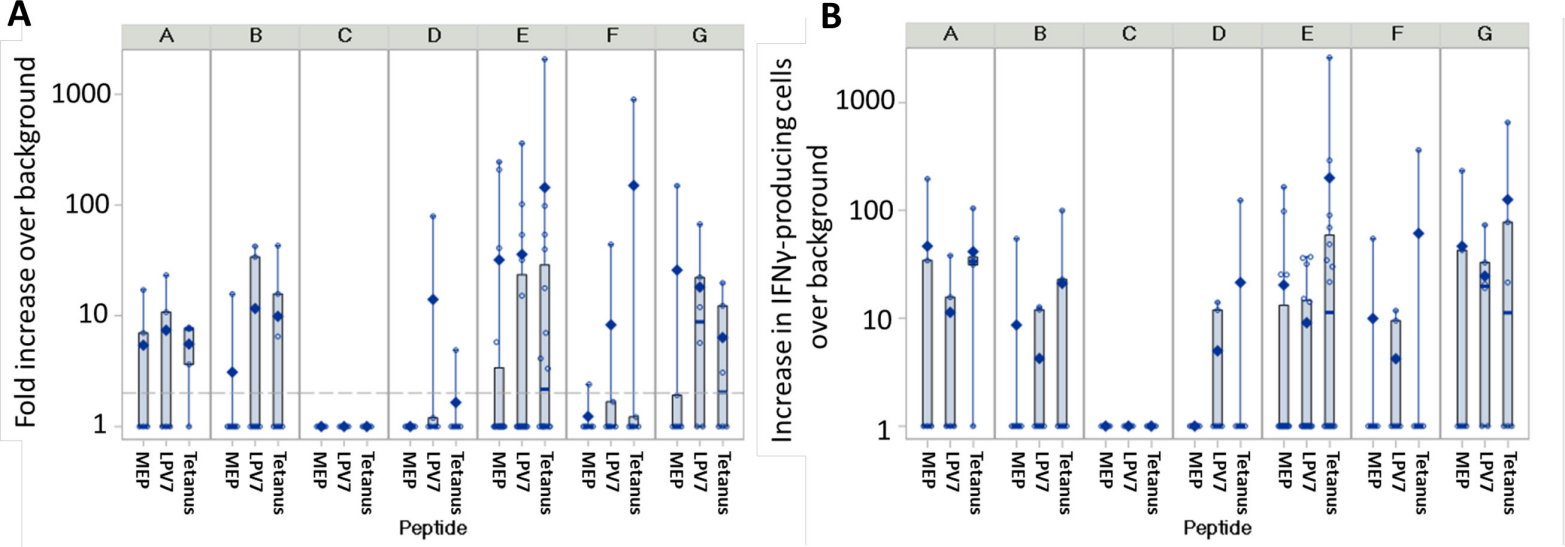
Summary T cell response data. The maximum T cell response for each patient, by study arm shown on the top of the panel, is shown in (A) as fold-increase over background, and in (B) as increase in IFN-gamma-producing T cells per 100,000 CD8^+^ T cells for short peptides (MEP), per 100,000 total PBMC for long peptides (LPV7), and per 100,000 CD4^+^ T cells for tetanus peptide (Tet). In (B), the values are modified by adding 1 to enable plotting zero values as 1 on a log scale. Bottom (25th percentile) and top (75th percentile) of the box represents the IQR, the thick horizontal line inside represents the median value. The diamond represents the mean, and the vertical line extends to the minimum and maximum observations outside of the IQR. The dashed reference line in panel A) indicates the twofold threshold for positivity. MEP, minimal epitope peptide.

By ex vivo ELIspot assay, immune responses to one of the seven long peptides individually or to the LPV7 pool, were observed in 15 subjects (30%, 90% CI 19 to 42). Example data for four participants are shown in figure 2, with some persisting to the last time point tested (figure 2B, C). The best IRRs to LPV7 were 67% (G), 40% (A), and 31% (E), with wide CIs (table 2). Among seven patients in whom T cell responses were detected to one or more of the individual long peptides, the most immunogenic were gp100 _9-39_ and NY-ESO-1 _79-108_ (5 and 3, respectively, table 2, right-most column). The peak T cell responses to LPV7 are summarized for each study arm in figure 3.

**Table 2:** T cell immune response rates

		Study group									
		A	B	C	D	E	F	G	A, E, F, G	B, C, D	A–G
Adjuvants		IFA	pICLC	Resiq	pICLC +resiq	IFA +pICLC	IFA +resiq	IFA +pICLC + resiq	Any IFA	No IFA	All
N		5	7	4	6	16	6	6	33	17	50
LPV7 pool	#+	2	1	–	1	5	1	4	12	2	14
Tyr (231-259)	#+	1	–	–	–	–	–	–	1	0	1
MAGE-A10 (245-274)	#+	1	–	–	–	–	–	–	1	0	1
Tyr (361-390)	#+	–	–	–	–	–	–	–	0	0	0
MAGE-A1 (82–111)	#+	–	–	–	1	1	–	–	1	1	2
Gp100 (9-39)	#+	1	2	–	–	2	–	–	3	2	5
NY-ESO-1 (79-108)	#+	1	–	–	–	1	–	1	3	0	3
Any long peptide	#+	1	2	0	1	2	0	1	4	3	7
LPV7 or any long peptide	#+	2	2	0	1	5	1	4	12	3	15
LPV7 pool or any	%+	**40**	**29**	**0**	**17**	**31**	**17**	**67**	**36**	**18**	**30**
(90% CI)		8 to 81	5 to 66	0 to 53	1 to 58	13 to 55	1 to 58	27 to 94	23 to 52	5 to 40	19 to 42
CD8 epitopes (MEP)	#+	**2**	**1**	**0**	**0**	**4**	**1**	**1**	**8**	**1**	**9**
CD8 epitopes (MEP)	%+	**40**	**14**	**0**	**0**	**25**	**17**	**17**	**24**	**6**	**18**
90% CI		8 to 81	1 to 52	0 to 53	0 to 39	9 to 48	1 to 58	1 to 58	13 to 40	0 to 25	10 to 29
Tetanus (CD4)	#+	**4**	**3**	**0**	**1**	**8**	**1**	**3**	**16**	**4**	**20**
Tetanus (CD4)	%+	**80**	**43**	**0**	**17**	**50**	**17**	**50**	**48**	**24**	**40**
90% CI		34 to 99	13 to 77	0 to 53	1 to 58	28 to 72	1 to 58	15 to 85	33 to 64	8 to 46	28 to 53

For the rows marked #+, the values are the number of patients positive for T cell response to the specified antigen(s), whereas for those marked %+, the values represent the % of patients in that group with an immune response. The rows with summary totals of patient numbers and proportions of the study population with immune responses are shown in bold.

### CD8 T cell response to MEPs epitopes

Each of the seven long peptides contained an MEP for CD8 T cells restricted by HLA-A1, A2, A3, B35, or B51 (table 1). T cell responses to these MEPs were assessed singly or as peptide pools by ex vivo ELIspot assay for each patient with the appropriate HLA expression. Example data for a T cell response to the MEP tyrosinase_240-251S_ (DAEK) are shown in figure 2D; however, the data in that plot represent raw values before adjusting for the percent of CD8^+^ T cells in the PBMC. After that adjustment, there were 56.2 IFN-gamma-secreting cells per 100,000 CD8^+^ T cells, with negative control values of 3.0 (not shown). Overall, CD8^+^ T cell responses were detected to MEPs in 9 patients (18%, 90% CI 10 to 29) with the best IRRs of 40% and 25% in arms A and E, respectively (table 2). The magnitude of those T cell responses to MEPs is shown by arm in figure 3. Among the nine participants with responses to MEPs, six had responses both to individual peptides and to the MEP pool, two had responses just to the pool, and one responded to an individual peptide. Responses to individual peptides were detected to tyrosinase _240-251S_, NY-ESO-1 _94-102_, gp100 _17-25_, and MAGE-A1 _96-104_ in four, two, one, and one patient, respectively, which represent 19%, 17%, 5%, and 5% of participants with the corresponding Class I MHC. In a prior trial using 12 MEPs+Tet+IFA in 41 participants (Mel44 trial, NCT00118274), CD8^+^ T cell responses were detected ex vivo to six of the MEPs using the same assay criteria. The IRRs to those six peptides in the prior study ranged from 4% to 78%, but IRRs to those same peptides in the present study ranged from 0% to 19%. Differences between IRR to these peptides between the studies ranged from 4% to 78% (online supplemental table 4).

### Helper T cell response to tetanus peptide

Immune responses to the Tet peptide provide another measure of immunogenicity with each adjuvant preparation. Example data for responses in four patients are shown in figure 2. Overall, responses were detected in 20 subjects (40%, 90% CI 28 to 53), with the highest IRRs of 80% in Arm A, 50% in Arms E and G, and 43% in Arm B (table 2). Data for immune response magnitude are summarized in figure 3.

### Immune response rates associated with adjuvant components

**Figure 4:**
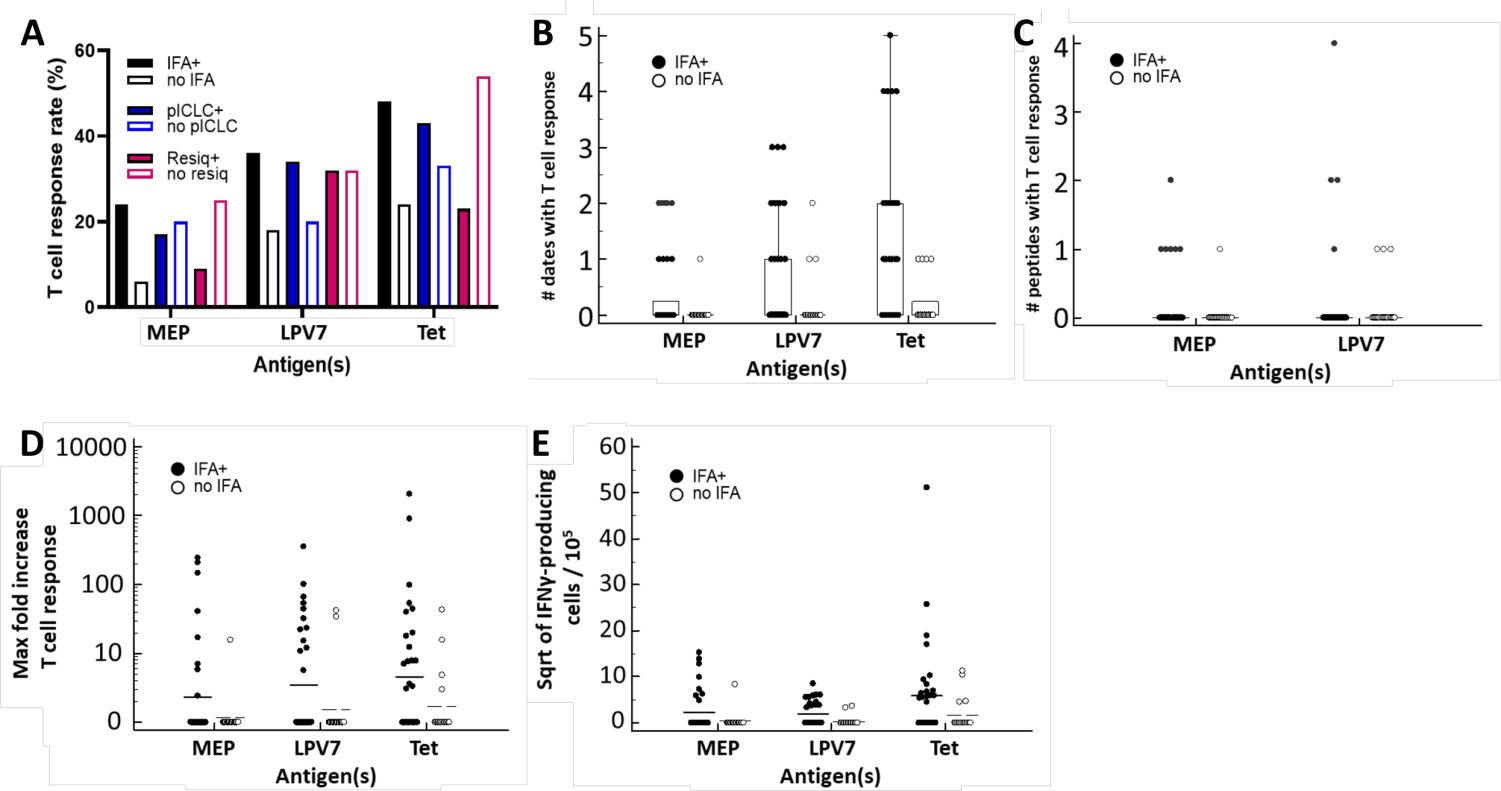
Immune responses after vaccination with adjuvants containing IFA or note containing IFA. The overall T cell response rates to MEPs, LPV7, and Tet are shown for groups based on the adjuvant used (A). Also, panels B–E show for participants vaccinated with IFA-containing adjuvants (groups A, E, F, G; n=33) and participants vaccinated with adjuvants lacking IFA (groups B-D; n=17), the per cent with immune responses to short peptides, LPV7, and Tet are shown (A), as well as the number of dates per participant with an immune response (B), number of peptides with immune response per participant (C), maximum fold increase (D), and the square root of the maximum number of IFN-gamma-secreting cells per 10^5^ (E). MEP, minimal epitope peptide.

This study was not designed to make comparisons among arms, but an exploratory assessment shows that the overall proportion of patients with T cell responses to MEPs, LPV7, and Tet trended higher for adjuvants containing IFA (arms A, E, F, G, n=33) than for those without IFA (IFA (arms B-D, n=17, table 2 and figure 4A). Adjuvants containing polyICLC yielded IRRs trending slightly higher than those without polyICLC for LPV7 and tetanus, but not for the short peptides. There was a trend to lower IRRs with adjuvants containing resiquimod versus those without resiquimod (figure 4A). For participants receiving IFA-containing vaccines, T cell responses were detected at more time points during the study (figure 4B), to more peptides (figure 4C), and with higher fold-increase and absolute magnitude of T cell response (figure 4D–E).

### Concordance of T cell responses to MEPs, LPV7, and Tet

Overall, 26 participants (52%) had immune responses to short peptides, LPV7, and/or Tet. Of the nine with T cell responses to short peptides, five (56%) also had responses to LPV7, and seven (78%) also had responses to Tet. Of 15 with responses to LPV7, 5 had responses to the short peptides and 10 had responses to Tet.

### Associations of T cell responses with treatment-related adverse events

As shown in online supplemental figure 1A, participants with higher grade 2/3 TRAEs had greater magnitude T cell responses to LPV7 (p=0.014), Tet (p=0.022), and MEP (p=0.068) by Fisher’s exact test. Also, grade 2/3 ISR (vs grade 0/1) was associated with higher immune response to MEP, LPV7, and Tet (p=0.008, 0.002, 0.009, respectively; Online supplemental figure 1B). Grade 2 induration at injection sites (vs 0/1) was associated with higher magnitude T cell responses to LPV7 (p=0.001) and to Tet (p=0.002), but not to MEPs (p=0.4) (online supplemental figure 1C).

### Antibody responses to LPV7

**Figure 5:**
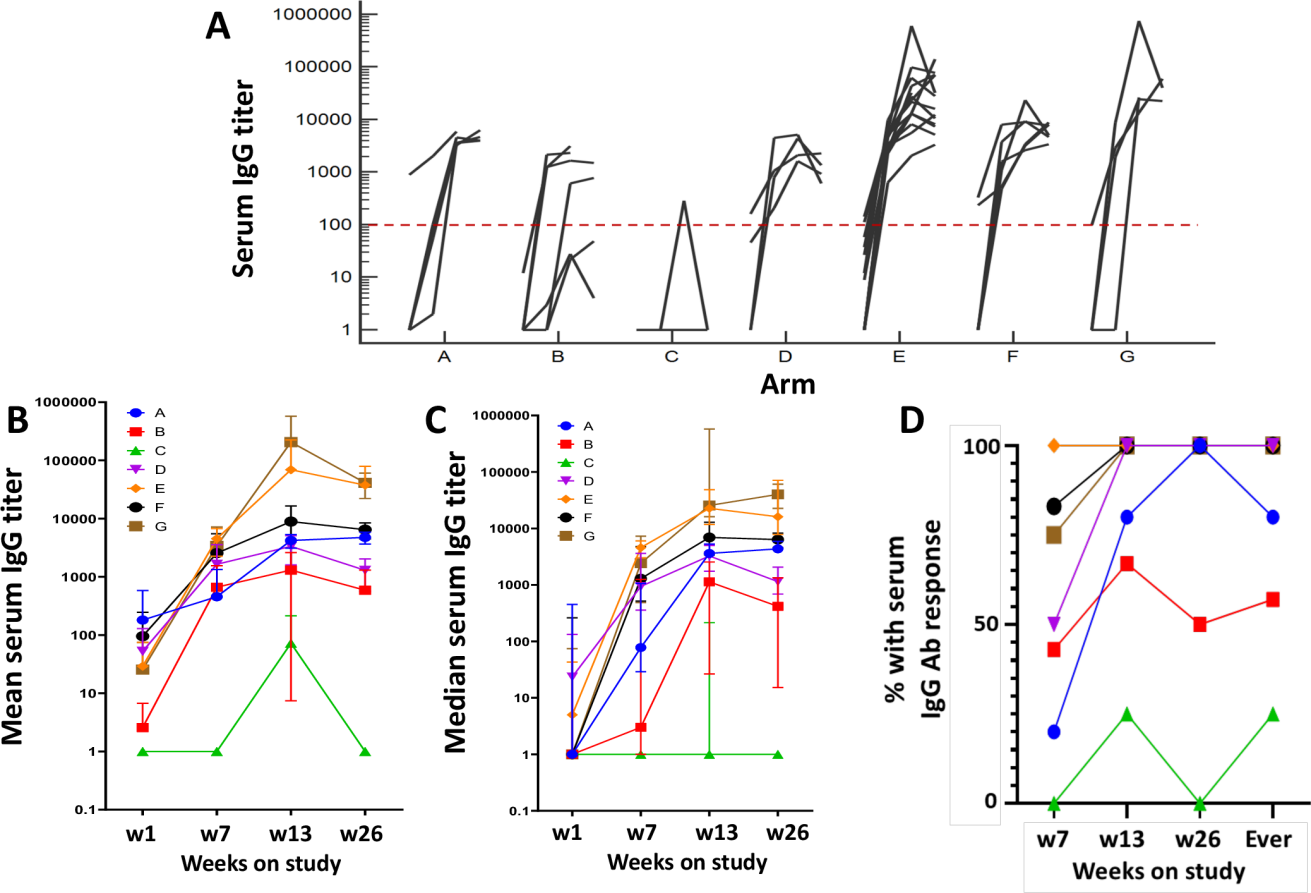
Serum IgG antibody response to LPV7 peptides. Serum Ab titers over time are shown for participants in arms A–G, representing 5, 7, 4, 4, 14, 6, and 4 participants, respectively (A). Also, the mean±SD (B) and median plus IQR (C) values for each arm are plotted over time. Panel (D) shows the cumulative proportion in the study with an Ab response by each time point.

IgG Ab responses to LPV7 were evaluated in 44 of the 50 participants. The induced IgG titers were highest in arms E and G and lowest in B and C (figure 5A–C). The proportions of participants with serum IgG response by week 7 were 0%, 20%, 43%, 50%, 75%, 83%, and 100% for arms C, A, B, D, G, F, and E, respectively, and increased by week 26%–100% for arms D–G, 80% for arm A, 57% for arm B, 25% for arm C, and 84% overall (figure 5D). For IFA-containing adjuvants, Ab responses were observed in 79% by week 7 and 97% overall. On the other hand, for adjuvants without IFA, Ab responses were observed in 33% by week 7% and 60% overall. IgG responses to Tet were also observed for 12/44 evaluable subjects (27%), including 75% (3/4) on arm G, 50% (7/14) on arm E, and one each on arms D and F (data not shown). IgG responses to HIV gag were assessed as negative controls with 42/44 (95%) negative and only 2 having low level titers (max 808 and 162) that presumably represent false positives (data not shown).

### T cell responses to peptides by enrolling institution

The overall immune response evaluation was performed for the full dataset in accord with the study design. However, differences were noted in T cell immune response by institution, details for which are in online supplemental table 5. All T cell responses to LPV7 and to MEP were observed for participants treated at Institution 1, the site where immune analyses were performed. Most responses to Tet were also observed at Institution 1, but some were observed at Institution 2. The proportions with T cell responses by institution are shown in online supplemental figures 2A, B. Blood samples were shipped overnight from Institution 2 to Institution 1; thus, the possibility that shipping conditions may have reduced T cell viability and function was explored. Controls for each analysis included response to two mitogens (phytohemagglutinin (PHA) and phorbol myristate acetate (PMA) plus ionomycin) as well as a pool of over 30 short peptides from viral proteins (CMV, EBV, influenza) restricted by Class I MHC molecules (CEF peptides). The number of cells producing IFN-gamma in response to CEF peptides varied widely among participants, which is expected, but ranges appear similar between institutions (online supplemental figure 2C, D). PMA responses also were similar between sites: p=0.93 (median 1534 vs 1427 per 100,000). However, the responses to PHA for Institution 2 were significantly lower than for Institution 1 (p=0.027; median 181 (95% CI 129 to 231)) vs 265 (214 to 326) (online supplemental figure 2C, D). Interestingly, the serum IgG (antibody) response to the LPV7 peptides was high for both sites, without appreciable differences in response frequency or magnitude (online supplemental figure 2E–G). Thus, participants at both institutions did receive vaccines and developed immune responses.

We had performed two prior multicenter clinical trials of melanoma vaccines incorporating 12) Class I MHC-restricted peptides (12MP) plus Class II MHC-restricted peptides (either Tet or six melanoma helper peptides, 6MHP):Mel43^37^ and Mel44.^15^ To explore whether this difference in IRR by institution had been observed previously, we reviewed the data on IRRs as a function of institution and found that in both of those trials, the IRRs were comparable across all sites, including the two institutions participating in the current trial (online supplemental figure 3).

### Clinical outcomes

With a median follow-up of 4.7 years, 43 (86%) of patients remain alive, with 32 (64%) without known disease recurrence. For arms with IFA-containing adjuvants (A, E, F, G), recurrences have occurred in 11/33 (33%, 90% CI 20 to 49), vs 7/17 (41%, 90% CI 21 to 64) in those without IFA. Deaths due to melanoma have occurred in 3/33 with IFA (9%, 90% CI 3 to 22) vs 4/17 (24%, 90% CI 8 to 46) without IFA (arms B, C, D). Kaplan-Meier plots for disease-free and overall survival in the whole study population are shown in online supplemental figure 4.

## Discussion

This study was initiated to assess safety and immunogenicity of vaccination with long peptides with each of seven adjuvant combinations and to select an adjuvant combination to use in future studies. LPV7 plus Tet was well-tolerated across all adjuvant combinations with only one DLT. All other AEs were expected and consistent with prior vaccine studies. Ulceration at the vaccine site was identified in three participants (6%) which is lower than with prior trials using short peptides and more than with helper peptides.^38^ There were significant positive associations between IRR and severity of local and overall TRAEs (online supplemental figure 1), suggesting that the extent of immune response may enhance inflammation locally and systemically.

A rationale for using long peptides is the requirement for internalization and presentation by APC, avoiding tolerogenic potential of MEPs binding directly to Class I MHC on cells such as fibroblasts and keratinocytes.^9^ Also, in vaccines containing IFA in mice, long peptides escape the negative effects of chronic inflammation in the vaccine site microenvironment (VSME) that may diminish immunogenicity of MEPs.^8^ In prior clinical trials with 12MP plus Tet in IFA alone (Mel43 (n=60) and Mel44 (n=41)), we observed CD8^+^ T cell responses ex vivo to at least one peptide by ELIspot assay in 73% and 78% of evaluable patients, respectively.^14 15^ CD8^+^ T cell responses to MEPs in the present trial did not exceed those IRRs, with only 18% overall, including 24% of all 33 participants receiving IFA as part of the adjuvant. The six peptides selected from 12MP included the most immunogenic in the prior Mel44 trial (online supplemental figure 4).^15^ The CD8 T cell response rates to each of the six MEPs from 12MP are lower than expected from the Mel44 trial (). Thus, lower CD8 T cell responses to the MEPs may be due to factors other than the choice of peptide and may be due to any of several factors:

- The present trial differed from the prior trials by administering vaccines 4–6 every 3 weeks, rather than weekly. However, most immune responses are evident after the first three vaccines; so, this schedule change is not likely to explain the difference. Also, high IRRs have been observed in other trials with vaccines 4–6 administered every 3 weeks.^39 40^
- In some of our prior trials, vaccines were administered to the same vaccine site each week (same site vaccination, SSV), but vaccines were rotated to different skin sites each week in the present trial. We have found that SSV can induce tertiary lymphoid structures and enhance Th1 dominance in the VSME.^41 42^ These may be crucial for optimal immunogenicity, and further investigation is underway to understand the role of SSV.
- Surprisingly, almost all detected T cell responses were observed in participants treated at Institution 1, where the immune analyses were performed. This difference may be explained partially by negative impacts of PBMC shipping overnight from Institution 2, but preserved responses to PMA and CEF peptides suggests that most T cell function was preserved. We have previously noted that temperature conditions during shipping can affect PBMC recovery.^43^ Exposure to 40°C for more than 8 hours, as is a common shipping occurrence from Institution 2, results in reduced cell recovery and cell viability. Within Institution 1 patients only, the IRRs to LPV7 were as high as 71% (5/7) for Arm E and 100% (4/4) for Arm G, and IRRs to MEPs were highest for Arm E (57%, 4/7), which are more favorable than for the whole study population. Even if only reviewing that data for Institution 1, the findings of favorable immunogenicity with IFA+polyICLC are supported and the more favorable immunogenicity with addition of IFA to TLR agonists overall (online supplemental figure 2A).

The IRRs to Tet exceeded 90% in prior trials;^14 15^ thus, the overall 40% IRR to Tet in the present trial, despite adding TLR agonists in most arms, is lower than expected and suggests a need for further optimization. Tet was added because it was not initially known if LPV7 alone would induce strong CD4^+^ T cell responses. The overall IRR to LPV7 may include both CD8^+^ and CD4^+^ T cell responses. These were observed ex vivo in 30% of participants overall. Serum Ab responses to LPV7 were detected in a much higher proportion of participants (84%). Now that we have data that LPV7 alone is sufficient to induce both CD8^+^ and CD4^+^ T cell responses, use of Tet may not be necessary going forward. Still, tetanus toxoid can modulate responses in the VSME and help support immune responses to less immunogenic melanoma-specific peptides.

The lack of detectable T cell responses in samples from Institution 2 may reflect artifactual lack of reactivity there, and it raises the possibility that the T cell response rates at Institution 1 are more reflectively of the true T cell response rates. If so, then the overall IRR to LPV7 may be closer to 48% overall, and to 67% for those patients vaccinated with IFA-containing adjuvants, as shown for Institution 1 in online supplemental table 5. Similarly, the CD8 T cell response rate to MEP after vaccination with LPV7 in IFA containing adjuvants may be closer to the 44% observed at Institution 1. Supporting this is the finding of T cell responses to Tetanus peptide in 72% of patients vaccinated with IFA-containing adjuvants at Institution 1, which is closer to what has been observed in other trials.

We have previously demonstrated that Ab responses to melanoma helper peptides are associated with induction of T-cell responses and with improved overall survival.^44^ Since Abs will not bind directly to antigen on tumor cells but rather to epitopes of intracellular antigens presented in the context of MHC, they may opsonize the vaccine peptides to enhance DC presentation and activation of T cells. Abs can also opsonize intracellular proteins after cell death and support cross-presentation of proteins released by dying tumor cells. More work is being done in this area to understand this phenomenon and optimize the application of immune monitoring using Ab responses.

Among the monotherapy adjuvant strategies (Zone 1, online supplemental table 1), T cell IRRs to LPV7 were higher with IFA alone than with either TLR agonist alone. For vaccines with doublet adjuvants (Zone 2), the highest IRR was with LPV7 with IFA plus polyICLC. With all three adjuvants (Arm G), T cell responses to LPV7 were detected in four of six participants (67%) but with only one demonstrable CD8 T cell response to MEPs. Across all cohorts, those including IFA induced IRRs for T cells and Ab that trended higher than those lacking adjuvant combinations lacking IFA (figure 5 and table 2). Vaccines including IFA also were associated with favorable clinical outcomes thus far.

This trial used a novel adaptive design that enabled assessment of safety and immunogenicity across seven different vaccine adjuvants with lower enrollment than would be required if it were a traditional 7-arm randomized study. The adaptive design limited exposure of participants to less immunogenic regimens. However, the small number of participants enrolled in most arms limits the precision of the immune response estimates. Arm E enrolled the most participants (16) and was among the most immunogenic approaches. Arm G also generated favorable IRRs, but it is not clear that adding resiquimod significantly enhances immunogenicity over the regimens without resiquimod. The evaluation of adjuvants containing IFA versus those without IFA is an unplanned analysis and may be considered exploratory only. However, it supports continued use of IFA as an adjuvant for peptide vaccines in humans, contradicting a trend based on murine data. The present study did not evaluate agonists for TLR9, which have shown value for enhancing the immunogenicity of peptides when added to IFA.^19^ This and other agents also offer promise. Preclinical data also strongly support the use of CD40 antibodies plus TLR agonists administered directly with peptide vaccines,^45^ and this has not yet been tested in humans, but remains another promising strategy. In future studies, we would like to explore these approaches and analyses of additional cytokines as well as functional assays of non-IFN-gamma activity, chemokine receptors such as CXCR3, and other homing receptors such as cutaneous lymphocyte antigen and α4β7 integrin expression as demonstrated in previous work.^39 46^ Curiously, IRRs do not always correlate with intratumoral responses. There is very little information on whether T cells induced by cancer vaccines infiltrate tumors, The present study was performed in patients without measurable tumor deposits; so, those analyses were not feasible in the present study. Such studies are important to address in future studies that incorporate tumor biopsies prevaccination and postvaccination.

A major focus of current vaccine development is on mutated neoantigens, which show promise, but their activity is limited by lack of consensus on the best way to induce immune responses to these and other cancer antigens. Thus, there is a critical need to define the best vaccine adjuvants to use with these and other cancer vaccines. The present study contributes to an enhanced understanding and provides direction toward optimized cancer vaccine approaches by demonstrating safety and immunogenicity of seven new long peptides, some of which may be useful beyond melanoma, by confirming the safety and immunogenicity of vaccination with IFA+polyICLC, and by raising new questions about the role of same-site vaccination that deserve further investigation and may enhance the effectiveness of cancer vaccines.

## Data Availability

Data are available on reasonable request. All data relevant to the study are included in the article or uploaded as supplementary information. All data relevant to the study are included in the article or uploaded as supplementary information and are available on request to the senior author.
