# Supplementary material for: Phase I/II trial of a long peptide vaccine (LPV7) plus toll-like receptor (TLR) agonists with or without incomplete Freund’s adjuvant (IFA) for resected high-risk melanoma
Source: J Immunother Cancer. 2021 Aug 19;9(8):e003220. doi: 10.1136/jitc-2021-003220 (PMC8378357; doi:10.1136/jitc-2021-003220)
Supplement: Supplementary data [file jitc-2021-003220supp003.pdf]

PRC# 1143-10 / IRB-HSR#15931 / IND# 15891
Version Date: 07-31-17

UNIVERSITY OF VIRGINIA
HUMAN IMMUNE THERAPY CENTER

PHASE I/II TRIAL OF A LONG PEPTIDE VACCINE (LPV7) PLUS TLR AGONISTS FOR RESECTED STAGE IIB – IV MELANOMA

ATTACH TO THE FRONT OF EVERY COPY OF THIS PROTOCOL

Confidentiality Statement
The information contained in this document is the property of the University of Virginia Human Immune Therapy Center. Distributing, copying, or disclosing information contained in this document requires prior authorization from the Human Immune Therapy Center, except that this document may be disclosed to the appropriate institutional review boards and representatives of the U.S. Food and Drug Administration.

Patel SP, *et al. J Immunother Cancer* 2021; 9:e003220. doi: 10.1136/jitc-2021-003220

PRC# 1143-10 / IRB-HSR#15931 / IND# 15891
Version Date: 07-31-17

Signature Page

Sponsor's Representative

**Name**	**Signature**	**Date**

Investigator

**Name**	**Signature**	**Date**

PRC# 1143-10 / IRB-HSR#15931 / IND# 15891
Version Date: 07-31-17

UNIVERSITY OF VIRGINIA
HUMAN IMMUNE THERAPY CENTER

PHASE I/II TRIAL OF A LONG PEPTIDE VACCINE (LPV7) PLUS TLR AGONISTS FOR RESECTED STAGE IIB – IV MELANOMA

Principal Investigator (UVA): Craig L. Slingluff, Jr., M.D.
University of Virginia
434-924-1730
cls8h@virginia.edu

Principal Investigator (MDA) Sapna Patel, M.D.
The University of Texas MD Anderson Cancer Center
713-792-2921
sppatel@mdanderson.org

Co-Investigators: Patrick Hwu, M.D., Ph.D.
The University of Texas MD Anderson Cancer Center
713-563-1728
phwu@mdanderson.org

Merrick Ross, M.D.
The University of Texas MD Anderson Cancer Center
713-792-0025
mross@mdanderson.org

Elizabeth Gaughan, M.D.
Unviersity of Virginia
434-924-1904
emg5x@virginia.edu

William W. Grosh, M.D.
University of Virginia
434-924-1904
wwg9u@virginia.edu

Carmel Nail, F.N.P.
University of Virginia
434-982-4042
cjn2r@virginia.edu

Kathleen Haden, RN MSN ANP-C
University of Virginia
434-924-1730
km3s@virginia.edu

Patel SP, et al. J Immunother Cancer 2021; 9:e003220. doi: 10.1136/jitc-2021-003220

Walter C. Olson, Ph.D.
University of Virginia
434-243-9688
wco3j@virginia.edu

Kimberly A. Bullock, Ph.D.
University of Virginia
434-924-0180
kb9d@virginia.edu

Willem Overwijk, Ph.D.
The University of Texas MD Anderson Cancer Center
713-563-5294
WOverwijk@mdanderson.org

Biostatisticians:

Gina R. Petroni, Ph.D.
University of Virginia
434-924-8363
grp4c@virginia.edu

Mark Smolkin, M.S.
University of Virginia
434-982-1032
mes6r@virginia.edu

Research Nurses:

Carmel J. Nail, F.N.P.
Kathleen Haden, RN MSN ANP-C

Clinical Research Coordinators and Data Management-University of Virginia

Emily Allred, PhD
434-982-1902
eh4m@virginia.edu

Jessica Zareno, MS
434-982-1901
jhz4f@virginia.edu

Alexandra Carney, MPH
434-982-6714
alc6a@virginia.edu

Kendall Pettaway, BS
434-982-6584
kp8ac@virginia.edu

Annika Shuali, MS
434-243-7242

ah2ze@virginia.edu

Sarah Lewis, BA
434-924-8709
set5v@virginia.edu

Authors: Craig L. Slingluff, Jr., M.D.
Jade Homsi, M.D. (Part 1)
Willem Overwijk, Ph.D. (Part 1)
Gina R. Petroni, Ph.D.
Kimberly A. Bullock, Ph.D.
Walter Olson, Ph.D.
Sapna Patel, M.D. (Part 1)

1.0 INTRODUCTION 14
Abstract 14
1.1 Study Rationale 14
1.2 Vaccine Design and Surgical Excision 16
2.0 STUDY OBJECTIVES 23
2.1 Part 1 (completed) 23
2.2 Part 2 24
3.0 STUDY OUTLINE 24
3.1 Type of Study 24
4.0 PARTICIPANT SELECTION 24
4.1 Inclusion Criteria 24
4.2 Exclusion Criteria 25
4.3 Demographics of Participant Population 27
4.4 Registration, Randomization, and Management of Participants 27
5.0 STUDY MEDICATION FORMULATION, DOSAGE, ACCOUNTABILITY AND ADMINISTRATION 28
5.1 Study Medication Formulation 28
5.2 Preparation, Dosage, and Storage of Study Drug 29
5.3 Drug Accountability and Distribution 31
5.4 Administration of Vaccines 31
5.5 Modification/Discontinuation of Treatment 33
5.6 Concomitant Medications 35
6.0 CLINICAL AND LABORATORY EVALUATION 36
6.1 Screening 36
6.2 Treatment 37
6.3 Tumor tissue collection 41
7.0 REGULATORY AND REPORTING REQUIREMENTS 41
7.1 Definitions 42
7.2 Attribution Assessment 43
7.3 Data collection 44
7.4 Risks and Safety 44
7.5 Adverse Event Classifications 47
7.6 Reporting Adverse Events 49
7.7 Adverse Event Review and Monitoring 52

7.8 Adverse Event Stopping Guidelines 53
7.9 Responsibility 54
7.10 Endpoint Data 54
8.0 EVALUATION OF IMMUNOLOGIC RESULTS **54**
8.1 Immunohistochemistry and Immunofluorescence of skin biopsy specimens 55
8.2 ELIspot assay 55
8.3 Tetramer assay and multiparameter flow cytometry 56
8.4 Proliferation assay / Cytokine Analysis 56
8.5 Antibody responses 56
8.6 Evaluation of tumor 57
9.0 STATISTICAL CONSIDERATIONS **57**
9.1 Study design Part 1 (completed) 57
9.2 Combination allocation Part 1 (completed) 58
9.3 Statistical properties Part 1 (completed) 60
9.4 Study design Part 2 61
9.5 Sample size/accrual 61
9.6 Stopping rules: 62
9.7 Analyses 63
10.0 Appendices **64**
10.1 Appendix 1: X-page 65
10.2 Appendix 2: AJCC Staging System, 7th Edition 66
10.3 Appendix 3: ECOG Performance Status 68
10.4 Appendix 4: New York Heart Association Disease Classification 69
10.5 Appendix 5: Lot Testing 70
10.6 Appendix 6: NCI Common Terminology Criteria for Adverse Events v4.03 72
10.7 Appendix 7: Summary of Changes 73
11.0 Reference List **78**

Protocol Precis

Title: Phase I/II trial of a long peptide vaccine (LPV7) + TLR agonists for resected stage IIB-IV melanoma

Objectives:
Part 1 (completed)
(1) To test safety of vaccination with a mixture of long peptides for patients with high-risk melanoma, in each of 7 adjuvant preparations, including TLR agonists and/or incomplete Freund's adjuvant (IFA)
(2) To estimate the immunogenicity of 7 long peptides in each of 7 adjuvant preparations

Exploratory objective:
(3) To evaluate the molecular and cellular changes at the vaccine site and systemically

Part 2
(4) To assess safety of vaccination of the optimal combination identified in Part 1 when administered in the same skin site for all 6 vaccines (same site vaccination).
(5) To estimate the immunogenicity of the optimal combination identified in Part 1 when administered in the same skin site for all 6 vaccines (same site vaccination).

Design: This is an open-label, randomized, phase I/II study, using an adaptive design, of the safety and immunogenicity of a novel melanoma vaccination approach using long peptides plus TLR agonists, along with characterization of cellular and molecular events at the cutaneous sites of immunization and systemically.
This trial involves two parts, which differ in the location of vaccine injections. For part 1, each vaccine was administered in a skin site that differs on each vaccine date (rotating vaccine sites). For part 2, each vaccine will be administered in the same skin site for all 6 vaccines (same site vaccination).

Table 1A. Clinical Trial Design for Part 1

Zone	Study Arm	Peptide vaccine	Adjuvant	Dose of TLR agonist	Route vax injection	Admin of TLR agonist
1	A	LPV7 + tet	IFA	---	Id/sq	--
1	B	LPV7 + tet	PolyICLC	1 mg	Id/sq	Id/sq
1	C	LPV7 + tet	Resiquimod	1000 mcg	Id/sq	Topical
2	D	LPV7 + tet	PolyICLC + resiquimod	1 mg/ 1000 mcg	Id/sq	Id/sq (PolyICLC) & topical (resiquimod)
2	E	LPV7 + tet	IFA + PolyICLC	1 mg	Id/sq	Id/sq
2	F	LPV7 + tet	IFA + resiquimod	1000 mcg	Id/sq	Topical
3	G	LPV7 + tet	IFA + PolyICLC + resiquimod	1 mg/ 1000 mcg	Id/sq	Id/sq (PolyICLC) & topical (resiquimod)

For part 2, the vaccine regimen is selected based on available data and the study decision rules from Part 1 which was determined to be Arm E. For Part 2, accrual will be to Study Arm E2. However, a modification is made to exclude the tetanus helper peptide, due to (a) lack of availability of that peptide component, (b) the finding that, in part 1, LPV7 peptides do

induce helper T cell responses themselves (unpublished data), and prior data that adding the tetanus helper peptide did not add significantly to immunologic or clinical outcome of a peptide vaccine designed to induce CD8 T cells[1].

Table 1B. Clinical Trial Design for Part 2

Zone	Study Arm	Peptide vaccine	Adjuvant	Dose of TLR agonist	Route vax injection	Admin of TLR agonist
2	E2	LPV7	IFA + PolyICLC	1 mg	Id/sq	Id/sq

Regimen: Patients will be registered on this phase I/II trial and will be vaccinated with LPV7 at 300 mcg peptide/dose every week x 3 then every 3 weeks x 3.

For Part 1, the adjuvants are described, and will be administered to patients in 7 study groups as shown in Table 1A. This incorporates an adaptive design with sequential initiation of accrual to study Zones with increasing numbers of adjuvants (Arms A-C, then D-F, then G). Details are in Section 9.0 and Appendix 1 (X-page).

For Part 2, the adjuvants will be administered to patients in 1 study group as shown in Table 1B.

Vaccine site biopsies: In each patient, three 4-mm punch biopsies will be performed of the skin at vaccine sites at two time points: 1 week after vaccine #1 (day 8) and one week after vaccine #3 (day 22). As negative controls, 3 punch biopsies will be obtained of clinically normal skin (not at the vaccine site) in the first 6 patients in Part 1 at the same time points (days 8 and 22). Details are provided in section 5.4.6.2.

Leadership: This trial will be performed at two institutions in patients with high-risk resected stage IIB-IV melanoma. The lead institution for this trial will be the University of Virginia, and the PI will be Dr. Craig Slingluff. The institutional PI at M.D. Anderson Cancer Center will be Dr. Patrick Hwu. Part 2 will be conducted at the University of Virginia only.

Population: Criteria for inclusion include:

- age 18 years or older
- expression of HLA-A1, -A2, -A3, -B35, or -B51
- histologically or cytologically proven Stage IIB-IV melanoma rendered clinically free of disease by surgery, other therapy, or spontaneous remission. Small (<1cm) findings that are equivocal on radiologic or clinical imaging will not be a basis for exclusion.
- ECOG performance status 0-1 (Appendix 3)
- Adequate hepatic and renal function
- Weight at least 50 kg (110 lbs)

NOTE: Patients will be excluded if they are pregnant or are immunosuppressed, or have previously received vaccines containing an incomplete Freund's adjuvant.

Accrual: For Part 1, the maximum accrual to the study is estimated from the simulations to be approximately 52 patients. Adjusting for 10% ineligibility/dropout/withdrawal rate, maximum total target accrual is estimated at 58 patients. For Part 2, target accrual is estimated to be 16 eligible patients. Adjusting for 10% ineligibility/dropout/withdrawal rate, maximum total accrual for part 2 is estimated at 18 patients.

Clinical outcome: The primary goals are to assess safety and immunogenicity. The study is not powered for differences in clinical outcome, but we will track disease-free survival, and overall survival. Significant differences in clinical outcome are not anticipated among the study arms, but trends in clinical outcome will be assessed in the context of the immunologic data to support decisions about the preferred vaccination strategy to test further in combination with other immune therapies.

Vaccine composition: The peptides described in Table 2 will be used in the vaccines.

Table 2: 7 long Melanoma Peptides Restricted by MHC Class I and the corresponding defined minimal epitope

	**Short Peptides**	**Long Peptide (30-mers) in LPV7 for the present proposal**	
**Allele**	**Minimal Epitope**	**Sequence (minimal epitope underlined)**	**Source (# residues)**
HLA-A1	Tyrosinase $_{240-251S}$*	FTIPYWDWRDAEKSDICTDEYMGGQHPTN	Tyrosinase $_{231-259\ S}$* (29)
HLA-A2	Tyrosinase $_{369-377}$ ♦	SMHNALHIYMDGTMSQVQGSANDPIFLLHH	Tyrosinase $_{361-390}$ ♦ (30)
	gp100 $_{209-217-2M}$ #	VPLAHSSSAFTIMDQVPFSVSVSQLRALDG	gp100 $_{198-227}$ # (30)
	MAGE-A10 $_{254-262}$	VIWEALNMMGLYDGMEHLIYGEPRKLLTQD	MAGE-A10 $_{245-274}$ (30)
HLA-A3	gp100 $_{17-25}$	LLHLAVIGALLAVGATKVPRNQDWLGVSRQL	gp100 $_{9-39}$ (31)
	MAGE-A1 $_{96-104}$	SREEEGPSTSCILESLFRAVITKKVADLVG	MAGE-A1 $_{82-111}$ (30)
HLA-B35/B51	NY-ESO-1 $_{94-102}$	GARGPESRLLEFYLAMPFATPMEAELARRS	NY-ESO-1 $_{79-108}$ (30)

*(substitution of S for C at residue 244); ♦(post-translational change of N to D at residue 371); #(209-2M, substitution of M for T at position 210)

Endpoints:

Primary:

- Safety and toxicity following vaccination with 7 long peptides in melanoma patients with and without TLR agonists
- Levels of peptide-reactive $CD8^+$ T cells in the peripheral blood (peak and durable at 26 weeks) and vaccine site

Secondary:

- $CD4^+$ T cell responses to peptides in the vaccine, and their function.

Exploratory:

- To analyze cellular infiltrates in the VSME
- To identify cytokine profiles of the VSME as a function of TLR signaling
- To identify toll-like receptor signaling in the VSME
- To understand regulatory processes in the replicate immunization sites
  - Regulatory T cells ($CD4^+CD25^{hi}FoxP3^+$)
  - Myeloid-derived suppressor cells
- Overall Survival and disease-free survival

List of Abbreviations

Abbreviation	Full text
βHCG	Beta Human chorionic gonadotropin (pregnancy test)
12-MP	12 melanoma-derived class I MHC-restricted peptides
AE	adverse event
AGC	absolute granulocyte count
AJCC	American Joint Committee on Cancer
ALT	alanine aminotransferase
ANC	absolute neutrophil count
APC	antigen presenting cell
AST	aspartate aminotransferase
BTRF	Biorepository and Tissue Research Facility
CC	Cancer Center
cc	cubic centimeter
CFR	Code of Federal Regulations
cm	centimeter
CO_2	carbon dioxide
CRF	case report form
C3TO	Cancer Center Clinical Trials Database
CT	computed tomography
CTA	cancer-testis antigens
CTCAE	Common Terminology Criteria for Adverse Events
CTL	cytotoxic T lymphocyte
CTO	Clinical Trials Office
DC	dendritic cells
DFS	Disease-free survival
dL	deciliter
DLT	dose limiting toxicity
DSMC	Data and Safety Monitoring Committee
ECOG	Eastern Cooperative Oncology Group
ELISA	enzyme linked immunosorbent assay
FACS	fluorescence activated cell sorter
FBS	fetal bovine serum
FDA	Food and Drug Administration
FITC	Fluorescein isothiocyanate
g	gram
GLP	good laboratory practice
GM-CSF	granulocyte-macrophage colony-stimulating factor
GMP	good manufacturing practice
Hgb	hemoglobin
HGBA1C	hemoglobin a1c
HITC	Human Immune Therapy Center
HIV	human immunodeficiency virus
HLA	human leukocyte antigen
HPLC	high performance liquid chromatography
HR	Hazard ratio
IBW	ideal body weight
id	intradermal

IFA	incomplete Freund's adjuvant
IFN	interferon
IHC	immunohistochemistry
IL-2	interleukin-2
IL-7	interleukin-7
IL-15	interleukin-15
IML	Immune Monitoring Laboratory
in	inch
IND	investigational new drug
ip	intraperitoneal
IRB	Institutional Review Board
IRR	immunologic response rate
IU	international unit
IV	intravenous
kg	kilogram
KLH	keyhole limpet hemocyanin
LC	Langerhans cells
LDH	lactate dehydrogenase
LNLA	Lymph-node-like aggregate
LPV7	Long peptide vaccine with 7 melanoma peptides
LyNeo	lymphoid neogenesis
m	meter
mcCi	microcurie
mcg	microgram
mcl	microliter
MDACC	M.D. Anderson Cancer Center
MDP	melanocyte differentiation proteins
mg	milligram
MHC	major histocompatibility complex
mIU	million international units
ml	milliliter
mm	millimeter
MRI	magnetic resonance imaging
NBT/BCIP	Nitro blue tetrazolium chloride/5-Bromo-4-Chloro-3-Indolyl Phosphate
NED	No evidence of disease, i.e. no clinical evidence of melanoma
NCI	National Cancer Institute
NOS	not otherwise specified
NSAID	non-steroidal anti-inflammatory drug
OS	overall survival
PBL	peripheral blood lymphocytes
PBMC	peripheral blood mononuclear cells
PBS	phosphate buffered saline
PET	positron emission tomography
PHA	phytohemagglutinin
PI	Principal Investigator
PMA	phorbol myristate acetate
PolyICLC	polyinosinic-polycytidylic acid stabilized with carboxymethylcellulose, and poly-L-lysine double-stranded RNA

ppm	parts per million
PRC	protocol review committee
RCC	renal cell carcinoma
RPMI	Roswell Park Memorial Institute
SD	standard deviation
SIN	sentinel immunized node
SQ	subcutaneous
TAA	tumor associated antigens
tet	tetanus helper peptide
TFA	trifluoroacetic acid
T_h	$CD4^+$ helper T cells
TIL	tumor infiltrating lymphocytes
TLR	Toll-like receptor
TNF	tumor necrosis factor
ULN	upper limits of normal
USP	United States Pharmacopeia
UVA	University of Virginia
VSME	Vaccine site microenvironment
WBC	white blood cell
w/v	weight to volume
v/v	volume to volume

1.0 INTRODUCTION

Abstract
Identification of minimal peptide epitopes for melanoma reactive T cells in the early 1990s has provided useful tools for evaluating T cell responses to melanoma; however, the immune responses to vaccines using short peptides are often transient and of low magnitude, and they have had limited clinical activity. Recent work suggests that vaccination with longer (30-mer) peptides that encompass these short peptides may be a more effective strategy and has been associated with clinical regressions of squamous vulvar neoplasia[1]. The most common adjuvant for peptide vaccines for melanoma has been a form of incomplete Freund's adjuvant (IFA). However, Toll-like receptor (TLR) agonists offer the potential to improve the magnitude and persistence of antitumor T cell responses, and findings from a few clinical trials support this potential[2-4]. However, there has been very limited characterization of the molecular and cellular effects of TLR agonists at the vaccine site microenvironment (VSME). A randomized trial of high-dose interleukin-2 (IL2) vs. IL2 + peptide vaccine showed improved response rate and progression free survival with addition of the peptide vaccine [5]. Other preclinical studies show improved tumor control by adding vaccines to adoptive T cell therapy and to blockade of immunologic checkpoints. An optimized vaccine strategy, relevant across the large range of HLA alleles is needed.

The proposal is for a clinical trial, performed in a collaborative multicenter setting involving 2 academic institutions. This trial will accrue approximately 52 eligible patients (Part 1) and approximately eligible 18 patients (Part 2) in a phase I/II design with a long peptide vaccine (LPV7) in one of 7 adjuvant strategies of IFA alone, TLR agonist(s) alone, or a combination of IFA plus TLR agonist(s). Goals of this trial will include a safety assessment and measures of immunogenicity, and characterization of the cellular and molecular events induced by each TLR agonist in the VSME. This will be a first-in-humans evaluation of the safety and immunogenicity of 7 long 30-mer peptides (LPV7) for melanoma, which incorporate 7 well-characterized minimal epitopes restricted by HLA-A1, A2, and A3 (approximately 80% of melanoma patients) and a well-characterized epitope restricted by HLA-B35/B51. We propose to test these peptides in IFA alone, an adjuvant combining IFA with each of the following polyICLC/Hiltonol (TLR3 agonist), resiquimod (TLR7/8 agonist), and both polyICLC and resiquimod, to be administered locally at the site of vaccination.

1.1 Study Rationale

Cancer immunotherapy for solid tumors is coming of age, with FDA-approved immuno-therapeutics now available for the treatment of multiple tumor types. In melanoma, checkpoint inhibitors specific for CTLA-4 and PD-1 have been shown to induce durable clinical regressions and improve overall survival. There is excitement about the growing armamentarium of systemic immunotherapeutics, whose effects are mediated predominantly by T lymphocytes. However, despite the effectiveness of those therapies, checkpoint inhibitors fail in about 70-80% of patients, and these patients often succumb to their disease. There is a need for new combination approaches that build on the demonstrated clinical value of immune therapy.

Cancer vaccines inducing antigen-specific T cell responses are emerging as a component of combination immunotherapy. In the past few years, a cancer vaccine has been approved for prostate cancer, based on two randomized trials showing improved

survival and a randomized prospective trial showed that adding a peptide vaccine to high-dose IL-2 significantly prolonged progression-free survival (PFS) when compared to IL-2 alone.[83;5] Thus, after several decades of development and optimization, there is now evidence that cancer vaccines may improve clinical outcomes, in particular in combination with other therapy.

There is a need to improve defined antigens for melanoma vaccines. Peptide vaccines for melanoma offer the promise of inducing T cells reactive to well-characterized tumor antigens and also enabling assessment of effectiveness of the vaccinations by monitoring antigen-specific T cell responses. However, clinical experience with peptide vaccines has been mixed. On one hand, we and others have found that selected peptides can induce circulating T cell responses in a majority of patients,[8;9] and that vaccination with a mixture of peptides can be immunogenic in up to 100% of patients.[8] In some trials, circulating immune responses have correlated with clinical outcome[8;10], but this has not been the rule. Also, the magnitude of T cell responses sometimes is substantial, with 1-5% of circulating CD8 T cells reactive to a single antigen [4;11;12]; however, responses in most patients represent T cell percentages that are 1-2 orders of magnitude lower, which may or may not be adequate for clinical benefit. The T cell responses to vaccines may be very durable, for months or years, but are at least as likely to be transient, sometimes declining even while still receiving vaccines.[13] Clinically, there have been durable clinical responses in some patients receiving melanoma vaccines, suggesting that there is clinical activity.[14] However, the overall clinical response rates are only about 3-5%.[15] Thus, vaccines are not optimized, and both the antigens and the adjuvants may be improved.

Long peptides. Recent work with longer (30-mer) peptides that encompass short minimal epitope peptides suggests that these longer peptides may be more effective immunogens than the minimal peptides. The extra length contributes to a tertiary structure that may protect from peptidases, and they are too long to be presented directly on MHC; so intracellular processing is required. Importantly, this further ensures that the peptides are presented just on professional APC. A vaccine using long peptides for squamous vulvar neoplasia has been associated with high rates of clinical regressions.[1] This proposal evaluates long peptides as immunogens for melanoma vaccines. It is for a first-in-human evaluation of safety and immunogenicity of 7 long peptides for melanoma, which incorporate well-characterized minimal epitopes restricted by HLA-A1, A2, and A3 and HLA-B35/51.

Adjuvants for cancer vaccines. Some data have challenged the effectiveness of the current formulation of incomplete Freund's adjuvant (IFA) with a peptide vaccine, especially for induction of Th1/Tc1 T cell responses,[16] while data from our own experience supports its adjuvanticity[17]. However, data from our own experience, and from our collaborators also show that vaccines using short peptides in IFA induce chronic inflammation at the site of vaccination that attracts and retains antigen-specific T cells at the vaccine site and may cause T cell dysfunction [18;19]. Thus, there is a need to optimize adjuvants; there also is reason to expect that IFA will function differently with long than with short peptides, as the epitopes derived from processing the long peptides will be presented in the draining nodes, rather at the sites of vaccination.

Toll-like receptor (TLR) agonists offer the potential to improve the magnitude and persistence of antitumor T cell responses [4]; however, most trials of TLR agonists have been limited to use of one TLR agonist formulation, and have not defined the molecular and cellular effects at the vaccine site microenvironment (VSME). In the present study, we propose to test each of 2 TLR agonists in parallel and in combination, with or without IFA, and to evaluate the added value over IFA alone. These include agonists for TLR3, TLR7, and TLR8, to be administered locally at the site of vaccination.

Combination immune therapy: This proposal will enable selection of an effective immunization strategy for combination with other effective systemic immune therapies in future studies. Vaccination with a single HLA-A2 restricted peptide in IFA improved response rate and progression-free survival when combined with HD IL2 in a randomized prospective trial for Stage IV melanoma[5]. Progression-free survival was the primary endpoint for that study. Despite the fact that the study was not powered to detect differences in survival, the vaccine-treated patients had a strong trend to improvement in overall survival (p = 0.06)[5]. However, vaccination with HLA-A2 peptides plus IFA alone in another study had no impact on clinical response.[20] The trial that produced the encouraging positive finding in combination with HD IL2 needs to repeated both for confirmation of benefit and to evaluate impact on survival in an adequately sized study. Recent concerns with the inadequacy of IFA alone as an adjuvant have prevented investigators from moving to a confirmatory trial until a more optimal adjuvant is available.

Other preclinical studies show that adding a vaccine to adoptive T cell therapy increases its therapeutic efficacy.[21-24] Also, vaccination can synergize with PD1/PDL1 blockade to increase immune responses and tumor control.[25] Thus, there is evidence to support the value of combining vaccines plus any of several effective immune therapies. However, vaccines are not yet optimized. The present application will test a vaccine comprised of multiple long peptides and multiple adjuvants. The use of peptides restricted by HLA-A1, A2, A3, and B alleles has a significant advantage for speeding accrual for a future definitive trial by almost doubling potential eligibility over HLA-A2 restriction alone (more than 80% vs. 45% HLA appropriate).

Vaccine site selection: Preliminary data from analysis of several clinical trials of peptide vaccines at the University of Virginia suggests that patients who have been vaccinated at the same skin site each week (for at least 3 vaccines, same site vaccination) have had stronger T cell responses than those with vaccines administered to a different skin site with each vaccine (rotating vaccine sites). However, those data were based on retrospective assessments among trials with varied vaccine adjuvants and over extending time periods. Also, the differences were not definitive enough to be assured that site makes a difference in outcome, We hypothesize that same site vaccination with LPV7 will be safe and will result in stronger $CD4^+$ and $CD8^+$ T cell responses to those peptides. Preliminary data from Part 2 will assess whether there may be a basis for exploring this question in a definitive randomized trial, and will provide a basis for powering such a study.

1.2 Vaccine Design and Surgical Excision

1.2.1 Antigen-Specific Immune Responses Induced by Peptide Vaccines

We have substantial experience in studies with multipeptide vaccines, and with high antigen-specific immune response rates in the peripheral blood and in vaccine-draining nodes.[8;9;12] These provide a strong foundation for evaluation of the multipeptide vaccines proposed in this application, and for the immunologic analyses.

1.2.2 Melanoma Peptides Restricted by MHC Class I Molecules and Incorporated in the prior vaccine trials, that are selected for use in the LPV7 mixture

Antigens representing the majority of melanoma-associated antigens identified thus far can be divided into two groups. The first group consists of melanocytic differentiation proteins (MDP); such antigens are only expressed on cells of the melanocytic lineage and include MART-1/MelanA, gp100/Pmel17, tyrosinase, TRP-1/gp75 and TRP-2 proteins. The second group consists of cancer-testis antigens (CTA) such as MAGE proteins, LAGE-1, and NY-ESO-1. These proteins are encoded by genes expressed in several tumors of different

histologic types, but not in normal tissues, other than testis and placenta. Peptides derived from these proteins are recognized by CTL in the context of various HLA alleles.

Melanocytic differentiation proteins: Gp100 is an enzyme involved in melanin synthesis. Multiple epitopes restricted by Class I MHC molecules have been identified from this protein, including peptides restricted by HLA-A2, and HLA-A3 [26-28], and we chose to incorporate in the present proposal long peptides that represent the following short epitopes: $gp100_{209-217-2M}$ IMDQVPFSV (substitution of M for T at position 210; HLA-A2), and ALLAVGATK ($HLA-A3_{17-25}$). The parent $gp100_{209-217}$ epitope (ITDQVPFSV) has been modified to incorporate a methionine residue at position 210 of the peptide (IMDQVPFSV). This modification increases the binding affinity of the IMDQVPFSV peptide for HLA-A2 and increases its immunogenicity *in vivo* when compared to the native peptide[29;30]. Another gp100 derived epitope, ALLAVGATK, is also naturally processed and presented by melanoma cells, and holds promise for induction of HLA-A3 restricted responses *in vivo* [28]. $CD8^+$ T cell responses to the ALLAVGATK peptide have been detected in multiple prior melanoma vaccine trials [12;31-34]. T-cell lines cultured from lymphocytes infiltrating human melanomas, when administered adoptively to participants with metastatic melanoma, have induced partial or complete remissions in a large subset of participants. Clinical response to this therapy has been reported to be increased when the T-cells used for therapy recognize peptides derived from Pmel-17/gp100 [35]. Thus, there is substantial evidence to support the use of the gp100-derived peptides in the melanoma vaccines.

Tyrosinase is an enzyme involved with melanin synthesis. Epitopes derived from tyrosinase have been identified for HLA-A1, HLA-A2, and other class I alleles [26]. In the proposed study, we are including long peptides incorporating one epitope (DAEKSDICTDEY) restricted by HLA-A1 and one epitope (YMDGTMSQV) restricted by HLA-A2. The parent epitope of $tyrosinase_{240-251}$, DAEKCDICTDEY, has been modified to incorporate a serine residue at position 244 of the peptide (DAEKSDICTDEY). This modification prevents disulfide bond formation within the peptide, but does not interfere with HLA-A1 binding or with T cell recognition [36]. In phase II clinical trials conducted by the UVA HITC (UVA-Mel31, Mel36, Mel39, Mel43, and Mel44), immunological responses against the DAEKSDICTDEY peptide were detected as the dominant peptide target for participants who expressed HLA-A1 and who were immunized with a synthetic peptide mixture containing the DAEKSDICTDEY peptide[12;31-34]. CTL cultured from a vaccine draining node (sentinel immunized node, SIN) after vaccination with DAEKSDICTDEY were capable of lysing $HLA-A1^+$ tumor cells naturally expressing tyrosinase[37]. The naturally occurring HLA-A2 restricted epitope from tyrosinase, $tyrosinase_{369-377}$ YMDGTMSQV, contains a post-translational modification of an asparagine to aspartic acid at residue 371 [38]. In phase II clinical trials conducted by the UVA HITC, immunological responses to the YMDGTMSQV peptide have been detected frequently in stage III and IV melanoma participants who expressed HLA-A2, and were immunized with a synthetic peptide mixture containing this peptide [12;31-34].

MAGE-A1 and MAGE-A10 are members of the MAGE gene family and are expressed in the testes, as well as in several different tumor types such as melanoma, breast, prostate, esophagus, colon, and lung [26]. One epitope derived from MAGE-A1 (SLFRAVITK) is included in the proposed study. The $MAGE-A1_{96-104}$ epitope (SLFRAVITK) was identified by Chaux et al. [39], and was first tested in humans in UVA-Mel39, in which immunogenicity and safety have been demonstrated [33]. It is a highly immunogenic peptide in the Mel39, Mel43, and Mel44 trials [8;12;40].

The MAGE-A10$_{254-262}$ epitope (GLYDGMEHL) was identified by Huang et al., and was shown to be naturally processed and presented by HLA-A2^{+}/MAGE-A10^{+} melanoma cell lines.[41] In a report by Valmori et al., CTL responses to this epitope were readily detected in two-thirds of participants with melanoma whose tumors tested positive for the expression of MAGE-A10.[42] However, the ability of MAGE-A10 synthetic peptide to stimulate immunologic responses in humans had not been tested until UVA-Mel39, in which immunogenicity and safety have been demonstrated and this was found to be very immunogenic[33]. Follow up studies further support immunogenicity of this MAGE-A10 peptide[12;34].

The cancer testis antigen NY-ESO-1 has been extensively studied in human clinical trials, including peptide, protein, and DNA[2;43-46]. Recently, a set of 4 overlapping long (30-mer) peptides have been tested for safety and immunogenicity in patients with NY-ESO-1+ ovarian cancer, using either IFA alone, or IFA plus polyICLC (1.4 mg) [2]. That study incorporated the NY-ESO-1 peptide to be used in the current trial (NY-ESO-1$_{79-108}$; CD4+ and CD8+ T cell responses were induced to a mixture of peptides covering the NY-ESO-1 sequence over residues 79-130, in a majority of evaluated patients.

For the present study, the 7 peptides have been selected for use as long peptides. Their sequences are listed in Table 2 in the protocol précis. The reason for selecting six of them is that their minimal epitopes are among the most immunogenic in the set of 12 peptides included in our prior trials [12;33;34]: DAEKSDICTDEY, YMDGTMSQV, IMDQVPFSV, GLYDGMEHL, ALLAVGATK, SLFRAVITK, and they have been administered safely [12;33;34]. We had also selected the peptide EVDPIGHLY from those prior trials, but the long peptide encompassing EVDPGIHLY could not be synthesized with adequate purity; the remaining 6 peptides were selected for use.

The NY-ESO-1$_{79-108}$ peptide was included based on the encouraging immunogenicity and safety findings referred to above with four NY-ESO-1 peptides[2]. Among the four peptides used in that trial, this one was available in adequate quantity as GMP-grade preparation.

Selection and synthesis of long peptides encompassing the short peptide antigens: The NY-ESO-1$_{79-108}$ peptide incorporates a defined nonamer epitope restricted by HLA-B35 and HLA-B51; the sequence was one of 4 previously used as the 30-mer peptide in a phase I trial. For the present study, the 7 long peptides selected for use have been designed to incorporate the minimal epitope sequences above, plus extension at the N and C terminal ends, with attention to avoid N-terminal glutamic acid or glutamine residues, to minimize cysteine residues, and to maximize incorporation of polar residues to aid in aqueous solubility. Their sequences are listed in Table 2 in the protocol précis.

1.2.3 Montanide ISA-51 as a Vaccine Adjuvant.

Montanide adjuvant has been effective at inducing immune responses against murine viral antigens when administered with a synthetic peptide epitope [47;48]. Montanide ISA-51 has also been used with peptides plus GM-CSF-in-adjuvant vaccine useful for enhancing both cellular and humoral immunity.[49] The product consists of a mineral oil base comparable to incomplete Freund's adjuvant. However, the Arlacel A emulsifying agent of incomplete Freund's, which has caused reactions in the past, has been replaced with a purified mannoside monooleate called "montanide", which appears to be safer. The UVA HITC has sponsored studies where peptide-based vaccines in Montanide ISA-51 have been safely administered to more than 500 participants. Immunological responses against the

immunizing peptides have been detected in most participants. Our recent experience with the current formulation of this adjuvant is published.[17;50]

1.2.4 PolyICLC (Hiltonol, Oncovir Inc.), TLR3 agonist.

Polyinosinic-Polycytidylic acid (PolyIC) is a double-stranded RNA (dsRNA) that acts as a TLR3 agonist. However, its short half-life limits its usefulness. To increase half-life and its practical use in clinical settings, it has been stabilized with polylysine and carboxymethylcellulose as polyICLC. Like polyIC, polyICLC is a TLR3 agonist. TLR3 is expressed in the early endosome of myeloid DC; thus polyICLC preferentially activates myeloid dendritic cells, favoring a Th1 cytotoxic T-cell response.[51;52] PolyICLC also activates NK cells and induces cytolytic potential.[52] It has been administered in the emulsion with Montanide ISA-51 plus long peptides, with increased immunogenicity over Montanide ISA-51 plus peptides alone; the combination was safe, with some significant local reactions but no DLTs [2].

Source: Hiltonol is provided by the Ludwig Institute for Cancer Research and its Cancer Vaccine Consortium at no charge; as they have purchased a lot of it from Dr. Andres Salazar from Oncovir, Inc. (Washington, D.C.; letter provided). It is provided as a clinical grade reagent for experimental use in single-use vials containing 1 mL of a 2 mg/mL solution.

Dosing and preparation**:** It has been used safely in cancer patients, with intravenous doses up to 300 mcg/kg.[53] We will administer 1 mg (0.5 mL) per vaccine, as used in other trials (e.g.: NCT01008527).

1.2.5 Resiquimod: TLR7 and 8 agonist.

This agent is an agonist for both TLR7 and TLR8. TLR8 activation may reduce regulatory T cells.[54] It has been safe when administered topically as a 0.05% gel once a week; more frequent administration led to dose-limiting local skin toxicity.[55] Thus, weekly dosing is selected for this trial. Other human experience with resiquimod is that oral dosing at 0.01 mg/kg (700 mcg/70kg) caused increases in serum IFN-alpha with associated side effects, but it was well tolerated.[56] It has been used (NCT00821652) safely at 500 mg 0.2% resiquimod gel per application up to 3x/week after each vaccine, with the dose spread over a 25 cm2 area each time.

Source: Resiquimod is provided by 3M Pharmaceuticals, St. Paul, MN. It is not yet FDA approved, but is available as a clinical grade reagent for topical use. We have approval from Mark Tomai at 3M to provide it for clinical use as a vaccine adjuvant.

Dosing and preparation**:** Multi-dose tubes contain approximately 3 grams of 0.2% resiquimod gel. The dose of resiquimod in 500 mg of 0.2% resiquimod gel is 1000 mcg. Immediately after vaccine injection, measure out approximately 500 mg of 0.2% resiquimod on dosing paper and apply resiquimod gel with a gloved finger over a 25 cm^2 area of the skin at the vaccine site. Using this dosing method, the dose range of resiquimod will be approximately 400-600 mg. Rub the gel into the skin for 1 minute until it vanishes. Following application, the site may be covered with an occlusive dressing, such as Tegaderm, for about 8 hours. Participants are encouraged to wash the area at 8 hours after application.The tubes may be retained in the investigational pharmacy between uses.

1.2.6 Integration of Peptide-Based Vaccines in the Adjuvant Setting

High-dose interferon (IFN-alpha therapy) is approved by the FDA for use in patients with resected stage IIB or III melanoma treated in the adjuvant setting. Approval was based largely on survival improvement observed following a one-year regimen with IFN as described in ECOG 1684.[57] However, survival improvement is modest and data from a subsequent trial, ECOG 1690, failed to reproduce differences in survival.[58] Data from a third trial, ECOG 1694, reveal a statistically significant, but still modest, survival improvement with IFN therapy compared to a ganglioside vaccine.[59] Thus, any patient with resected stage IIB or III melanoma should be offered IFN therapy, which is routine practice at UVA and MDACC. However, the toxicity of high-dose IFN therapy is substantial. Approximately 25-30% of patients withdraw from therapy because of toxicity, and approximately 60% of patients require dose reductions during treatment.[57]

Ipilimamb 10mg/kg is approved by the FDA for use in the adjuvant setting for patients who have undergone complete resection of cutaneous melanoma with pathological involvement of regional lymph nodes greater than 1 mm. Approval was based largely on results from the the Phase 3 EORTC 18071/CA184029 trial.[84] The ipilimumab 10mg/kg treatment yielded statistically significant prolonged distant metastasis-free survival, with five-year distant metastasis-free survival rate 9% higher than the placebo. However, the toxicty of ipilimumab 10mg/kg is is substantial, with approximately 50% of patients who started ipilimumab withdrawing as a result of ipilimumab related toxicities.

The experience at UVA is that most patients who are candidates for therapy refuse IFN because of concerns with toxicity, despite being informed IFN therapy is one of the few FDA approved adjuvant therapies for melanoma and that therapy may provide a survival advantage. In addition, many patients are not eligible for the current approved dose of ipilimumab (10 mg/kg) or decline treatment with ipilimumab because of the potential for serious immune-related toxicities. Recent data from the E1609 study presented at ASCO showed that there was no difference in disease-free-survival between ipilimumab 3 mg/kg and ipilimumab 10 mg/kg (E1609); however, ipilimumab 10 mg/kg was associated with greater toxicity.[85] Thus, future approved treatment options may include ipilimumab administered at the lower dose of 3 mg/kg, although even the lower dose carries risk of death. The same may be true for other checkpoint inhibitors that are currently being explored in the adjuvant setting (e.g. PD-1 blockade).

1.2.7 Toxicology

There is no reason to expect direct toxicity of the melanoma peptides; they are not directly cytotoxic *in vitro* and are not expected to have functional implications. On the other hand, because some of these peptides are identical or similar to a portion of a normal protein, risks of autoimmunity in humans are reasonable to evaluate. Unfortunately, there is no murine system adequately modeling the human immune response to these peptides. The most meaningful evaluation of this peptide vaccine mixture is in patients with melanoma. This trial is for participants with resected stage IIB/C, III, or IV NED melanoma. These individuals face a high risk (> 40%) of death from melanoma, and the anticipated risk of short-term or long-term toxicity of this vaccine preparation is minimal, while the vaccine may delay or decrease the risk of morbidity and mortality due to melanoma in these participants. The potential implications of autoimmunity against cells of melanocytic lineage are illustrated by reported cases of vitiligo occurring coincident with regressions of melanoma.[60] Most of these are limited, often occurring in skin surrounding the regressing melanoma, but

occasionally occurring systemically. While pathogenesis of this phenomenon can only be hypothesized, it is reasonable to consider this a worst-case scenario.

The loss of skin and hair pigment can be striking in cases of vitiligo, but is not a cause of morbidity or mortality. Of greater potential concern is the theoretical risk of damage to the retinal pigment epithelium; however, visual loss has not been reported either as a complication of successful immunotherapy with melanoma-reactive CTL or spontaneous vitiligo. However, some ocular toxicity has been reported that resolved with steroid treatment.[61]

Depigmentation of the retinal pigment epithelium has been observed in a small number of patients vaccinated with dendritic cells pulsed with MDP-derived peptides; however, this change was asymptomatic and was not associated with loss of visual acuity (personal communication – Frank Haluska). A careful study of the retinal pigment epithelium using monobenzyl ether of hydroquinone to induce pigment cell destruction on a biochemical basis suggests the safety of pigment cell destruction and supports immunotherapy directed against MDP as a strategy for melanoma therapy (personal communication, JM Kirkwood).

Thus far, 10-15% of participants receiving peptide-based vaccines in the studies at UVA were diagnosed with definite vitiligo. In all cases, the vitiligo was asymptomatic. No major visual/ocular toxicity was reported for participants on these prior trials.

<u>Toxicities Previously Reported for Participants Receiving the 12-MP vaccine administered in Montanide ISA-51 adjuvant</u>

Participants have been vaccinated with a mixture of 12 short melanoma peptide mixture, plus tetanus peptide, in Montanide ISA-51 adjuvant (MELITAC 12.1) in 62 participants on the UVA-Mel43 trial, to about 80 participants in the UVA-Mel44 trial, and to about 40 participants on the UVA-Mel48 trial. Toxicity data for the first 102 participants on the Mel43 and Mel44 trials were graded using the NCI Common Terminology Criteria for Adverse Events v3.0 and are listed regardless of attribution. Maximum Grade 2-4 toxicities experienced by at least 10% of participants are listed in Table 3:

Table 3. Toxicities related to administration of MELITAC 12.1 melanoma vaccine administered with Montanide ISA-51 (IFA) in 102 patients on UVA-Mel43 and UVA-Mel44 clinical trials

Toxicity	Grade 2	Grade 3
Fatigue (lethargy, malaise, asthenia)	17%	3%
Injection site reaction	87%	2%
Ulceration	7%	3%

The grading of injection site reactions was modified from CTC v2.0 to CTCAE v3.0, and now to v4.03. CTC v2.0 did not have an ulceration Adverse Event Term, therefore participants with ulceration in conjunction with an injection site reaction were graded as experiencing a Grade 3 injection site reaction (ulceration or necrosis that is severe; operative intervention indicated). In prior UVA HITC studies, an injection site reaction with ulceration ≤ 2 cm in maximal diameter was an expected adverse event in a subset of participants. However, an injection site reaction that included ulceration >2 cm in diameter, or an injection site reaction requiring debridement, narcotic analgesics for pain, or surgery was not an expected toxicity and was considered a dose-limiting toxicity (DLT) and was graded as a Grade 3 injection site reaction. UVA-Mel43 used the CTC v2.0 grading system whereas UVA-Mel44 used the CTCAE v3.0 grading system. In CTCAE v3.0, ulceration is a separate Adverse Event Term;

therefore, ulceration and injection site reactions can be graded separately. For this study, participants who experience induration at their injection site, both injection site reaction and induration will be graded and reported. The data in Table 3 have been corrected to be consistent with CTCAE v3.0. The criteria for each of these toxicity categories (fatigue, injection site reaction, and ulceration) have changed from v3.0 to v4.03, but are very similar. Thus, they are probably comparable, but the exact details for assessing them in v4.03 require information that was not collected at the time those studies were performed. In the proposed study, we will grade based on v4.03, and we do not expect Grade 3 injection site reactions; ulceration and injection site reaction toxicities will be expected at Grade 2.

1.2.8 Patient acceptance of biopsies of skin at the Vaccine Site

This study will include three punch biopsies 4mm in diameter of the vaccine site, on two dates. We have previously performed much larger excisions of skin and subcutaneous tissue approximately 2 x 4-6 cm in size.[50] There were questions, prior to that study, of whether that size excision would be too large to expect participants to tolerate or to accept it. As a surgeon, the Principal Investigator (PI) routinely performs excisions 2-4 cm wide (and about 6-12 cm long) in patients when they have wide excisions of melanoma, and these can reliably be closed primarily, without a skin graft, when taken from the upper thigh and upper arm. Thus, excisions of a 2x4 cm skin section (regardless of depth) can be closed primarily with minimal morbidity on those locations where the vaccines would be given.

Also, we surveyed a convenience sample of 52 of our patients during clinic visits for their willingness (on a scale of 1 to 9) to participate in a clinical trial of a melanoma vaccine with or without a skin biopsy. Forty-three (43) of the 52 patients (83%), answered some of the questions, and 37 (71%) answered all questions. Findings are summarized in Table 4 below and have been published[62]. Willingness to enter such a trial was diminished by any biopsy requirement, but interest in accrual remained high even with a biopsy. Interestingly the size of the biopsy had a much smaller impact than the fact that any biopsy was required. In prior trials, we have included excision of a vaccine-draining lymph node (sentinel immunized node), which requires a 3 cm incision, and we have done over 150 of such biopsies on vaccine patients.[31-33;37] Based on that questionnaire, patients were expected to be about 70-80% as likely to enroll on a trial with a 6 cm excision vs. a 3 cm excision as without a biopsy. The actual accrual matched closely the predicted accrual.[62]

The present proposal is to remove much less skin that was done in that prior protocol (Mel48). Thus, we anticipate rapid accrual and high patient acceptance. We typically have accrued 40-60 patients per year on trials with sentinel immunized node biopsies; so if we accrue at even half that rate (20-30 per year), we expect to meet accrual goals.

Table 4. Findings from vaccine biopsy questionnaire (n = 91)

Willingness to enter vaccine trial with or without skin excision	N	Likely (5) to highly likely (9)	Moderately likely (7) to highly likely (9)	Highly likely (score 9)
No skin excision	43	95 %	69 %	54 %
3 cm scar	37	74 %	47 %	28 %

1.2.9 Regulatory Issues

The peptides for this trial have been synthesized under GMP conditions by a commercial provider. Prior experience has been with peptides made by Polypeptide Group, San Diego, California. They have been vialed under GLP conditions in our HITC lab clean room, which

has limited access, an entry room for gowning and gloving, and positive pressure ventilation and laminar flow hoods. Lot release testing of the final vialed peptides will be completed in accord with CFR guidelines. Stability testing will be conducted regularly, in accord with the Chemistry and Manufacturing section of the IND application.

Montanide ISA-51 is produced by Seppic, Inc. (Fairfield, NJ and Paris, France). A master drug file for Montanide ISA-51 is filed with the FDA. This agent has been used in hundreds of patients in our own experience and in thousands of patients worldwide.

This study will be approved by the Institutional Review Board (IRB) of the University of Virginia and by the IRBs of M.D. Anderson Cancer Center. This protocol will be submitted to the FDA as an IND application. Records of all study review and approval documents will be kept on file by the Principal Investigator, and are subject to FDA inspection during or after completion of the study. The Institutional Review Board will receive notification of study closure within three months of study termination or completion.

This study will be conducted in compliance with Good Clinical Practices and regulations.

1.2.10 Same site vaccination (Part 2)

The clinical trials group at the University of Virginia has extensive experience in several hundred patients with administering peptide vaccines in IFA, with or without other immune modulators, at the same skin site for each vaccine. This has been well-tolerated [34;77-99]. Later trials with rotating vaccine sites were performed for several reasons, but with the expectation that vaccine site reactions would be less prominent; however, overall vaccine site reactions have been similar with both approaches (Table 5):

Table 5: Vaccine Site Reactions

	Clinical trial	N	CTCAE	Grade 1	Grade 2	Grade 3
Injection site reaction	Mel44	170	V3	17%	75%	4%
	Mel58	51	V4	35%	61%	0%
Ulceration	Mel44	170	V3	1%	19%	6%
	Mel58	51	V4	0%	4%	2%

Note: data are from the published data from the Mel44 trial [2] and the 2017 IND report for the Mel58 trial.

The LPV7 vaccine has been well-tolerated in the patients on Part 1. Same site vaccination will be assessed for safety/tolerability and immunogenicity in a pilot cohort in Part 2.

2.0 STUDY OBJECTIVES

2.1 Part 1 (completed)

(1) To assess safety of vaccination with a mixture of long peptides for patients with high-risk melanoma, in each of 7 adjuvant preparations, including TLR agonists and/or incomplete Freund's adjuvant (IFA)

(2) To estimate the immunogenicity of 7 long peptides in each of 7 adjuvant preparations

Exploratory

(3) To evaluate the molecular and cellular changes at the vaccine site and systemically

2.2 Part 2

(4) To assess safety of vaccination of the optimal combination identified in Part 1 when administered in the same skin site for all 6 vaccines (same site vaccination).

(5) To estimate the immunogenicity of the optimal combination identified in Part 1 when administered in the same skin site for all 6 vaccines (same site vaccination).

3.0 STUDY OUTLINE

3.1 Type of Study

This is an open-label, randomized, phase I/II study, using an adaptive design, to test the safety and immunogenicity of a novel melanoma vaccination approach using long peptides plus TLR agonists, along with characterization of cellular and molecular events at the cutaneous site of immunization.

Regimen:

Part 1: Patients will be registered on this phase I/II trial and will be vaccinated with LPV7 at 300 mcg peptide/dose, and tetanus peptide at 200 mcg/dose, every week x 3 then every 3 weeks x 3. The adjuvants are described, and will be administered to patients in 7 study groups as shown in Tables 1 and 5. This incorporates an adaptive design with sequential initiation of accrual to study Zones with increasing numbers of adjuvants (Arms A-C, then D-F, then G) and a 3 week interval between subjects 1 and 2 of each arm of the study. The design allows, during that 3 week interval, randomization to arms that remain open.

Part 2: Patients will be registered on this phase I/II trial and will be vaccinated with LPV7 at 300 mcg peptide/dose every week x 3 then every 3 weeks x 3. For part 2, the vaccine regimen is selected based on available data and the study decision rules from Part 1 which was determined to be Arm E. For Part 2 accrual will be to Study Arm E2. However, a modification is made to exclude the tetanus helper peptide, due to (a) lack of availability of that peptide component, (b) the finding that, in part 1, LPV7 peptides do induce helper T cell responses themselves (unpublished data), and prior data that adding the tetanus helper peptide did not add significantly to immunologic or clinical outcome of a peptide vaccine designed to induce CD8 T cells[1].

4.0 PARTICIPANT SELECTION

All questions regarding eligibility should be directed to the Human Immune Therapy Center (HITC) at the University of Virginia.

4.1 Inclusion Criteria

4.1.1 Histologically or cytologically proven Stage IIB-IV melanoma (at diagnosis or at the time of recurrence) rendered clinically free of disease by surgery, other therapy, or spontaneous remission within 6 months prior to registration; patients with treated brain metastases may be eligible if they meet the criteria in 4.1.4. Small radiologic or clinical findings of an indeterminate nature will not be a basis for exclusion, and brain metastases treated in accord with section 4.1.4 will not be a basis for exclusion.

Staging of cutaneous melanoma will be based on the 7th edition AJCC staging system (Appendix 2)[63].

Staging of mucosal melanomas will be based on the following system modified from the cutaneous melanoma staging system: 2.01- 4mm primary with ulceration or > 4mm primary= stage IIB, lymph node metastases = stage III, distant metastases = stage IV.

4.1.2 Patients may have had multiple primary melanomas.

4.1.3 Patients may have had or may have a metastasis from a cutaneous primary site, mucosal primary site, or unknown primary site.

4.1.4 Patients with brain metastases may be eligible if all of the following are true:

- The total number of brain metastases ever is less than or equal to 3.
- The brain metastases have been completely removed by surgery or have been treated completely by stereotactic radiotherapy. Stereotactic radiotherapy, such as gamma knife, can be used up to 1 week prior to study entry. In the absence of concerning clinical findings, repeat scans are not required after stereotactic radiotherapy if the patient enrolls within 8 weeks of completing the stereotactic therapy.
- There has been no evident growth of any brain metastasis since treatment.
- No treated brain metastasis is greater than 2 cm in diameter at the time of protocol entry.

4.1.5 Patients must have at least one axillary and/or inguinal lymph node basin that is intact (no prior excisional biopsy of a node or complete lymph node dissection).

4.1.6 All patients must have:

- ECOG performance status of 0 or 1.
- Ability and willingness to give informed consent.

4.1.7 Laboratory parameters as follows:

1) HLA-A1, A2, A3, B35, or B51.
2) ANC > 1000/mm^3, and Platelets > 100,000/mm^3 and Hgb > 9 g/dL
3) AST and ALT up to 2.5 x upper limits of normal (ULN). Patients known to have Gilbert's disease may be eligible with AST and ALT up to 5 x ULN.
4) Bilirubin up to 2.5 x ULN
5) Alkaline phosphatase up to 2.5 x ULN.
6) Creatinine up to 1.5 x ULN
7) Hgb-A1C level of ≤ 7.5%

4.1.8 Patients must be 18 years or older at study entry.

4.2 Exclusion Criteria

4.2.1 Patients who have had brain metastases unless they meet the criteria outlined in section 4.1.4.

4.2.2 Patients with melanoma arising from uveal/ocular primary sites

4.2.3 Patients who are currently receiving systemic cytotoxic chemotherapy, radiation, targeted molecular therapy (e.g. vemurafenib, other inhibitor of mutant BRAF, MEK, or cKit), or other experimental therapy, or who have received this therapy within the preceding 4

weeks (except as specified in section 4.2.4). Gamma knife or stereotactic radiosurgery may be administered within the prior 4 weeks, but must not be administered less than one week prior to study registration. Patients who are currently receiving nitrosoureas or who have received this therapy within the preceding 6 weeks are excluded.

4.2.4 Patients who are receiving or have been treated with antibody to CTLA4 (e.g. ipilimumab), PD-1, PD-L1, CD137, or CD27 within the prior 12 months. Any patient who has had one or more of these therapies greater than 12 months prior and is clinically free of disease and totally recovered from toxicities related to those therapies can be eligible.

4.2.5 Patients with known or suspected allergies to any component of the vaccine.

4.2.6 HIV positivity or evidence of active Hepatitis C virus.

4.2.7 Patients receiving the following medications at study entry or within the preceding 4 weeks are excluded:

1) Agents with putative immunomodulating activity (with the exception of non-steroidal anti-inflammatory agents and topical steroids (see section 4.2.7 (3)).
2) Allergy desensitization injections.
3) Systemic corticosteroids, administered parenterally or orally. Inhaled steroids (e.g. Advair®, Flovent®, Azmacort®) are not permitted. Topical corticosteroids are acceptable, including steroids with very low solubility administered nasally for local effects only (e.g. Nasonex®).
4) Any growth factors (e.g. GM-CSF, G-CSF, erythropoietin).
5) Interferon therapy.
6) Interleukin-2 or other interleukins.
7) For immune modulating agents referred to in 4.2.3 and 4.2.4, it may be necessary for more than 4 weeks to have elapsed since completion of that therapy (as specified in those sections).

4.2.8 Prior melanoma vaccinations may be an exclusion criterion in the following circumstances:

1) Patients who have received, within the prior 5 years, melanoma vaccines or other vaccines containing an incomplete Freund's adjuvant (IFA; such as Montanide ISA-51).
2) Patients who have received any melanoma vaccine within the past 12 months.
3) Patients who have been vaccinated in the past with any of the melanoma peptides in the LPV7 vaccine included in this study.

4.2.9 Other investigational drugs or investigational therapy if the patient is currently taking those drugs/therapy, or if they have received the drugs/therapy within 1 month.

4.2.10 Pregnancy or the possibility of becoming pregnant during vaccine administration. Female patients of child-bearing potential must have a negative pregnancy test (urinary or serum beta-HCG) prior to administration of the first vaccine dose. Males and females must agree, in the consent form, to use effective birth control methods during the course of

vaccination. Women must also not be breast feeding. This is consistent with existing standards of practice for vaccine and chemotherapy protocols.

4.2.11 Participants must not have had prior autoimmune disorders requiring cytotoxic or immunosuppressive therapy, or autoimmune disorders with visceral involvement. Participants with an active autoimmune disorder requiring these therapies are also excluded. The following will not be exclusionary:

- The presence of laboratory evidence of autoimmune disease (e.g. positive ANA titer) without symptoms
- Clinical evidence of vitiligo
- Hypothyroidism of any etiology on stable thyroid hormone replacement therapy.
- Other forms of depigmenting illness
- Mild arthritis requiring NSAID medications

4.2.12 Patients in whom there is a medical contraindication or potential problem in complying with the requirements of the protocol, in the opinion of the investigator.

4.2.13 Patients classified according to the New York Heart Association classification as having Class III or IV heart disease (Appendix 4).

4.2.14 Patients with a body weight < 110 lbs (50 kg) because of the amount and frequency with which blood will be drawn, and because of the skin biopsies required.

4.3 Demographics of Participant Population

4.3.1 Age: 18 years or older.

4.3.2 Sex: Both male and female participants are eligible.

4.3.3 Race: All races and ethnic backgrounds are eligible.

4.4 Registration, Randomization, and Management of Participants

4.4.1 Participant Registration

All participants must sign the consent form prior to determination of eligibility for this study. Participants eligible for interferon therapy will complete the interferon education packet and review their answers with the study team and convince the study team that they answered the questions accurately or understand the correct answers. Registration information, the eligibility checklist with supporting documentation, and the on-study case report form for potential participants at institutions other than UVA must be received by the UVA HITC prior to registration and randomization. All participants who meet the inclusion/exclusion criteria may be registered to the study. Registration will occur following verification of eligibility by the treating physician or institutional PI.

4.4.2 Randomization

Randomization will be discussed with participants during the process of informed consent, and informed consent must be documented prior to randomization. Randomization will occur after registration and no sooner than 5 days prior to the start of treatment. In part 1, randomization will be based on equal allocation among allowable arms unless a weighted allocation scheme is triggered (section 9.2.1). Randomization will not be stratified by

institution. The randomization codes are generated by the study statisticians and stored in the Cancer Center Clinical Trials Database. The UVA HITC will communicate with the outside site to let them know which study arm a participant has been randomized to. Participants should receive the first scheduled vaccination or biopsy (if applicable) within 2 weeks of registration. Part 2 will not include randomization.

4.4.3 Management of Participants
This study will be conducted on an outpatient basis, with participants scheduled to be evaluated on days 1, 8, 15, 22, 36, 43, 57, 78, and 85, and week 26 (or more often if needed for testing or medical reasons). As needed, there may also be follow-up within 1-3 weeks after biopsies performed on days 8 or 22 for wound evaluation. Participants will also be followed-up at 52 weeks (1 year) and at 2 years with blood draws for studies of immune response durability. During off treatment follow-up, participants will be followed yearly for survival and disease status.

5.0 STUDY MEDICATION FORMULATION, DOSAGE, ACCOUNTABILITY AND ADMINISTRATION

5.1 Study Medication Formulation

5.1.1 Peptide Synthesis and Storage
All peptides are synthesized directly from amino acids, and then are purified by high performance liquid chromatography (HPLC). The identity of the synthetic peptides will be confirmed by verifying their mass and amino acid sequences by mass spectrometry. Details of the synthesis, certificates of analysis, and technical summaries are included in the Chemistry and Manufacturing section of the IND application. Each bulk peptide is supplied as lyophilized powder without excipients and stored at a temperature ≤ -70°C and protected from light.

5.1.2 Preparation of peptide vaccine vials for patient use.
For the 6 peptides prepared at UVA, the bulk peptides are solubilized in aqueous solution, sterile-filtered, and mixed to provide a final concentration of 600 mcg/ml for each of the 6 peptides. The peptide mixture is then aliquoted at 1 ml per vial into sterile borosilicate vials in the HITC clean room. The mixture is slightly cloudy, as the pH of the mixture supports solubility of most of the peptides, but some peptides are partially in suspension at that pH. The 7th peptide, from NY-ESO-1 is provided as single use vials of lyophilized peptide, at 600 mcg/vial, from the Ludwig Institute for Cancer Research, prepared under GMP conditions.

5.1.3 Storage of Vialed Peptides
The vials of peptide are stored by the HITC at a temperature ≤ -70°C and protected from light. On the day of use, they are thawed at room temperature or in a water bath up to 37°C. They should be swirled gently as they are thawed and moved to a refrigerator as soon as they are thawed if not being injected immediately. Once thawed, the vial(s) must be used for preparation of the vaccine within 24 hours.

5.1.4 Lot Testing
Each lot of peptide vaccine is evaluated as required by the FDA to confirm identity, sterility, general safety, purity, lack of peptide aggregation and is tested for endotoxin. In addition,

studies of stability will be performed over time. The details of these tests are outlined in Appendix 5.

5.1.5 Labeling
The labeling of vials will occur as specified below.

Each vial of lyophilized peptide is labeled with the following information:
Short name of the product: “LPV6”
Product number
Proper name of the product: “6 long melanoma peptides”
Name and address of the vialing facility: “UVA-HITC”
Lot number
Date of manufacture (the date of vialing the reconstituted peptides)
Serial number
Quantity of each peptide per vial: 600 mcg/ml
Vial contains no preservative, store at ≤ -70°C
“Caution: New Drug – Limited by US Federal law to investigational use”

5.2 Preparation, Dosage, and Storage of Study Drug

5.2.1 Dosage and Preparation of Peptide Vaccine
Part 1:
The peptides will be prepared by combining the LPV6 mixture of 6 peptides with one NY-ESO-1 long peptide. The long melanoma peptides (LPV6) will be available as single-use vials of 1 ml volume containing 600 mcg/ml of each peptide. The NY-ESO-1 peptide is provided as lyophilized preparation with 600 mcg of peptide in single use vials. The tetanus peptide is vialed as 300 mcg lyophilized peptide per vial. For each vaccine, the full 1 ml of the peptide solution of LPV6 (600 mcg) will be withdrawn and injected into the vial of lyophilized NY-ESO-1 peptide, creating a solution/suspension of all 7 long peptides, each at 600 mcg/ml. Then 0.75 ml of this suspension/solution will be withdrawn and injected into one vial of lyophilized tetanus peptide, to create a suspension/solution of 450 mcg of each of the 7 long peptides, and 300 mcg of tetanus peptide in 0.75 ml. Then, the full 0.75 ml of this solution/suspension (450 mcg of all 7 long peptides, plus 300 mcg tetanus peptide) will be withdrawn into a syringe for injection as specified below for each study arm. Details of making emulsions with IFA (Montanide ISA-51) are provided in the Investigator’s Brochure and Study Manual.

Part 2:
The peptides will be prepared by combining the LPV6 mixture of 6 peptides with one NY-ESO-1 long peptide. The long melanoma peptides (LPV6) will be available as single-use vials of 1 ml volume containing 600 mcg/ml of each peptide. The NY-ESO-1 peptide is provided as lyophilized preparation with 600 mcg of peptide in single use vials. For each vaccine, the full 1 ml of the peptide solution of LPV6 (600 mcg) will be withdrawn and injected into the vial of lyophilized NY-ESO-1 peptide, creating a solution/suspension of all 7 long peptides, each at 600 mcg/ml. Then 0.75 ml of this solution/suspension (450 mcg of all 7 long peptides) will be withdrawn into a syringe for injection as specified below for each study arm. Details of making emulsions with IFA (Montanide ISA-51) are provided in the Investigator’s Brochure and Study Manual.

Study Arm A (IFA): Sterile saline solution 0.75ml will be added to 0.75 ml of LPV7 + tetanus peptide (total 1.5 ml). This will be combined with 1.5 ml of Montanide ISA-51VG to create an emulsion. Two (2) ml will be injected half-intradermally and half-subcutaneously in one skin location.
Study Arm B (polyICLC): PolyICLC (Hiltonol) is provided from Oncovir, Inc. (Washington, D.C.) as a clinical grade reagent for experimental use in single-use vials containing 1 mL of a 2 mg/mL solution. We will administer 1 mg (0.5 ml) per vaccine. PolyICLC (Hiltonol) 0.75 ml will be added to 0.75 ml of LPV7 + tetanus peptide. One (1) ml of this will be injected half-intradermally and half-subcutaneously in one skin location.
Study Arm C (resiquimod): Resiquimod is provided by 3M (Minneapolis, MN) and is packaged in multi-dose tubes (typically 3 g of 0.2% gel). Sterile saline solution 0.75ml will be added to 0.75 ml of LPV7 + tetanus peptide. One (1) ml of this will be injected half-intradermally and half-subcutaneously in one skin location. Immediately after vaccine injection, apply approximately 500 mg of 0.2% resiquimod gel with a gloved finger over a 25 cm^2 area of the skin at the vaccine site. Rub the gel into the skin for 1 minute until it vanishes. Following application, the site may be covered with an occlusive dressing, such as Tegaderm, for about 8 hours. Participants are encouraged to wash the area at 8 hours after application.
Study Arm D (polyICLC + resiquimod): PolyICLC (Hiltonol) 0.75 ml will be added to 0.75 ml of LPV7 + tetanus peptide. One (1) ml of this will be injected half-intradermally and half-subcutaneously in one skin location Immediately after vaccine injection, apply approximately 500 mg of 0.2% resiquimod gel with a gloved finger over a 25 cm^2 area of the skin at the vaccine site. Rub the gel into the skin for 1 minute until it vanishes. Following application, the site may be covered with an occlusive dressing, such as Tegaderm, for about 8 hours. Participants are encouraged to wash the area at 8 hours after application.
Study Arm E (IFA + polyICLC): Part 1: PolyICLC (0.75 ml) will be added to 0.75 ml of LPV7 + tetanus peptide (total 1.5 ml). This will be combined with 1.5 ml of Montanide ISA-51VG to create an emulsion. Two (2) ml will be injected half-intradermally and half-subcutaneously in one skin location.

Study Arm E2 (IFA + polyICLC): Part 2: PolyICLC (0.75 ml) will be added to 0.75 ml of LPV7 (total 1.5 ml). This will be combined with 1.5 ml of Montanide ISA-51VG to create an emulsion. Two (2) ml will be injected half-intradermally and half-subcutaneously in one skin location (as in study arm A).

Study Arm F (IFA + resiquimod): Sterile saline solution 0.75ml will be added to 0.75 ml of LPV7 + tetanus peptide (total 1.5 ml). This will be combined with 1.5 ml of Montanide ISA-51VG to create an emulsion. Two (2) ml will be injected half-intradermally and half-subcutaneously in one skin location (as in study arm A). Immediately after vaccine injection, apply approximately 500 mg of 0.2% resiquimod gel with a gloved finger over a 25 cm^2 area of the skin at the vaccine site. Rub the gel into the skin for 1 minute until it vanishes. Following application, the site may be covered with an occlusive dressing, such as Tegaderm, for about 8 hours. Participants are encouraged to wash the area at 8 hours after application.
Study Arm G (IFA + polyICLC + resiquimod): PolyICLC (0.75 ml) will be added to 0.75 ml of LPV7 + tetanus peptide (total 1.5 ml). This will be combined with 1.5 ml of Montanide ISA-51VG to create an emulsion. Two (2) ml will be injected half-intradermally and half-subcutaneously in one skin location (as in study arm A). Immediately after vaccine and polyICLC injection, apply approximately 500 mg of 0.2% resiquimod gel with a gloved finger over a 25 cm^2 area of the skin at the vaccine site. Rub the gel into the skin for 1

minute until it vanishes. Following application, the site may be covered with an occlusive dressing, such as Tegaderm, for about 8 hours. Participants are encouraged to wash the area at 8 hours after application.

Storage of Prepared Peptide Vaccines
The prepared peptide vaccines will be stored in a plastic syringe and delivered to the clinicians in a plastic bag. This bag with the syringe will be stored at room temperature until the vaccine is administered. Ideally, the vaccine should be administered within 1-2 hours after mixing. If the vaccine is not administered within 4 hours after mixing, it should be discarded.

5.3 Drug Accountability and Distribution
Study drug will be accounted for and distributed to UVA patients and the outside sites using the HITC InvestMed Database. The InvestMed Database is an Access database that interfaces with the Cancer Center Clinical Trials clinical database so that drug may be allocated and dispensed on a per patient basis to UVA patients. For the outside sites, drug is dispensed to the corresponding institution and tracked on a per institution basis. Study drug will be sent to the outside sites by HITC personnel.

5.4 Administration of Vaccines

5.4.1 Designation of Vaccine Sites
Evidence suggests nodes proximal to a tumor site may be relatively immunosuppressed [64]; therefore, the vaccination sites will be distant from sites of known tumor whenever possible. In general, participants will be vaccinated in upper arm or thigh locations. If a participant has received a prior experimental vaccine, vaccines should not be administered at the same site as the prior experimental vaccine, but they may be administered on the same extremity as prior vaccines.

5.4.2 Regimen
Part 1: Each vaccine will be administered at a different site, rotating among available extremities when possible.

Part 2: The same skin location will be used for all vaccines. If the vaccine site has severe inflammation or ulceration after multiple vaccines, the next vaccine may be placed adjacent to the original site.

Chronic inflammatory reactions at the vaccine sites are expected to occur in all participants who receive Montanide ISA-51. Induration may persist for days to months, but is not expected to require additional therapy. Sterile abscesses may occur in some participants. These are not a basis for discontinuation of the vaccines. However, if the inflammatory reactions develop significant ulceration of the skin (> 2 cm), then this would be considered a dose-limiting toxicity and the patient would be taken off the study.

5.4.3 Vaccination doses:
Part 1:
For each vaccine, 300 mcg of each of the long peptides in LPV7 and 200 mcg of the tetanus helper peptide will be administered, plus 1 ml IFA or the dose of TLR agonists as specified in Table 1A and section 5.2.1.

Part 2:

For each vaccine, 300 mcg of each of the long peptides in LPV7 will be administered, plus 1 ml IFA and the dose of TLR agonists as specified in Table 1B and Section 5.2.1.

5.4.4 Route of administration:
At each injection site for emulsions with IFA, a single needle puncture is to be performed, with delivery of 1 cc volume into the subcutaneous tissue and 1 cc volume into the dermis. This usually will require advancing the needle into the subcutaneous tissue to deliver the first 1 cc over an area within reach of that needle, then pulling the needle back to near the skin puncture site, and advancing it into the intradermal tissue for delivery of the remaining 1 cc. For injections with peptides in polyICLC without IFA, the total volume will be 1 ml, which will be divided between subcutaneous tissue and dermis as above.

5.4.5 Post-Vaccination Observation
All participants will be closely observed for adverse events for at least 20 minutes following each vaccination. Any time thereafter, participants should report any adverse events to the research coordinator or research clinician.

5.4.6 Biopsies of Vaccine site and normal skin:
Each participant in Part 1 and Part 2 will undergo biopsy of the vaccine site at two time points (days 8 and 22; one week after vaccines 1 and 3). Control biopsies of clinically normal skin (three 4-mm punch biopsies of the dermis and subcutaneous tissue) will be taken at the same time points (days 8 and 22), for the first 6 participants enrolled in Part 1.

The biopsies will consist of three 4-mm punch biopsies of skin. Note that vaccine sites will be rotated to different skin sites for each vaccination; so there will not be repeat injection of vaccines into the prior skin biopsy sites.

5.4.6.1 The punch biopsies of normal skin will be taken from an upper thigh or upper arm, and will be preserved/processed as follows:

5.4.6.1.1 Normal 1: quick-frozen in liquid nitrogen.

5.4.6.1.2 Normal 2: preserved in formalin, then paraffin-embedded

5.4.6.1.3 Normal 3: preserved in RNA later.

5.4.6.2 The punch biopsy specimens of the VSME will be preserved/processed as follows:

5.4.6.2.1 VSME sample 1: quick-frozen in liquid nitrogen.

5.4.6.2.2 VSME sample 2: preserved in formalin, then paraffin-embedded

5.4.6.2.3 VSME sample 3: preserved in RNA later.

5.4.6.3 Their evaluation is summarized in the Study Manual.

5.4.6.4 The specimens should be put into liquid nitrogen, formalin, or RNA later within 15 minutes if at all possible, and ideally within 5 min of excision. Delays beyond 15 minutes should be documented. This information should be documented on the Skin Biopsy Submission Form.

5.5 Modification/Discontinuation of Treatment

5.5.1 Dose Modifications

There will be no dose modifications of the vaccine components.

5.5.2 Discontinuation

If participants are discontinued prior to one or more scheduled skin biopsies, they will not receive those biopsies. Protocol treatment will be discontinued for any of the following reasons:

5.5.2.1 Any dose-limiting toxicity as defined in Section 7.4.

5.5.2.2 In circumstances where assessment of an AE is limited, such as by intercurrent illness, or when laboratory studies are required to assess for other causes of toxicity, the vaccine/biopsy schedule may be interrupted for up to 7 days. Delay of one vaccine administration, biopsy, or evaluation visit by up to 7 days will not be considered a protocol violation, regardless of attribution. If more than one vaccine is delayed by 8 days or more, treatment must be discontinued.

5.5.2.3 Disease progression requiring other therapy (e.g. surgery under general anesthesia, radiation, chemotherapy, or steroid therapy). The appearance of small metastases or recurrent tumor deposits will not be a basis for discontinuing the vaccinations. Biopsy to determine the nature of new lesions, or minor surgical procedures to excise a new lesion, will not be a basis for discontinuing vaccinations.

5.5.2.4 Initiation of cytotoxic chemotherapy, radiation therapy, steroid therapy as detailed under Section 4.2.5, or other immunosuppressive therapy.

5.5.2.5 Any other potential adverse reaction deemed sufficiently serious to warrant discontinuation of therapy by the Principal Investigator or one of the sub-investigators.

5.5.2.6 Noncompliance with the requirements of the study.

5.5.2.7 Therapy may be discontinued at the participant's request.

5.5.2.8 Therapy may be discontinued at the discretion of an investigator.

5.5.2.9 Pregnancy. Pregnant participants will continue to be followed for the duration of the pregnancy.

5.5.3 Elective Withdrawal

A participant who is enrolled but neither receives any study drug nor has a skin biopsy on protocol may be replaced. Every attempt will be made to evaluate any data from these participants for endpoint assessment.

5.5.4 Delayed Visit for Reasons Other Than Toxicity

A schedule for return visits should be established at the first visit. If a participant misses a treatment, the missed treatment will be administered as soon as possible, so that the subsequent vaccinations are given in the appropriate intervals. Treatment may be continued for an additional time period, if needed. Participants who are vaccinated outside of the established schedule should return to the original schedule as soon as possible.

The table below defines what constitutes a delayed visit, whether the participant should continue to be treated, and whether a protocol violation/deviation should be reported and recorded. The range of days is counted from the original scheduled date.

Table 6. Delayed Visit for Reasons other than Toxicity

**Treatment Period**	**Range of Days**	**Participant Treatment**	**Protocol Deviation**
Vaccine 1			
Day 1	± 2 days	Vaccine/Labs	No
	± 3 to 7 days	Vaccine/Labs	Yes
	± 8 or more days	Labs	Yes
*Vaccine 2/Biopsy**			
Day 8	± 2 days	Vaccine/Labs/Biopsy	No
	± 3 to 7 days	Vaccine/Labs/Biopsy	Yes
	± 8 or more days	Labs	Yes
*Vaccine 3**			
Day 15	± 2 days	Vaccine/Labs	No
	± 3 to 7 days	Vaccine/Labs	Yes

	± 8 or more days	Labs	Yes
Assessment/Biopsy			
Day 22	± 2 days	Biopsy/Labs	No
	± 3 to 7 days	Biopsy/Labs	Yes
	± 8 or more days	Labs	Yes
Assessment			
Day 43			
	± 2 days	Labs	No
	± 3 to 7 days	Labs	Yes
	± 8 or more days	Labs	Yes
*Vaccines 4-6**			
Days 36, 57, 78	± 7 days	Vaccine/Labs	No
	± 8 to 14 days	Vaccine/Labs	Yes
	± 15 or more days	Labs	Yes
Assessment			
Day 85 (Week 12)	± 7 days	Labs/Scans	No
	± 8 to 14 days	Labs/Scans	Yes
	± 15 or more days	Labs	Yes
Follow-up			
Week 26, Year 1 and 2	± 7 days	Scans/Labs	No
	± 8 to 30 days	Scans/Labs	Yes
* A participant will be taken off protocol treatment if more than one vaccination is delayed [± 3 to 7 days] during the treatment period.			

5.6 <u>Concomitant Medications</u>

Medications taken in the month prior to randomization (Part 1) or arm assignment (Part 2) should be recorded on the baseline case report form. This includes prescription medications, over-the-counter medications, injected medications, biological products, blood products, imported drugs, or street drugs. Participants should be maintained on drugs that they were taking prior to entry unless a change in regimen is medically indicated.

5.6.1 Non-permitted medications or treatments (within 30 d of study treatment)

5.6.1.1 Systemic cytotoxic chemotherapy

5.6.1.2 Interferon therapy (e.g. Intron-A®)

5.6.1.3 Radiation therapy

5.6.1.4 Nitrosureas

5.6.1.5 Allergy desensitization injections

5.6.1.6 Systemic corticosteroids, administered parenterally or orally. Inhaled steroids (e.g. Advair®, Flovent®, Azmacort®) are not permitted.

5.6.1.7 Growth factors (e.g. Procrit®, Aranesp®, Neulasta®)

5.6.1.8 Interleukins (e.g. Proleukin®)

5.6.1.9 Other investigational medications

5.6.1.10 Street drugs

5.6.1.11 Antibodies to CTLA-4, PD-1, PD-L1, or CD137

5.6.1.12 Targeted molecular therapy (e.g. vemurafenib, other inhibitor of mutant BRAF, MEK, or cKit).

5.6.2 Permitted medications or treatments

5.6.2.1 Nonsteroidal anti-inflammatory agents

5.6.2.2 Anti-histamines (e.g. Claritin®, Allegra®)

5.6.2.3 Topical corticosteroids are acceptable, including steroids with very low solubility administered nasally for local effects only (e.g. Nasonex®).

5.6.2.4 Short-term therapy for acute conditions not specifically related to melanoma (except those listed under Section 5.6.1)

5.6.2.5 Chronic medications except those listed in Section 4.2.7 or 5.6.1.

5.6.2.6 Vaccines, including live vaccines (e.g. FluMist) are permitted, but should be administered at least 2 weeks prior to or at least 2 weeks after a study vaccine.

5.6.2.7 Topical forms of chemotherapeutic reagents (e.g. Efudex®) are permitted, but should be administered at least 2 weeks after a study vaccine.

6.0 CLINICAL AND LABORATORY EVALUATION

6.1 Screening

The following studies should be completed within 6 weeks prior to randomization unless otherwise noted:

6.1.1 Class I HLA-typing (any time prior to randomization; typing for HLA-A and HLA-B is sufficient). If the patient is known to express HLA-A1, A2, or A3, HLA-B testing is not required).

6.1.2 Review of pathology at the treating and participating institution (any time prior to randomization). Central pathology review at UVA is not required for pathology reviewed at other participating institutions.

6.1.3 CBC with differential, including automated lymphocyte count if available (0.3 ml)

6.1.4 Comprehensive chemistry panel to include sodium, potassium, creatinine, **fasting** glucose, calcium, total bilirubin, AST, ALT, and alkaline phosphatase. NOTE: fasting blood sugars may be evaluated 4h or more after last eating (0.9 ml)

6.1.5 Lactate Dehydrogenase (LDH) (0.3 ml)

6.1.6 Urinalysis

6.1.7 β-HCG for women of childbearing potential (within two weeks prior to randomization)

6.1.8 HgB-A1C (only required at baseline)

6.1.9 HIV and HCV screening (within 6 months prior to randomization)

6.1.10 Chest x-ray or chest CT, abdominal and pelvic CT, and head CT or MRI. PET/CT fusion scan may replace scans of the chest, abdomen, and pelvis.

6.1.11 Complete history and physical examination, including:

- Vital signs
- Weight
- Performance Status
- Assessment of skin and nodal basins for evidence of disease recurrence or metastasis
- Designation of vaccination sites
- Medication review

6.2 <u>Treatment</u>

The following evaluations will be performed on an outpatient basis on each of the listed days. Blood for these evaluations should be obtained prior to the vaccine injection if a vaccine is scheduled to be administered. Results of those tests are not required prior to administering the vaccine on that date.

6.2.1 Day 1

- Interval history and physical examination directed at new signs and symptoms.
- Vital signs
- Weight
- Performance status
- Visual acuity exam using a Snellen chart, and color vision exam using an Ishihara eye chart
- Neurologic function, general.
- Note hair and eye color
- Assessment of skin for vitiligo
- Anti-nuclear antibody and rheumatoid factor (4 ml total)
- Baseline symptoms, codable in the NIH CTCAE v.4.03, to be recorded as pre-existing conditions unrelated to the research protocol interventions
- Distribute participant toxicity diary
- Medication review
- Blood collection for immunologic testing:_The following blood samples for research will be provided to UVA and processed by the UVA BTRF and stored by the UVA-HITC IML:
  - 120 cc blood collected in heparinized green top tubes for lymphocytes. This may be decreased to 100 cc if more blood must be drawn for safety or screening labs, in order to comply with institutional limits on total blood drawn in a day.
  - 20 cc blood collected in red top tubes for serum
- Vaccine administration

6.2.2 Day 8

- Interval history and physical examination directed at new signs and symptoms. Examination of the vaccine site(s) should be included..

- Vital signs
- Weight
- Performance status
- Review of adverse events
- Review of participant toxicity diary and distribute new toxicity diary
- Medication review
- Blood collection for immunologic testing:_The following blood samples for research will be provided to UVA and processed by the UVA BTRF and stored by the UVA-HITC IML:
    - 80 cc blood collected in heparinized green top tubes for lymphocytes.
    - 20 cc blood collected in red top tubes for serum
- Biopsies of 1st vaccine site (all patients), and biopsies of normal skin (patients 1-6 only in Part 1). Details in section 5.4.6 and in Study Manual.
- Vaccine administration

6.2.3 Day 15

- Interval history and physical examination directed at new signs and symptoms. Examination of the vaccine site(s) should be included.
- Vital signs
- Weight
- Performance status
- Review of adverse events
- Review of participant diary and distribute new toxicity diary
- Medication review
- Vaccine administration

6.2.4 Day 22

- Interval history and physical examination directed at new signs and symptoms. Examination of the vaccine site(s) should be included.
- Vital signs
- Weight
- Performance status
- Review of adverse events
- CBC with differential, including automated lymphocyte count if available (0.3 ml)
- Comprehensive chemistry panel to include sodium, potassium, creatinine, glucose, calcium, total bilirubin, AST, ALT, and alkaline phosphatase. (0.9 ml)
- Lactate Dehydrogenase (LDH) (0.3 ml)
- Anti-nuclear antibody and rheumatoid factor (4 ml total)
- Assessment of skin for vitiligo
- Neurologic function
- Vaccine Site Biopsy (3)
- Review of participant diary and distribute new toxicity diary
- Medication review

- Blood collection for immunologic testing:_The following blood samples for research will be provided to UVA and processed by the UVA BTRF and stored by the UVA-HITC IML:
- 80 cc blood collected in heparinized green top tubes for lymphocytes.
- 20 cc blood collected in red top tubes for serum
- Biopsies of 3rd vaccine site (all patients), and biopsies of normal skin (patients 1-6 only, in Part 1). Details in section 5.4.6 and in Study Manual.

6.2.5 Day 36

- Interval history and physical examination directed at new signs and symptoms. Examination of the vaccine site(s) should be included.
- Vital signs
- Weight
- Performance status
- Review of adverse events
- Review of participant diary and distribute new toxicity diary
- Medication review
- Vaccine administration

6.2.6 Day 43

- Interval history and physical examination directed at new signs and symptoms. Examination of the vaccine site(s) should be included.
- Vital signs
- Weight
- Performance status
- Review of adverse events
- Review of participant diary and distribute new toxicity diary
- Medication review
- Blood collection for immunologic testing:_The following blood samples for research will be provided to UVA and processed by the UVA BTRF and stored by the UVA-HITC IML:
    - 80 cc blood collected in heparinized green top tubes for lymphocytes.
    - 20 cc blood collected in red top tubes for serum

6.2.7 Day 57

- Interval history and physical examination directed at new signs and symptoms. Examination of the vaccine site(s) should be included.
- Vital signs
- Weight
- Performance status
- Review of adverse events
- Review of participant diary and distribute new toxicity diary
- Medication review
- Vaccine administration

6.2.8 Day 78

- Interval history and physical examination directed at new signs and symptoms. Examination of the vaccine site(s) should be included.
- Vital signs
- Weight
- Performance status
- Review of adverse events
- CBC with differential, including automated lymphocyte count if available (0.3 ml)
- Comprehensive chemistry panel to include sodium, potassium, creatinine, glucose, calcium, total bilirubin, AST, ALT, and alkaline phosphatase. (0.9 ml)
- Lactate Dehydrogenase (LDH) (0.3 ml)
- Anti-nuclear antibody and rheumatoid factor (4 ml total)
- Review of participant diary and distribute new toxicity diary
- Medication review
- Vaccine administration

6.2.9 Day 85

- Interval history and physical examination directed at new signs and symptoms. Examination of the vaccine site(s) should be included.
- Vital signs
- Weight
- Performance status
- Review of adverse events
- CBC with differential, including automated lymphocyte count if available (0.3 ml)
- Comprehensive chemistry panel to include sodium, potassium, creatinine, glucose, calcium, total bilirubin, AST, ALT, and alkaline phosphatase. (0.9 ml)
- Lactate Dehydrogenase (LDH) (0.3 ml)
- Chest X-ray or chest CT
- Visual acuity exam using a Snellen chart, and color vision exam using an Ishihara eye chart
- Neurologic function
- Note hair and eye color
- Assessment of skin for vitiligo
- Review of participant diary
- Medication review
- Blood collection for immunologic testing:_The following blood samples for research will be provided to UVA and processed by the UVA BTRF and stored by the UVA-HITC IML:
  - 80 cc blood collected in heparinized green top tubes for lymphocytes.
  - 20 cc blood collected in red top tubes for serum

6.2.10 Week 26

- Interval history and physical examination directed at new signs and symptoms. Examination of the vaccine site(s) should be included.

- Vital signs
- Weight
- Performance status
- Anti-nuclear antibody and rheumatoid factor (4 ml total)
- Chest x-ray or chest CT
- Neurologic function
- Assessment of skin for vitiligo
- Medication review
- Blood collection for immunologic testing:_The following blood samples for research will be provided to UVA and processed by the UVA BTRF and stored by the UVA-HITC IML:
  - 80 cc blood collected in heparinized green top tubes for lymphocytes.
  - 20 cc blood collected in red top tubes for serum

6.2.11 Weeks 52 and 104 (Year 1 and 2)

- Interval history and physical examination directed at new signs and symptoms. Examination of the vaccine site(s) should be included.
- Vital signs
- Weight
- Performance status
- Medication review
- Blood collection for immunologic testing:_The following blood samples for research will be provided to UVA and processed by the UVA BTRF and stored by the UVA-HITC IML:
  - 80 cc blood collected in heparinized green top tubes for lymphocytes.
  - 20 cc blood collected in red top tubes for serum

6.3 Tumor tissue collection

If during the study, participants develop metastases or recurrences, these may be removed, and following receipt by pathology, may be evaluated by the study research team. Tissue samples may be screened for antigen expression or protein profiles using tests such as Western blots, immunohistochemistry, PCR, flow cytometry or gene chip analysis. Tumor escape mechanisms may also be evaluated. Specimens will be used in immunological assays to assess T cell function or antibody response. Assays generally used for this type of testing include, but are not limited to, ELIspot assays, ELISAs, chromium-release assays, proliferation assays and intracellular cytokine staining. Specimens may be used to study the immunologic aspects of the tumor microenvironment or as targets or controls in laboratory assays. Specimens may be used to establish cell lines for long-term studies.

This tissue may also be compared to lesions resected prior to enrollment, which will be requested from the pathology department of each institution as paraffin-embedded tissue samples, and these tissues may be banked for use in future studies. If participants are removed from the study or progress during or after follow-up, tissue may be collected for use as part of this study, as described above, or banked for use in future studies

7.0 REGULATORY AND REPORTING REQUIREMENTS

Adverse event reporting requirements for this trial, as specified in this section, apply to patients accrued at all participating sites. There will be accrual at UVA and at MD Anderson

Cancer Center; UVA will be the coordinating site, and responsibility for data coordination and reporting for the study as a whole falls to the Human Immune Therapy Center (HITC). Throughout this section, when referring to 'Cancer Center Database', 'Cancer Center DSMC', 'CRC', 'PI' or, 'nurse practitioner', they are preceded by 'site' when it refers to reporting within either of the participating institutions, or are preceded by 'UVA' when it refers to central reporting to the coordinating UVA center.

In addition to the reporting requirements for the study, specified in this section of the protocol, investigators should also follow their institutional policy for documentation in their database and reporting to the IRB locally at their own site.

7.1 Definitions

7.1.1 Adverse event (AE) –

Any unfavorable and unintended sign (including an abnormal laboratory finding), symptom, or disease temporally associated with the use of a medical treatment or procedure regardless of whether it is considered related to the medical treatment or procedure (attribution of unrelated, unlikely, possible, probable, or definite). Medical conditions or diseases present before starting the investigational drug will be considered as treatment-related AEs if they worsen after starting study treatment.

7.1.2 Unexpected AE –

Any adverse event not listed in Section 7.4.3.

7.1.3 Serious AE –

Any adverse drug experience occurring at any dose that results in any of the following outcomes:

- death;
- a life-threatening adverse drug experience;
- inpatient hospitalization, or prolongation of existing hospitalization (as defined below in this section);
- a persistent or significant disability/incapacity; or a congenital anomaly/birth defect.
- Important medical events that may not result in death, be life threatening, or require hospitalization may be considered a serious adverse drug experience when, based upon medical judgment, they may jeopardize the patient or subject and may require medical or surgical intervention to prevent one of the outcomes listed in this definition.

Hospitalization for expedited AE reporting purposes is defined as an inpatient hospital stay equal to or greater than 24 hours. Hospitalization is used as an indicator of the seriousness of the adverse event and should be reserved for situations where the adverse event truly fits this definition and not for hospitalizations associated with less serious events. For example, a hospital visit where a patient is admitted for observation or minor treatment (e.g. hydration) and released in less than 24 hours would not be considered serious. Furthermore, hospitalization for pharmacokinetic sampling is not an AE, and therefore is not to be reported either as a routine AE or in an expedited report.

7.1.4 Unanticipated problem –

An unanticipated problem is any event/experience that meets ALL 3 criteria below:

- Is unexpected in terms of nature, severity or frequency given the research procedures that are described in the protocol-related documents AND in the characteristics of the subject population being studied.
- Is related or possibly related to participation in research. This means that there is a reasonable possibility that the incident may have been caused by the procedures involved in the research study. (see section 7.2)
- The incident suggests that the research placed the subject or others at greater risk of harm than was previously known or recognized OR results in actual harm to the subject or others.

7.1.5 Protocol Violation-

A protocol violation is defined as any change, deviation, or departure from the study design or procedures of a research project that is NOT approved by the institution's IRB prior to its initiation or implementation, OR deviation from standard operating procedures, Good Clinical Practices (GCPs), federal, state or local regulations. Protocol violations may or may not be under the control of the study team or UVa staff. These protocol violations may be major or minor violations.

7.1.6 Suspected Adverse Reaction (as defined in 21 CFR 312.32 (a))-

Any adverse event for which there is a reasonable possibility that the drug caused the adverse event.

7.2 Attribution Assessment

7.2.1 Attribution – The determination of whether an adverse event is related to a medical treatment or procedure. The attribution groups are:

Definite – Applies to those adverse events which, the investigator feels are incontrovertibly related to the vaccine. An adverse event may be assigned an attribution of definitely related if or when (must have all of the following):

- It follows a reasonable temporal sequence from administration of the test drug.
- It could not be reasonably explained by the known characteristics of the subject's clinical state, environmental or toxic factors, or other modes of therapy administered to the subject.
- It disappears or decreases on cessation or reduction in dose with re-exposure to drug. (Note: This is not to be constructed as requiring re-exposure of the subject; however, the group of definitely related can only be used when a recurrence is observed.)
- It follows a known pattern of response to the test drug.

Probable – Applies to those adverse events for which, after careful consideration at the time they are evaluated, are felt with a high degree of certainty to be related to the test drug. An adverse event may be considered probably related if or when (must have three of the following):

- It follows a reasonable temporal sequence from administration

of the test drug.

- It could not be reasonably explained by the known characteristics of the subject's clinical state, environmental or toxic factors, or other modes of therapy administered to the subject.
- It disappears or decreases on cessation or reduction in dose. There are important exceptions when an adverse event does not disappear upon discontinuation of the drug, yet drug-relatedness clearly exists (e.g. bone marrow depression, fixed drug eruptions, tardive dyskinesia).
- It follows a known pattern of response to the test drug.

Possible – Applies to those adverse events for which, after careful consideration at the time they are evaluated, a connection with the test drug administration appears unlikely but cannot be ruled out with certainty. An adverse event may be considered possibly related if or when (must have two of the following):

- It follows a reasonable temporal sequence from administration of the test drug.
- It could not readily have been produced by the subject's clinical state, environmental or toxic factors, or other modes of therapy administered to the subject.
- It follows a known pattern of response to the test drug.

Unlikely – Applies to those adverse events for which, after careful consideration at the time they are evaluated, are judged to be unrelated to the test drug. An adverse event may be considered unlikely if or when (must have two of the following):

- It does not follow a reasonable temporal sequence from administration of the test drug.
- It could readily have been produced by the subject's clinical state, environmental or toxic factors, or other modes of therapy administered to the subject.
- It does not follow a known pattern of response to the test drug.
- It does not reappear or worsen when the drug is re-administered.

Unrelated – Applies to those adverse events, which after careful consideration, are clearly and incontrovertibly due to extraneous causes (disease, environment, etc.).

7.3 Data collection

Data will be collected using a centralized electronic case report form called **ON**-line **C**linical **O**ncology **R**esearch **E**nvironment = **Oncore**.

7.4 Risks and Safety

7.4.1 Adverse Event Descriptions and Grading Scales

The NCI Common Terminology Criteria for Adverse Events (CTCAE) version 4.03 will be used for the characterization and grading of adverse events (see Appendix 6).

7.4.2 Time Span for Reporting Adverse Events

Reporting of AEs will begin when the subject is administered the study drug or has a study related biopsy. Events occurring through 30 days after administration of the last vaccine, regardless of attribution, will be reported. AEs should be followed to resolution or stabilization. If an AE worsens and becomes an SAE, it should be reported as serious per the guidelines specified for SAE reporting.

AEs that are possibly, probably, or definitely related to the vaccine will be recorded until the subject completes treatment follow-up. If, during treatment follow-up, the subject receives additional treatment, subjects will be off treatment follow-up and will be followed yearly for disease progression and survival.

7.4.3 Agent-Specific Expected Adverse Events List:

Any AE not on this list will be considered an unexpected AE.

Expected toxicities related to peptide vaccines. This list of expected AEs for the LPV7 vaccine is based on prior data from the UVA-Mel 44 clinical trial in which 41 participants in Arm A received a mixture of 12 Class I peptides plus a tetanus helper peptide in an emulsion with Montanide ISA-51 adjuvant. AEs are listed only if observed in ≥ 5% of treated participants.

Table 7: Summary Toxicity Data from UVA-Me44
Grade 2:

**CTCAE Category**	**Description**
Investigations	Lymphocyte count decreased
General disorders and administration site conditions	Fatigue
General disorders and administration site conditions	Fever
Skin and subcutaneous tissue disorders	Hyperhidrosis
Skin and subcutaneous tissue disorders	Skin induration
Skin and subcutaneous tissue disorders	Skin ulceration
General disorders and administration site conditions	Injection site reaction
Gastrointestinal Disorders	Nausea
Metabolism and nutrition disorders	Hyperglycemia
General disorders and administration site conditions	Flu-like symptoms
Skin and subcutaneous disorders	Other: any rash

*NOTE: Hyperglycemia ≥ Grade 3 is not an expected event. If a non-fasting hyperglycemia adverse event occurs at a level ≥ Grade 3, a fasting serum glucose test will be ordered and the event will be re-graded appropriately. The fasting serum glucose measurement will be used for the purpose of determining reporting requirements.

Injection site reaction/induration was experienced at maximum grade 3 in 5% of patients in Arm A of Mel44. Ulceration was experienced as maximum grade 3 in 7% of patients in Arm A of Mel 44. By the rule above (observed in ≥5% of treated patients), injection site reaction, induration, and ulceration would be expected at grade 3. Despite this and in the interest of the patients, we would consider grade 3 injection site reaction with significant ulceration of the skin (> 2 cm) as a DLT (see section 7.8).

Adverse Events Expected with Resiquimod application at the vaccine site.

The investigator brochure specifies the following adverse events in more than 1800 patients treated with topical resiquimod in 25 clinical studies. Reported adverse events were primarily local adverse events including protocol-defined local skin reactions (including, erythema, edema, ulceration, vesicles, weeping, scabbing, and flaking); and protocol-defined local symptoms at the site of administration (including pain, burning, pruritus, and numbness/tingling). Other adverse events at the application site include drainage, infection, and pigmentation changes. Systemic adverse events that may be related to resiquimod due to a systemic induction of cytokines and/or release of locally induced cytokines into the circulation include influenza-like symptoms (e.g., fever, headache, arthralgia, myalgia, chills, asthenia, fatigue), and transient decreases in neutrophils and total leukocytes that may be related to cellular trafficking to the application site. Other infrequent adverse events that were considered possibly or probably related to study medication included liver function test abnormalities, abdominal cramping, nausea, vomiting, diarrhea, backache, neck pain, rhinitis, urticaria, and dizziness.

The formal list, below, of expected AEs for resiquimod, for this study, is based on published data related to its clinical use [65;66] The table below is based on a study applying resiquimod 3 times per week for 4 weeks, for treatment of actinic keratosis. This study had 4 treatment arms, using 0.01, 0.03, 0.06, and 0.1% resiquimod gel. We will be using 0.2% resiquimod gel, only once a week; so toxicities from 3x/weekly administration with 0.06% resiquimod use may exceed what we observe[65]. Overall in that study, no deaths or serious adverse events were reported, but some adverse events are described as severe. We expect grade 2 toxicities in these categories. Listed in the table below are those categories where adverse events were recorded in 10% of patients or more, in any study group, but the percentages listed are those for patients receiving resiquimod 0.06% three times per week.

Table 8: Toxicity Data for Resiquimod

Grade 2:

**CTCAE Category**	**Description**	**Percent with possibly or probably related AEs at 0.6% resiquimod three times per week**
General disorders and administration site conditions	Application site burning	25%
	Application site erythema	6%
	Application site pain	16%
	Application site pruritus	25%
	Application site reaction	None
	Fatigue	9%
	Rigors/chills	16%
Musculoskeletal and connective tissue disorders	Arthralgia	16%
	Myalgia	6%
Nervous system disorders	Headache	19%
	Lethargy/fatigue	13%
Psychiatric disorders	Not specified	16%
Skin and subcutaneous tissue disorders	Not specified	13%

Mild and transient leukopenia and thrombocytopenia have also been reported with use of resiquimod, but these are expected only to be grade I toxicities.

Toxicities at sites of resiquimod application should be limited to grade 1, 2, or 3 (with ulceration ≤ 2 cm) dermatologic toxicities, and should be manageable either by continuation of resiquimod if the patient is tolerating the toxicities and if the toxicities are relatively stable, or they may be managed by holding vaccination for one to several days (up to 7 days without being a DLT).

Adverse events expected with polyICLC (Hiltonol)

The following expected toxicities for polyICLC are based on the data from 45 patients treated with 20 mcg/kg 3x/week. This would be about 1.4 mg per dose and 4.2 mg per week (much higher than we will use).

Table 9: Toxicity Data for PolyICLC

Category	Toxicity	Grade	Comment
Nervous system disorders	Headache	Grade 2	1 of 22 (<5%)
	Tremors	Grade 3	2 of 45 (4%) poss related
Musculoskeletal and connective tissue disorders	Muscle weakness	Grade 2	1 of 45 (<2%) experienced at Grade 3
Respiratory, thoracic and mediastinal disorders	Dyspnea	Grade 2	1 of 45 (<2%) experienced at Grade 3
	Hypoxia	Grade 2	1 of 45 (<2%) experienced at Grade 3
Metabolism and nutrition disorders	Hypernatremia	Grade 2	1 of 45 (<2%) experienced at Grade 3
Investigations	Elevated transaminases (GPT)	Grade 3	4 of 45 (9%) 3 cases possibly related; one case probably related. Typically transient.
	Elevated Alkaline phosphatase	Grade 3	7% of patients, in IB
	Leukocytopenia	Grade 3	2 of 45 (<4%) in published work; 20% in IB
	Thrombocytopenia	Grade 3	14% of patients in IB
	Neutropenia	Grade 3	10% of patients in IB
Blood/lymph disorders	Anemia	Grade 3	13-31% of patients in IB
General disorders and administration site conditions	Vaccine site reaction	Grade 2	1 of 22 (<5%)
	Fever	Grade 3	14% of patients in RCC trial
	Chills	Grade 3	10% of patients in RCC trial
	Fatigue	Grade 3	10% of patients in RCC trial

Adverse events expected from vaccine and normal skin biopsies.

Below is a list of expected AEs related to skin biopsies:

Table 10: Toxicity Data for Skin Biopsies

Grade 2:

CTCAE Category	Description
Pain	Pain - Skin

7.5 Adverse Event Classifications

The determination under which adverse event classification an AE is reported. The adverse event classifications are specified in the CTCAE v4.03. For specific classifications pertaining to the protocol, we specify the following:

Hematologic/Metabolic- Any AE coded under one of the following CTCAE v4.03 categories should be reported under the Hematologic/Metabolic adverse event classification:

Table 11: Hematologic/Metabolic Classifications

Section	AE
Blood and lymphatic	Anemia<br>Leukocytosis
Investigations	**ALL EXCEPT:**<br>Carbon monoxide diffusing capacity decreased<br>Ejection fraction decreased<br>Forced expiratory volume decreased<br>Vital capacity abnormal<br>Weight gain<br>Weight loss
Metabolism and nutrition disorders	**ALL EXCEPT:**<br>Alcohol intolerance<br>Anorexia<br>Dehydration<br>Glucose intolerance<br>Iron overload<br>Obesity<br>Tumor lysis syndrome

Non-hematologic/Non-Metabolic- Any AE not reported under hematologic/metabolic, ocular, or allergic/autoimmune, should be reported under the non-hematologic/non-metabolic adverse event classification.

Ocular – Any AE coded under one of the following CTCAE v4.03 Adverse Event Terms should be reported under the Ocular adverse event classification:

1) A single treatment-related experience of the following adverse events will be classified as a DLT:
   - Eye Disorders: Night blindness (nyctalopia)
   - Eye Disorders: Papilledema
   - Eye DisordersI: Retinopathy

   Participants will be referred for an ophthalmologic exam if any of these ocular adverse events occur.

2) A prolonged treatment-related experience (e.g., lasting > 5 days) of the following non-severe adverse events will be classified as a DLT:
   - Eye Disorders: Blurred vision
   - Eye Disorders: Flashing lights
   - Eye Disorders: Floaters

   Participants will be referred for an ophthalmologic exam if any of these ocular adverse events occur.

Allergic/Autoimmune – Only AEs coded as Immune System Disorder: Allergic reaction, autoimmune disorder, or anaphylaxis should be reported under the Allergic/Autoimmune

adverse event classification. Other AEs coded under Immune System Disorder should be reported under Non-hematologic/Non-metabolic adverse event classification.

7.6 Reporting Adverse Events

7.6.1 Process for Reporting AEs:

The process of reporting AEs will occur as follows:

A. Identify the type of event using the NCI CTCAE v4.03 (Appendix 6). The CTCAE provides descriptive terminology and a grading scale for each adverse event listed.
B. Grade the event using the NCI CTCAE v4.03.
C. Determine whether the AE is related to the vaccine. Attribution groups are defined in Section 7.2.1.
D. Determine the prior experience of the AE. Expected events are those that have been previously identified as resulting from administration of the vaccine. An AE is expected when it is listed in Section 7.4.3.
E. Determine the adverse event classification of the AE, as specified and defined in Section 7.5.
F. Determine whether the AE is a dose-limiting toxicity and report as specified below.
G. Determine whether the AE is an SAE and report as specified below.
H. AEs determined to require expedited reporting to the Sponsor, UVA HITC, must also be reported to your own institution according to the local policy and procedures.

Reporting Dose Limiting Toxicities

All DLT's (defined in Section 7.8) should be reported by phone or fax to the HITC within 24 hours of when the site is notified of the event and entered into Oncore within 5 calendar days of when the site is notified of the event.

DLT's that are deemed serious and unexpected should be submitted to to a site's local IRB per their institutional guidelines.

SAE reporting

- **Report SAEs by phone or fax to the HITC within 24 hours of of when the site is notified of the event.**
- **Send completed FDA MedWatch 3500a form and relevant supporting documentation (e.g. clinic notes, hospital case records) to the Sponsor within 3 calendar days for deaths or life-threatening events and 5 calendar events for other SAEs.**
- **Enter SAE into Oncore per guidelines specified in the table below.**

DLT and SAE Reporting Information:
UVA HITC
Phone: (434) 982-6714
Fax: (434) 982-6608
Box 801457, 1352 Jordan Hall
Charlottesville, VA 22908

In addition to DLT's and SAE's, all other AEs must be recorded into the University of Virginia Cancer Center OnCore database per the following guidelines:

Table 12: AE reporting

High Risk Studies<br>Reporting requirements for AEs that occur within 30 days of the last dose of protocol specified treatment								
	Grade 1	Grade 2		Grade 3				Grade 4 & 5
	Expected and unexpected	Expected	Unexpected	Expected		Unexpected		Expected and Unexpected
				Without hospitalization	With hospitalization	Without hospitalization	With hospitalization	
Unrelated Unlikely	OnCore 30 days[a]	OnCore 30 days	OnCore 30 days	OnCore 30 days	OnCore 15 days	OnCore 30 days	OnCore 15 days	OnCore 7 days
Possible Probable Definite	OnCore 30 days[a]	OnCore 30 days	OnCore 15 days	OnCore 30 days	OnCore 15 days	OnCore 7 days	OnCore 7 days	OnCore (24-hrs)* 7 days

*Enter into OnCore database w ithin 24 hours if unexpected and definitely related to protocol specified treatment
Hospitalization defined as an inpatient hospital stay or prolongation of a hospital stay equal to or greater than 24 hours
[a] Grade 1 unexpected or expected hematologic/metabolic events w ill be recoreded in the Cancer Center Database; how ever, regardless of attribution, these events do not have to be reported.

Pregnancy

- Report pregnancy to the Sponsor w ithin 5 calendar days of w hen the site is notified of the event.
- Report the pregnancy using the Safety Reporting Cover Sheet.

Pregnancy Reporting Information:
UVA HITC
Phone: (434) 982-6714
Fax: (434) 982-6608
Box 801457, 1352 Jordan Hall
Charlottesville, VA 22908

7.6.2 IRB Reporting Requirements

Participating sites should follow their institutional policies for reporting adverse events and serious adverse events to their local Institutional Review Board.

The University of Virginia is responsible for reporting to the UVA IRB-HSR per the follow ing guidelines:

Table 13: UVA IRB-HSR reporting

Type of Event	To whom will it be reported:	Time Frame for Reporting	How reported?
Any internal event resulting in death that is deemed DEFINITELY related to (caused by) study participation	IRB-HSR	Within 24 hours	IRB Online and phone call<br><br>www.irb.virginia.edu/

Type of Event	To whom will it be reported:	Time Frame for Reporting	How reported?
(Note: An internal event is one that occurs in a subject enrolled in a UVa protocol.)			
Internal, Serious, Unexpected adverse event.	IRB-HSR	Within 7 calendar days from the time the study team received knowledge of the event.<br><br>*Timeline includes submission of signed hardcopy of AE form.*	IRB Online<br><br>www.irb.virginia.edu/
Unanticipated Problems that are not adverse events or protocol violations This would include a Data Breach.	IRB-HSR	Within 7 calendar days from the time the study team received knowledge of the event.	Unanticipated Problem report form.<br><br>*http://www.virginia.edu/vprgs/irb/HSR_docs/Forms/Reporting_Requirements-Unanticipated_Problems.doc*)
Protocol Violations (*The IRB-HSR only requires that MAJOR violation be reported, unless otherwise required by your sponsor, if applicable.)*<br><br>Or<br><br>Enrollment Exceptions	IRB-HSR	Within 7 calendar days from the time the study team received knowledge of the event.	Protocol Violation and Enrollment Exception Reporting Form<br><br>http://www.virginia.edu/vpr/irb/hsr/
Data Breach	The UVa Corporate Compliance and Privacy Office and<br><br>ITC: if breach involves electronic data-<br><br>UVa Police if breach includes such things as stolen computers.	As soon as possible and no later than 24 hours from the time the incident is identified.<br><br>As soon as possible and no later than 24 hours from the time the incident is identified.<br><br>IMMEDIATELY.	UVa Corporate Compliance and Privacy Office- Phone 924-9741<br><br>ITC: Information Security Incident Reporting procedure, http://www.itc.virginia.edu/security/reporting.html<br><br>Phone- (434) 924-7166

7.6.3 Additional Reporting Requirements for the Sponsor (UVA)

Reporting to the FDA

Serious and unexpected suspected adverse reactions will be reported to the FDA no later than 15 calendar days after the sponsor determines that the requirements for an IND safety report have been met. The FDA will be notified using an FDA Form 3500a.

Unexpected fatal or life-threatening suspected adverse reactions will be reported to the FDA no later than 7 calendar days after the Sponsor receives the initial information of the event. The FDA will be notified using an FDA Form 3500a.

Other adverse event information will be sent to the FDA in the IND annual report.

Reporting to the Sites

The sponsor will report serious and unexpected suspected adverse reactions to the outside sites no later than 15 calendar days after the sponsor determines that the requirements for an IND safety report have been met.

7.6.4 Reporting of Subject Withdrawals/Dropouts Prior to Study Completion

Subjects who withdraw consent and those dropping out of the study secondary to an AE will be reported to the UVA IRB yearly on the IRB continuation form. These data should be reported to the sponsor within 30 days of subject withdrawal or dropout.

7.7 Adverse Event Review and Monitoring

7.7.1 In addition to clinic notes, adverse events will be initially captured using study-specific tools and participant toxicity diaries.

As specified in the study flow chart (Appendix 1), each participant will be evaluated by a licensed clinician. The following will be performed as designated in the protocol: routine disease-directed physical exam including performance status, blood collection, re-assessment for vitiligo, and examination of the skin and nodal basins for evidence of metastasis.

Participants will keep a daily diary of toxicities for days 1 through 85. The diaries will be reviewed by a research clinician prior to the next scheduled vaccine. During clinic visits, participants will also be asked about subjective symptoms including headache, malaise, fatigue, dyspnea, nausea, rash, diarrhea, abdominal discomfort, peripheral nerve pain, visual changes, appetite, tremors, night sweats, and ability to concentrate. Additional toxicities will be captured from laboratory tests. For each AE (with the exception of Grade 1 hematologic/metabolic events), date of onset, duration, grade, and attribution will be noted in the participant's study chart, on study documents, or in the clinic note, and will be entered into the UVA Cancer Center database.

After each vaccination, participants will be observed for AEs for at least 20 minutes. Follow-up phone calls will be made per the judgment of the research clinicians with regard to individual participant need. Subjects will be instructed on how to reach their provider should they have any questions and/or problems during the study.

In the event of an AE, appropriate action will be taken to ensure adequate care for the participant. If the participant is still on protocol, treatment delay or withdrawal from the protocol will be considered according to the protocol guidelines.

7.7.2 Individual AEs will be reviewed at each site by the Principal Investigator, nurse practitioner(s), and clinical research coordinator(s) (CRC),.

7.7.3 SAEs experienced by participants at all sites will be reviewed twice a month by the sponsor during the UVA Melanoma Team meeting. This meeting will occur at least 20 times in a calendar year. Those present at the meeting include the sponsor/overall study PI, sub-investigators, protocol development staff, biostatisticians, research nurses, research coordinators, laboratory specialists, and laboratory research managers. These meetings may also include the review of individual participants to assess whether they are protocol candidates, whether AEs warrant discontinuation, and whether existing protocols should be continued or closed.

7.7.4 Monthly conference calls will be held with the Sponsor and the site designee to review protocol status, accruals, and adverse events.

7.7.5 The following laboratory values will be recorded in the UVA Cancer Center database, graded using the CTCAE v4.03 (if a grading category exists), and reported as described in Section 7.6:

1. Alk Phosphatase
2. ALT (GPT)
3. ANA
4. AST (GOT)
5. Bilirubin, total
6. Creatinine
7. Eosinophil #
8. Hepatitis C serology
9. beta-HCG
10. HgB
11. HIV
12. HLA type
13. LDH
14. Potassium
15. RF
16. Urinalysis
17. WBC

Any abnormal laboratory values captured which are not included in the above list, but are considered to be pertinent positive clinical signs/symptoms, and laboratory results obtained as part of routine care of patients will be recorded in the UVA Cancer Center database and reported as described in Section 7.6. If there is any doubt on the part of study personnel concerning what constitutes a pertinent positive finding, the sponsor is to be consulted.

7.8 <u>Adverse Event Stopping Guidelines</u>
Based on results from previous vaccinations with 12-MP and tetanus peptide preparations, and with findings from use of resiquimod and polyICLC, significant toxicity is not anticipated. The study will be monitored continuously for treatment-related adverse events within each vaccine preparation by the sponsor. Data from the UVA- Mel44 vaccine trial (Arm A) (using 12-MP and tetanus peptide) indicate an 8% rate of treatment-related unexpected Grade 3 adverse events.

Adverse events will be described and coded based upon the NCI CTCAE v4.03.

A DLT is defined as any unexpected adverse event that is possibly, probably or definitely related and:

- ≥ Grade 3, with the exception of grade 3 injection site reaction with ulceration ≤ 2 cm
- ≥ Grade 1 ocular adverse events as defined under Section 7.5
- ≥ Grade 2 allergic/autoimmune reactions

7.9 Responsibility

7.9.1 The University of Virginia Cancer Center Data and Safety Monitoring Committee (DSMC) will provide oversight of the conduct of this study. The CC DSMC will report to the UVA Protocol Review Committee (PRC).

7.9.2 The UVA CC DSMC will review the following:

All adverse events
Audit results
Application of study designed stopping/decision rules
Whether the study accrual pattern warrants continuation/action
Protocol violations

7.9.3 The UVA CC DSMC will meet every month for aggregate review of data. Tracking reports of the meetings are available to the PI for review. Issues of immediate concern by the DSMC are brought to the attention of the sponsor (and if appropriate to the PRC and IRB) and a formal response from the sponsor is requested. Per the UVA Cancer Center NIH approved institutional plan, this study will be audited approximately every 6 months.

7.10 Endpoint Data

7.10.1 Endpoint data will be collected using HITC IML data forms, participant-specific binders, and the HITC laboratory database.

7.10.2 The HITC laboratory database, which has password-restricted access, is stored on the UVA Health System Computing Services secure server.

8.0 EVALUATION OF IMMUNOLOGIC RESULTS

A primary goal is to evaluate immunogenicity of LPV7 in each of 7 adjuvant preparations, including IFA, with or without TLR agonists. The primary immunological response of interest is the CD8+ T cell response to the defined minimal peptides epitopes contained within each of the 7 long peptides (Table 2). This will be assessed primarily by ELIspot assay as outlined below. Associated analyses will include the durable immune response (week 26), and the peak magnitude of the CD8+ T cell responses. Details related to study design are in Section 9.

Studies in the peripheral blood will also assess CD4 T cell responses, T cell persistence, antibody response, Th1/Tc1 deviation and cytokine functionality. The antitumor immune response to vaccination with LPV7 with TLR agonists will be compared to vaccination with

IFA alone, and the highest immune response rate will be selected. Blood will be collected as described in Section 6.

Skin biopsy specimens from the vaccine sites after 1 (day 8) and 3 vaccines (day 22) (and from normal skin controls) will be evaluated for changes induced by TLR agonists, and these will be evaluated as we have for a prior study [50]: immunohistochemistry of the vaccine-site immune cells to define the cell types and function; multiparameter flow cytometry to identify activation status of antigen specific cells, regulatory T cells, and MDSC; ELIspot assays for the ability to produce IFNγ; and Luminex studies to define the cytokine and chemokine profiles of the VSME.

The following laboratory criteria will be used to evaluate effects of the vaccines and the TLR agonists in circulating peripheral blood mononuclear cells (PBMC) and in the vaccine sites. In general, immunological evaluations will be performed in batch.

8.1 Immunohistochemistry and Immunofluorescence of skin biopsy specimens

For the immunohistochemical studies of the VSME, we will employ stains with single antibodies (eg CD8), with multiple antibodies (eg CD8 and FoxP3), and a technique developed by our colleague Jim Mandell, which we have working our lab now. This technique, called SIMPLE, enables repeated IHC stains on the same section, with whole-slide imaging using an Aperio or Leica system, and multiparameter evaluation of these cells in situ by combining the multiple images with each antibody.[67] Immunofluorescence studies will also be available in the event they are helpful.

We will obtain CD8/FoxP3 ratios to evaluate whether higher (more favorable) ratios are induced by adjuvants that are associated with improved circulating immune responses.

For optimization of antibodies in IHC, we have used positive control tissues, which will continue to be useful controls in the proposed studies. These include, but are not limited to, normal human peripheral blood lymphocytes (PBL), PBL activated with CD3/CD28 beads (activated PBL), human lymph node tissue from non-melanoma patients (LN), and inflamed human tissue from a surgical specimen of Crohn's disease (Crohn's).

8.2 ELIspot assay

We will evaluate CD4+ and CD8+ T cell responses in the peripheral blood using a direct (ex vivo) ELIspot assay for IFNγ as reported.[12] This will be performed in a core laboratory dedicated to immune monitoring, and with SOPs, controls, and quality assurance measures, which we have reported.[12]. Vaccination site infiltrating lymphocytes will be evaluated by ELIspot as well. Vaccine site infiltrating lymphocytes will be compared to those in peripheral blood. ELIspot assays after one in vitro stimulation (stimulated ELIspot) may also be employed if appropriate, as reported [33].

CD4+ T cell responses will be assessed by IFNγ ELIspot assay using the mixture of 7 long peptides and also pools of 15-mer peptides (overlapping by 5 residues) from each peptide (4 peptides for each long peptide) as antigen sources.

Durability of CD8 and helper T cell responses will be assessed by measuring T cell activity at 26 weeks, and for longer intervals when available, with the hypothesis that there will be increased durability of T cell responses when compared to prior experience with short peptides[13] and will be improved with TLR agonists when compared to IFA alone in this study.

We have extensive stores of cryopreserved T cells reactive to minimal epitope peptides included in the 12 short peptides (12MP) mixture, and detectable by IFN-gamma ELIspot assay [8;12] and by cytotoxicity assays.[68;69]. These cells will serve as positive controls for reactivity to the minimal epitopes contained within the long peptides used in this trial.

8.3 Tetramer assay and multiparameter flow cytometry
Peripheral blood lymphocytes and VSME lymphocytes will be evaluated by flow cytometry after incubation with MHC-peptide tetramers or pentamers for the number of peptide-reactive T-cells. We have validated tetramers for all 5 of the minimal CD8 epitopes restricted by HLA-A2 or A3 among the LPV7 peptides. We will use these MHC multimers with which we also have substantial experience.[12] Tetramers will likely detect more antigen-specific T cells than are detected in the functional ELIspot assay, but the primary measure of CD8 T cell response will be the ELIspot assay, on which the statistical power was based. Tetramers will also be used in multiparameter flow cytometry to determine the effector/memory status of the antigen-specific T cells [70] (CD45RA, CD27, CD28, CCR7, CD62L), and evaluation for multiple functions of the CD8 T cells (degranulation (CD107a), IFNγ, MIP-1β, TNFα, and IL-2) [71]

We will also use multiparameter flow cytometry to measure myeloid suppressor cells (CD14+ HLA-DR lo/neg), and several subtypes of MDSC in PBMC to determine if they are increased or decreased by any of these vaccine approaches, compared to our current experience with MDSC numbers using our short 12MP vaccine (data not shown).

8.4 Proliferation assay / Cytokine Analysis
To characterize responding T cells more completely, we will evaluate with multiparameter flow cytometry after short-term culture with antigen. $CD4^+$ T cells responding to antigen by CFSE dilution will be assayed for intracellular cytokine production to evaluate Th1/Th2/Th17 bias (Th1: IL-2, IFNγ, TNFα; Th2: IL4, IL-5, IL-10; Th17: IL-17a). Antigen specific T-regs will also be assayed as described [72].

We will also assay supernatants of PBMC, and of immune cells infiltrating the VSME, cultured 2 and 5 days with the 7 long peptides to define secreted cytokines and chemokines representative of the whole cell population. We anticipate that the long peptides will induce Tc1 and Th1 dominant responses, with a high proportion of memory phenotype after several vaccines.

Peripheral blood mononuclear cells (PBMC) and vaccine-site inflammatory cells from participants in this clinical trial may also be evaluated for CD4 T cell reactivity, using a 3H-thymidine uptake assay, CFSE dilution or other assays as summarized above.

Responses to the tetanus helper peptide will be assayed as well as responses to the 30-mer melanoma peptides and 15-mer pools comprising those peptides. Either or both of two peptides will be used as negative controls for $CD4^+$ T cell responses: one being the PADRE peptide (aK(X)VAAWTLKAa), where X = L-cyclohexylalanine [73;74].

8.5 Antibody responses
Antibody responses to 30 mer peptides (and to pools of 15-mer peptides) will be measured by ELISA assay using methods described by Sabbatini et al [2].

8.6 Evaluation of tumor

Tumor tissue collected prior to enrollment or at the time of progression will be evaluated by routine histology and immunohistochemistry. In addition, *in vitro* evaluations of tumor tissue and tumor infiltrating lymphocytes may be completed.

9.0 STATISTICAL CONSIDERATIONS

This is an open-label, Phase I/II study evaluating the safety and immunogenicity of a long peptide vaccine (LPV7) + TLR for resected stage IIB – IV melanoma. There are two parts to the study.

For Part 1, the trial was designed to find the range of optimal combination therapies, defined as combinations with high immunologic response and an acceptable level of toxicity. The primary outcomes included the frequency of treatment related dose limiting toxicities (DLTs) and frequency of immunologic response. For Part 2, the goal is to obtain preliminary safety and immunogenicity data on the optimal combination identified in Part 1 when administered in the same skin site for all 6 vaccines (same site vaccination).

For part 2, the vaccine regimen is selected based on available data from Part 1, in which the strongest immune responses were observed in participants enrolled on Arms E and G (Table 1A). Resiquimod is no longer available; so the regimen for Arm G is not currently feasible. Also, the total enrollment on Arm E is larger (15 vs 6) so that outcomes from Part 2 will be evaluable in a similar sample size as for Part 1, Arm E. This group will be called Study Arm E2.

9.1 Study design Part 1 (completed)

In order to assess the first two objectives, the **co-primary objective** is to determine a range of optimal combination therapies, among the combinations provided in the following table where an optimal combination is a combination that is estimated to have an acceptable toxicity profile as measured by DLT's and a high rate of immunologic response (IRR) as measured by peak immune response.

Table 14: Study Design

Zone	Arm/Combination	Peptide vaccine	Adjuvant preparation
1	A	LPV7 + tet	IFA
1	B	LPV7 + tet	PolyICLC
1	C	LPV7 + tet	Resiquimod
2	D	LPV7 + tet	PolyICLC + Resiquimod
2	E	LPV7 + tet	IFA + PolyICLC
2	F	LPV7 + tet	IFA + Resiquimod
3	G	LPV7 + tet	IFA + PolyICLC + Resiquimod

DLTs are defined in Section 7.8 of the protocol.

Within the range of optimal doses a **co-primary objective** is if more than one combination is contained within the range of optimal combinations, to estimate the difference in immunologic response rates between combinations, among those in the range of optimal combinations.

9.2 Combination allocation Part 1 (completed)

The goal is to determine the range of optimal combinations, where optimal combination allocation will occur in two stages. The initial stage will accrue eligible participants in cohorts of one on each arm per the allocation process in 9.2.1 until a participant experiences a DLT. The second stage will allocate eligible participants based upon a continual reassessment method (CRM)[81] for combinations of agents[82]. Randomization will be based on equal allocation among allowable arms unless a weighted allocation scheme is triggered (Section 9.2.1). Randomization will not be stratified by institution. Participants must be observed for a minimum of 3 weeks after the initial vaccine for initial escalation between zones, and between the 1st and 2nd participants within an arm.

9.2.1 Combination allocation in Stage 1:

The initial stage will accrue eligible participants on combinations in cohorts of one until a participant experiences a dose-limiting toxicity (DLT). The escalation plan for the first stage is based on grouping treatment combinations into “zones.” With this dose escalation design participants can be accrued and assigned to other open combinations within a zone but escalation will not occur outside the zone until the minimum follow-up period is observed for the first participant accrued to a combination. Allocation will begin in Zone 1.

- Allocation in Zone 1:
  a) The first eligible participant in Zone 1 will be entered onto combination A, B or C, chosen at random.
  b) The second eligible participant in Zone 1 will be randomized to one of the two remaining combinations in the zone that have not yet been tried.
  c) The third eligible participant will be entered onto the combination in Zone 1 that has not yet been tried.
  d) Escalation to a Zone 2 occurs only when all combinations in Zone 1 have accrued at least 1 participant and no participants have experienced a DLT.

- Allocation in Zone 2:
  a) After completion of Zone 1, the first eligible participant in Zone 2 will be entered onto combination D, E or F, chosen at random.
  b) The second eligible participant in Zone 2 will be randomized to one of the two remaining combinations in the zone that have not yet been tried.
  c) The third eligible participant will be entered onto the combination in Zone 2 that has not yet been tried.
  d) Escalation to a Zone 3 occurs only when all combinations in Zone 2 have accrued at least 1 participant and no participants have experienced a DLT.

- Allocation in Zone 3:
  a) Note Zone 3 contains a single combination, so the first eligible participant on Zone 3 will be entered onto combination G.
  b) If 0/1 participants experience a DLT, the next eligible participant will be entered onto combination G. If 0/2 participants experience DLT, the next eligible participant will be randomized to any combination.
  c) In the absence of DLT’s, participants will continue to be randomized to combination A through G until a DLT occurs (upon which the modeling stage begins), or until at least 3 participants have been treated at every combination, at which point allocation will be based on which combination has the highest IRR. In the absence of DLT’s, participants will continue to be entered on the combination with the highest IRR until sufficient information has been obtained regarding the

optimal combination, according to the stopping rules described below. Once a DLT has been observed, Stage 2 using CRM modeling begins.

9.2.2 Combination allocation in Stage 2:

The second stage will allocate eligible participants based upon a continual reassessment method for combinations of agents (R package, pocrm). The modeling stage uses (a) a selected set of possible orderings for the DLT probabilities and (b) a working model for the DLT probabilities under each ordering.

Table 15: Possible Orderings of DLT Probabilities

Possible orderings of DLT probabilities
1) A-B-C-D-E-F-G
2) A-C-B-D-F-E-G
3) B-A-C-E-D-F-G
4) B-C-A-E-F-D-G
5) C-A-B-F-D-E-G
6) C-B-A-F-E-D-G

Table 16: Working Model of DLT Probabilities Under Each Ordering

Working model of DLT probabilities under each ordering**							
Order	A	B	C	D	E	F	G
1	0.01	0.05	0.12	0.20	0.28	0.36	0.45
2	0.01	0.12	0.05	0.20	0.36	0.28	0.45
3	0.05	0.01	0.12	0.28	0.20	0.36	0.45
4	0.12	0.01	0.05	0.36	0.20	0.28	0.45
5	0.05	0.12	0.01	0.28	0.36	0.20	0.45
6	0.12	0.05	0.01	0.36	0.28	0.20	0.45
**Working models were chosen according to the algorithm of Lee and Cheung (2009)							

Within each ordering, the continual reassessment method (CRM) is fit, using the working model and the accumulated data. The second stage will accrue eligible participants in cohorts of one and use a CRM model fit to estimate DLT probabilities at each arm combination. For each order, $m = 1, \cdots, 6,$ in Table 16, the DLT probabilities for each arm combination $i = 1, \cdots, 7,$ are modeled via a one-parameter power model, $Pr(\text{DLT at combination } i) \approx p_{mi}^{\theta_m}$, where the p_{mi} are the working model values for order m given in Table 16. After accrual of each participant into the trial, the parameter θ_m is estimated for each ordering by maximum likelihood estimation where the likelihood is given by $\prod_{i=1}^{7} \left(p_{mi}^{\theta_m}\right)^{y_i} \left(1 - p_{mi}^{\theta_m}\right)^{n_i - y_i}$ where y_i= the number of DLTs and n_i=the number of treated participants in arm combination i. The order with the largest likelihood is chosen and, within this ordering, DLT probability estimates are updated for each combination. If there is a tie between the likelihood values of two or more orderings, then the selected order is randomly chosen from among the tied orderings. These DLT probabilities will be used to define a set of “acceptable” combinations, defined as any dose with estimated DLT probability less than or equal to 33%. This toxicity tolerance of 33% was chosen based on the expectedness of adverse events.

9.2.3 Combination recommendation:

Once the set of "acceptable" combinations is determined, the recommended combination will be based upon how many participants have been entered into the study to that point. If less than 3 participants have been treated on any acceptable dose, then the recommended combination for the next entered participant will be chosen at random from the acceptable combinations. If at least 3 participants have been treated at every acceptable combination, the recommended combination for the next participant entered is defined as the "acceptable" combination with the highest observed IRR. After each participant, a new recommended combination is obtained, and the next entered participant is allocated to the recommended combination. Part 1 of the trial will stop once sufficient information about the optimal dose range has been obtained, according to the stopping rules outlined in Section 9.6.1.

9.3 Statistical properties Part 1 (completed)

Simulation results were run (R package, pocrm) to display the performance of the design characteristics. For each scenario, 1000 simulated trials were run. Each table reports the true DLT probability at each combination, the true IRR at each combination, the percentage of trials in which each combination was recommended as the optimal combination, and the average number of participants treated in a simulated study.

9.3.1 Scenario 1:

All true DLT probabilities are safe (i.e. less toxic than 33%) and one combination has the highest IRR. The true optimal combination is indicated in italicized bold type.

Table 17: Study Scenario 1

Zones	1			2			**3**
Combination	A	B	C	D	E	F	***G***
True DLT probabilities	1%	2%	5%	7%	11%	14%	***20%***
True IRR	35%	35%	40%	55%	60%	65%	***80%***
% optimal combination recommendation	0%	1%	1%	7%	10%	18%	***62%***
% participant allocation	8%	8%	9%	12%	14%	17%	***33%***
Average trial size: 45 participants (25th, 50th, 75th percentile = 40, 43, 48)<br>0.4% of simulated trials were stopped for safety<br>The overall toxicity rate was 11.8%. The overall IRR was 60.9%.							

9.3.2 Scenario 2:

One combination (G) has true DLT probability more toxic than 33% and the three combinations in Zone 2 have the highest IRR among safe combinations. The true range of optimal combinations is indicated in italicized bold type.

Table 18: Study Scenario 2

Zones	1			2			3
Combination	A	B	C	***D***	***E***	***F***	G
True DLT probabilities	5%	6%	8%	***22%***	***23%***	***25%***	45%
True IRR	45%	50%	50%	***65%***	***65%***	***65%***	80%
% optimal combination recommendation	6%	10%	10%	***25%***	***22%***	***20%***	5%
% participant allocation	12%	14%	13%	***18%***	***18%***	***17%***	8%
Average trial size: 45 participants (25th, 50th, 75th percentile = 39, 44, 52)							

2.2% of simulated trials were stopped for safety<br>The overall toxicity rate was 18.3%. The overall IRR was 60.0%.

9.3.3 Scenario 3:
Combinations in Zones 2 and 3 have true DLT probabilities more toxic than 33% and the two combinations in Zone 1 have the highest IRR among safe combinations. The true range of optimal combinations is indicated in italicized bold type.

Table 19: Study Scenario 3

Zones	1			2			3
Combination	***A***	***B***	***C***	D	E	F	G
True DLT probabilities	***18%***	***20%***	***25%***	45%	48%	54%	64%
True IRR	***65%***	***70%***	***70%***	80%	80%	80%	90%
% optimal combination recommendation	***19%***	***28%***	***25%***	1%	1%	0%	0%
% participant allocation	***25%***	***29%***	***29%***	6%	6%	5%	1%
Average trial size: 29 participants (25th, 50th, 75th percentile = 21, 33, 39)<br>25.2% of simulated trials were stopped for safety<br>The overall toxicity rate was 25.4%. The overall IRR was 70.6%.							

9.3.4 Scenario 4:
All true DLT probabilities are unsafe (i.e. more toxic than 33%).

Table 20: Study Scenario 4

Zones	1			2			3
Combination	A	B	C	D	E	F	G
True DLT probabilities	50%	55%	56%	65%	66%	74%	80%
True IRR	70%	70%	70%	80%	80%	80%	90%
% optimal combination recommendation	1%	1%	3%	0%	0%	0%	0%
% participant allocation	32%	28%	35%	2%	2%	1%	0%
Average trial size: 7 participants (25th, 50th, 75th percentile = 2, 3, 6)<br>96% of simulated trials were stopped for safety<br>The overall toxicity rate was 45.6%. The overall IRR was 70.3%.							

9.4 Study design Part 2
A fixed sample size design will be used in part 2. The vaccine regimen is selected based on available data from Part 1, in which the recommended combination was Arm E. For Part 2, all accruals will be to Study Arm E2.

9.5 Sample size/accrual

9.5.1 Part 1 (completed)
Maximum sample size is based upon acquiring sufficient information to assess the objective of selecting the arm with the highest IRR, assuming at least one optimal combination has been found.

Data from our prior Mel43 trial in the arms not getting GM-CSF [12] indicated a high T cell response (5-fold increase in reactivity, at least 1200 cells per 100,000 CD8, and no overlap

of 2 SD) to the 12 peptide vaccine in PBMC in 30 of 60 participants (50%, 95%CI (37%, 63%)). Using the range as an approximate guideline, maximum sample size is established to ensure that if IRR among all seven arms differ by 30%, with the smallest expected rate of 50%, then the arm with the higher IRR would be selected with high probability, P=0.90. The initial maximum target accrual of 20 eligible participants per arm is based upon the ranking and selection procedure, B_{SH} [75], which allows selection of the best treatment worthy of further investigation in single-factor Bernoulli response experiments. However, it is not anticipated that all arms will fall in the range of optimal combinations, thus, sample size is estimated from the simulations and will be determined by the stopping rules below. Although maximum accrual is set at 140, in the simulation results below, on average a total of between 7 and 46 participants were required to complete the study.

Accrual is estimated at 2 participants per month at each site (4 per month overall). The maximum accrual to the study is estimated from the simulations to be approximately 52 participants. Adjusting for 10% ineligibility/dropout/withdrawal rate, maximum total target accrual for Part 1 is estimated at 58 participants.

9.5.2 Part 2

Immune response was observed in 6/16 (37.5%, 90%CI [17.8, 60.9%]) from Part 1. For Part 2 target accrual is set at 16 eligible participants. At study conclusion, if we observe that at least 12/16 (a doubling of the response rate) eligible participants satisfy the definition of immune response then the lower limit of a one-sided 80%CI around the observed immune response rate for Arm E2 would be 61.5% which we would conclude supports that the immune response rate is likely to be higher for single site injections. If fewer responses are observed then additional studies will be needed to better understand the impact of site rotation with the use of the tetanus helper peptide.

Accrual is estimated at 2 participants per month. Adjusting for 10% dropout rate, maximum total target accrual for Part 2 is estimated at 18 participants.

9.6 <u>Stopping rules:</u>

9.6.1 Part 1 (completed)

The study will be stopped according to the following rules:

1. The study will stop early for safety if the first three entered participants in Zone 1 experience DLT on combinations in Zone 1 in Stage 1.
2. If at any point in Stage 2, the set of acceptable combinations is empty, the trial will stop for safety.
3. The study will stop if the recommendation is to assign the next participant to a combination that already has 20 participants treated at that combination.

9.6.2 Part 2

Safety will be assessed by monitoring the number of participants who experience a DLT. In Part 1, 1/16 (6.3%, 95%CI [0.2, 30.2%]) participants treated on Arm E experienced a DLT. For Part 2 the upper boundary of a sequential probability ratio test (SPRT) based upon a binomial test of proportions for DLT will be used for monitoring to protect against excessive DLT rates. The stopping boundary is for a SPRT contrasting a 5% versus 30% DLT rate, with nominal type I and II errors of 5% and 20%, respectively. Safety stopping guidelines for the occurrence of DLTs are detailed in the following table. If a stopping bound is crossed then accrual to the study will be suspended until the study PI, co-investigators and the DSMC can review the data, and determine if the study should continue, be amended or be closed to further accrual.

Table 21: Incidence of Treatment-Related DLTs

Incidence of treatment related DLTs	
Number of participants	Stopping boundary
2-4	≥ 2
5-11	≥ 3
12-18	≥ 4

9.7 Analyses

9.7.1 Safety:

All participants who receive any study intervention will be monitored for adverse events. Adverse events will be described and coded based upon the NCI CTCAE v4.03. A DLT is defined as any unexpected adverse event that is possibly, probably or definitely related to treatment and satisfies the criteria in section 7.8. Occurrence of DLTs will guide escalation and stopping decisions. At study conclusion frequency, proportion and severity of adverse events, and DLTs by arm will be tabulated.

9.7.2 Immunologic response:

Subjects are evaluable for immune response if at least one post treatment sample is measurable for response. Peak immunogenicity will be evaluated by direct ELIspot assay against the defined nonamer peptides in the peripheral blood. The presence or absence of an immune response will be defined by induction of at least a 2-fold increase in IFN-gamma-secreting cells over background, and over pre-existing responses, and at least 30 IFN-gamma secreting spots per 100,000 CD8 T cells, as described [34]. This assessment will be performed on blood through week 12, although a minimum of 4 weeks of data will be used to guide decisions about the range of optimal dose combinations. IRR is defined as the proportion of participants where presence (as defined above) of an immune response has been observed.

Durable immunogenicity will be evaluated week 26 by ELIspot assay against the defined nonamer peptides in the peripheral blood. The presence or absence of an immune response at that time will be defined by induction of at least a 2-fold increase in IFN-gamma-secreting cells over background, and over pre-existing responses, and at least 30 IFN-gamma secreting spots per 100,000 CD8 T cells, as described [34]. This assessment will be performed on blood through week 26.

9.7.3 At study conclusion:

Frequency and magnitude of adverse events will be summarized by arm. Point estimates and confidence intervals will be calculated for all dichotomous endpoints. If more than one combination is contained within the range of optimal combinations, estimates and confidence intervals will be used to estimate the difference in IRR between pairs of combinations among those in the range of optimal combinations.

Immunogenicity will be evaluated by direct ELIspot assay against the defined nonamer peptides in vaccine site biopsies using the above criteria. Disease-free survival is defined as the duration of time from start of treatment to time of recurrence/progression or death from any cause, whichever occurs first. Participants who do not experience an event (recurrence/progression or death) will be censored at date of last contact. Overall survival is defined as the duration of time from start of treatment to time of death from any cause. Participants who do not experience an event (death) will be censored at date of last contact.

Disease-free survival and overall survival distributions will be estimated by the product limit method of Kaplan and Meier.

10.0 Appendices

PRC# 1143-10 / IRB-HSR#15931 / IND# 15891
Version Date: 07-31-17

10.1 Appendix 1: X-page

Studies & Tests			Active Treatment									Follow-up		
	Day	Pre	1	8	15	22	36	43	57	78	85	183	365	730
	Week		0	1	2	3	5	6	8	11	12	26	52	104
	Vax #		1	2	3		4		5	6				
Informed consent [a]		X												
Class I HLA-typing [a]		X												
Pathology review [a]		X												
β-HCG [c]		X												
HIV / Hepatitis C [d]		X												
HGBA1C [b]		X												
Urinalysis [b]		X												
CBC with differential [b]		X				X				X	X			
Comprehensive chemistry [b, f]		X[e]				X				X	X			
LDH [b]		X				X				X	X			
Anti-nuclear antibody / Rf factor			X			X				X		X		
Chest x-ray / CT [b, g]		X									X	X		
Abdominal CT [b, g]		X												
Pelvic CT [b, g]		X												
Head MRI / CT [b, g]		X												
History & physical [b]		X	X	X	X	X	X	X	X	X	X	X	X	X
120cc green top tubes			X											
80cc green top tubes				X		X		X			X	X	X	X
20cc red top tubes			X	X		X		X			X	X	X	X
Vaccination			X	X	X		X		X	X				
Biopsy normal skin, pts 1-6, in Part 1				X[i]		X[i]								
Vaccine Site Biopsies				X		X								
Visual acuity, and color acuity examinations			X								X			
Note hair and eye color			X								X			
Note vitiligo			X			X					X	X		
Note neurologic function			X			X					X	X		
Distribute/review participant toxicity diary			X	X	X	X	X	X	X	X	X[h]			

[a] Any point prior to randomization
[b] Pre-study within 6 weeks of randomization
[c] Within 2 weeks of randomization (for childbearing women)
[d] Within 6 months of randomization
[e] To include fasting glucose. Fasting blood sugars may be evaluated 4h or more after last eating.
[f] Comprehensive chemistry panel to include sodium, potassium, creatinine, glucose, calcium, total bilirubin, AST, ALT, and alkaline phosphatase
[g] PET-CT may replace CT of the chest, abdomen, and/or pelvis. New abnormalities of AST, ALT, or LDH levels, or new nodules on CXR will warrant more complete staging with CT scans of the chest, abdomen and pelvis or PET-CT and with CT or MRI of the brain as appropriate.
[h] Review only
[i] Biopsies of normal skin to be done just for the first 6 patients enrolled in Part 1.

10.2 Appendix 2: AJCC Staging System, 7th Edition

Melanoma TNM Classification

T Classification	Thickness	Ulceration Status
T1	≤ 1.0 mm	a: without ulceration and level II/III
		b: with ulceration or mitoses ≥1/mm2
T2	1.01 – 2.0 mm	a: without ulceration
		b: with ulceration
T3	2.01 – 4.0 mm	a: without ulceration
		b: with ulceration
T4	> 4.0 mm	a: without ulceration
		b: with ulceration

N Classification	# of Metastatic Nodes	Nodal Metastatic Mass
N1	1 node	a: micrometastasis*
		b: macrometastasis†
N2	2 – 3 nodes	a: micrometastasis*
		b: macrometastasis†
		c: in transit met(s)/satellite(s) without metastatic nodes
N3	4 or more metastatic nodes, or matted nodes, or in transit met(s)/satellites(s) with metastatic node(s)	

M Classification	Site	Serum Lactate Dehydrogenase
M1a	Distant skin, subcutaneous or nodal mets	Normal
M1b	Lung metastases	Normal
M1c	All other visceral metastases	Normal
	Any distant metastasis	Elevated

* Micrometastases are diagnosed after sentinel or elective lymphadenectomy.

† Macrometastases are defined as clinically detectable nodal metastases confirmed by therapeutic lymphadenectomy or when nodal metastasis exhibits gross extracapsular extension.

PRC# 1143-10 / IRB-HSR#15931 / IND# 15891
Version Date: 07-31-17

Stage Groupings for Cutaneous Melanoma

	Clinical Staging			Pathologic Staging		
	**T**	**N**	**M**	**T**	**N**	**M**
**0**	Tis	N0	M0	Tis	N0	M0
**IA**	T1a	N0	M0	T1a	N0	M0
**IB**	T1b<br>T2a	N0<br>N0	M0<br>M0	T1b<br>T2a	N0<br>N0	M0<br>M0
**IIA**	T2b<br>T3a	N0<br>N0	M0<br>M0	T2b<br>T3a	N0<br>N0	M0<br>M0
**IIB**	T3b<br>T4a	N0<br>N0	M0<br>M0	T3b<br>T4a	N0<br>N0	M0<br>M0
**IIC**	T4b	N0	M0	T4b	N0	M0
**III‡**	Any T	N1<br>N2<br>N3	M0			
**IIIA**				T1-4a<br>T1-4a	N1a<br>N2a	M0<br>M0
**IIIB**				T1-4b<br>T1-4b<br>T1-4a<br>T1-4a<br>T1-4a/b	N1a<br>N2a<br>N1b<br>N2b<br>N2c	M0<br>M0<br>M0<br>M0<br>M0
**IIIC**				T1-4b<br>T1-4b<br>Any T	N1b<br>N2b<br>N3	M0<br>M0<br>M0
**IV**	Any T	Any N	Any M1	Any T	Any N	Any M1

* Clinical staging includes microstaging of the primary melanoma and clinical/radiologic evaluation for metastases. By convention, it should be used after complete excision of the primary melanoma with clinical assessment for regional and distant metastases.

† Pathologic staging includes microstaging of the primary melanoma and pathologic information about the regional lymph nodes after partial or complete lymphadenectomy. Pathology stage 0 or stage 1A patients are the exception; they do not require pathologic evaluation of their lymph nodes.

‡ There are no stage III subgroups for clinical staging.

<u>Addendum: Staging for Mucosal Melanomas</u>

This system is based on the staging of cutaneous melanomas.

Stage IIB: Clinically localized primary melanoma > 4mm thick
Stage III: Lymph node metastases
Stage IV: Distant metastases

Patel SP, *et al*. *J Immunother Cancer* 2021; 9:e003220. doi: 10.1136/jitc-2021-003220

10.3 Appendix 3: ECOG Performance Status

Grade	Performance
0	Fully active, able to carry on all pre-disease performance without restriction
1	Restricted in physically strenuous activity but ambulatory and able to carry out work of a light or sedentary nature, e.g. light house work, office work
2	Ambulatory and capable of all self-care, but unable to carry out any work activities. Up and about more than 50% of waking hours
3	Capable of only limited self-care, confined to bed or chair more than 50% of waking hours
4	Completely disabled. Cannot carry on any self-care. Totally confined to bed or chair
5	Dead

* As published in Am. J. Clin. Oncol.: Oken, M.M., Creech, R.H., Tormey, D.C., Horton, J., Davis, T.E., McFadden, E.T., Carbone, P.P.: Toxicity And Response Criteria Of The Eastern Cooperative Oncology Group. Am J Clin Oncol 5:649-655, 1982.

10.4 Appendix 4: New York Heart Association Disease Classification

**Functional Capacity**	**Objective Assessment**
**Class I.** Patients with cardiac disease but without resulting limitation of physical activity. Ordinary physical activity does not cause undue fatigue, palpitation, dyspnea, or anginal pain.	No objective evidence of cardiovascular disease.
**Class II.** Patients with cardiac disease resulting in slight limitation of physical activity. They are comfortable at rest. Ordinary physical activity results in fatigue, palpitation, dyspnea, or anginal pain.	Objective evidence of minimal cardiovascular disease
**Class III.** Patients with cardiac disease resulting in marked limitation of physical activity. They are comfortable at rest. Less than ordinary activity causes fatigue, palpitation, dyspnea, or anginal pain.	Objective evidence of moderately severe cardiovascular disease.
**Class IV.** Patients with cardiac disease resulting in inability to carry on any physical activity without discomfort. Symptoms of heart failure or the anginal syndrome may be present even at rest. If any physical activity is undertaken, discomfort is increased.	Objective evidence of severe cardiovascular disease.

* The Criteria Committee of the New York Heart Association. Nomenclature and Criteria for Diagnosis of Diseases of the Heart and Great Vessels. 9th ed. Boston, Mass: Little, Brown & Co; 1994:253-256

10.5 Appendix 5: Lot Testing

A. Preparation of the synthetic melanoma and tetanus peptides

All peptides have been synthesized under GMP conditions by Polypeptide Group (San Diego, CA) or comparable provider. Certificates of analysis and technical summaries for each of the peptides will be included in the chemistry and manufacturing portion of the IND application.

Peptide preparation and vialing has been performed under GMP conditions by Clinalfa/Merck for the tetanus peptide, and Bachem for the NY-ESO-1 long peptide, and will be performed under GLP conditions by the UVA Human Immune Therapy Center laboratory for the 6 peptides in the LPV6 long peptide mixture. Documentation relating to the procedures used to prepare and vial the peptides will comply with the CFR and will be included in the Chemistry and Manufacturing Section of the IND application.

B. Lot Release Quality Assurance Testing

Prepared peptides will be subjected to the following tests:

1. Identity: Identity will be confirmed by structural studies. The individual peptides will be tested for identity by mass spectrometry (to define molecular mass and amino acid sequence) and high performance liquid chromatography (HPLC, to confirm purity) in a GMP laboratory (Polypeptide Group). Amino acid analysis will also be performed to confirm identity further.

   Before mixing, the amino acid sequence of each individual peptide preparation will be reconfirmed by mass spectrometry or co-elution.

   After combining the peptides, the mixture will be subjected to HPLC analysis.

2. Purity: Purity will be assessed before and after vialing the peptide mixtures. Before vialing the peptide mixtures, each synthetic peptide will be evaluated for the presence of a single dominant species by high performance liquid chromatography (HPLC). Purity of each peptide component will exceed 90% (expected 94%-98%). Variants of the original peptide may include incomplete products of synthesis, minor degradation products due to oxidation of methionine residues, and dimerization of cysteine-containing peptides. After vialing the peptide mixture, purity will be reconfirmed by HPLC.

3. Aggregation: Aggregation of peptides will be assessed by HPLC. Aggregation is not expected.

4. Trifluoroacetic acid (TFA): The amount of total fluorine in each peptide preparation will be less than 0.5% or 5000 ppm.

5. Potency: The net peptide content will be calculated for each synthesis. The amounts of each peptide (mcg quantities) added to the vaccine vials are calculated based on the net peptide content of the original stock of lyophilized peptides.
   The amount of peptide in each sterile filtered peptide solution will be measured by amino acid analysis. The final vials will also be tested for quantity of total peptide by amino acid analysis. Lot release criteria are defined in the Chemistry and Manufacturing section of the IND application.

6. Endotoxin: Endotoxin testing will be conducted in accordance with CFR guidelines.

7. General Safety: General safety testing will be conducted in accordance with US CFR guidelines.

8. Sterility: Sterility testing will be conducted in accordance with CFR guidelines.

C. Ongoing Quality Assurance Testing

Stability: We will follow guidelines for quality assurance studies to assure stability of the peptide preparation over time during the trial[76]. The peptide preparations will be assayed for stability every six months for 2 years and every year thereafter. The following analyses will be performed to confirm stability:

1. HPLC: HPLC will be performed to confirm purity. A comparison to previous HPLC data will be performed. Ideally, the purity of each peptide component will exceed 90% (94%-98%). Variants of the original peptide may include incomplete products of synthesis, minor degradation products due to oxidation of methionine residues, and dimerization of cysteine-containing peptides. Such minor variants will be tolerated as long as the intended peptide represents at least 75% of the total peptide species. With liquid formulations of this peptide preparation, we have observed dimerization of cysteine-containing peptides, yet this peptide retained immunogenicity even when as little as 10% remains as monomer. For the present study, dimerization of cysteine-containing peptides will be acceptable as long as at least 25% remains as monomer by HPLC. Because measures of peptide quantity are subject to variability, a peptide lot will be rejected only if two sequential measures fail to meet the criterion stated above. If a second measure is required, the lot will be held until the second reading is obtained and shown to be acceptable.

2. Aggregation. Aggregation will also be assessed by HPLC.

3. Mass spectrometry. This will be performed on each peptide peak to define the molecular sequence and mass if there is any ambiguity about the identity of the peptides based on HPLC. New peaks on HPLC, or significant change in the elution profiles will warrant determination of the peptide identities by mass spectrometry on each peak.

4. Sterility: One vial of peptide will be submitted to the Clinical Microbiology Laboratory at the University of Virginia or Microbiology Research Associates, Inc. (Acton, MA). Sterility testing will be conducted in accord with FDA guidelines in 21 CFR part 610.12.

10.6 Appendix 6: NCI Common Terminology Criteria for Adverse Events v4.03

http://evs.nci.nih.gov/ftp1/CTCAE

10.7 Appendix 7: Summary of Changes

07-31-17	1) Updated Table of Contents.<br>2) Section 1.1: updated to address current treatments for resected melanoma in the adjuvant setting.<br>3) Section 1.2.6: updated to address ipilimumab approval for treatment of resected melanoma in the adjuvant setting.<br>4) Section 4.1: removed reference to interferon packet.<br>5) Section 5.6.1: added targeted molecular therapy (e.g. vemurafenib, other inhibitor of mutant BRAF, MEK, or cKit) as nonmerpitted treatments.<br>6) Section 5.6.2.6: edited to indicate that any live vaccines (e.g. FluMist) are permitted, but should be administered at least 2 weeks prior to or at least 2 weeks after a study vaccine.<br>7) Updated reference list.
06-26-17	1) Updated cover sheet<br>2) Updated study personnel<br>3) Updated formatting and numbering throughout study document<br>4) Additional administrative changes and typographical errors corrected throughout the document to improve readability.<br>5) Table of Contents: Updated<br>6) Protocol precis<br>• Design: The study has been divided into Part 1 and Part 2. Part 1 required rotating vaccine sites and accrual to this part was completed. Part 2 of the study has been added and the regimen requires that each vaccine is administered in the same skin site for all 6 vaccines.<br>• The objectives have been revised to add objectives specific for part 2.<br>• Table 1 has been re-labeled as Table 1A. The title of the table has been revised to specify that Table 1A is relevant for Part 1 of the study.<br>• Addition of Table 1B: Study Arm E2 has been specified for Part 2 of the study.<br>• The rationale for choosing the vaccine regimen from study arm E has been added.<br>• Regimen: Revised to describe regimen for Parts 1 and 2 of the study.<br>• Vaccine site biopsies: revised to specify that 3 punch biopsies of normal skin will be completed in the first 6 patients enrolled in Part 1 of the study.<br>• Leadership: Revised to specify that Part 2 of the study will be conducted just at the University of Virgina.<br>• Accrual: Adjusted to allow for an additional patients to be enrolled to part 2 of the study.<br>7) Section 1.0: Updated accrual information.<br>8) Section 1.1: Updated information describing incidence of melanoma and deaths from melanoma in 2017 and added a section on vaccine site selection to provide a rationale for evaluating same site vaccinations.<br>9) Section 1.2.6: Updated information on approved adjuvant therapies for melanoma.<br>10) Section 1.2.10: Added prior experience with same site vaccinations.<br>11) Added Table 5 to describe vaccine site reactions for prior Mel44 and Mel58 studies.<br>12) Section 2.0: Revised objectives to add objectives for Part 2.

	13) Section 3.1: Regimen-added the regimen for Part 2, which includes same site vaccinations.<br>14) Section 4.2.7(1): Corrected section reference.<br>15) Section 4.4.2: Revised randomization section and specified that Part 2 of the study will not include randomization.<br>16) Section 5.2.1: Added a description for preparing drug for Study Arm E2.<br>17) Section 5.4.2: Deleted prior text describing the vaccine regimen and replaced with a description for the administration of vaccines for Part 1 and Part 2 and a description of expected vaccine site toxicities.<br>18) Section 5.4.3: A section describing doses for part 2 of the study has been added.<br>19) Section 5.4.6 , 6.2.2, 6.2.4, Appendix 1: Clarified that biopsies of normal skin will be completed in the first 6 participants who are enrolled in Part 1.<br>20) Section 5.6: Clarified that patients will be randomized in Part 1 and assigned to an arm in Part 2 of the study.<br>21) Section 7.4.3: Clarified that grade 3 injection site reactions with significant ulceration of the skin (> 2 cm) will be considered DLTs. Clarified dermatologic toxicities for resiquimod.<br>22) Section 7.8: Added grade 3 injection site reaction with ulceration ≤ 2 cm as an exception to the DLT rule<br>23) Section 9.0: Revised introduction to statistical considerations to add information about Part 2 of the study.<br>24) Section 9.1-removed the DLT rule from this section and revised to refer to section 7.8 of the protocol.<br>25) Sections 9.2, 9.3, 9.4, 9.5, 9.6: clarified which sections pertain to Part 1 and added statistical language for Part 2.<br>26) Reformatted headings for the appendices<br>27) Appendix 7: added a summary of changes list for prior versions of the protocol
06-17-14	1) Sapna Patel's role was changed from co-investigator to PI at MD Anderson Cancer Center.<br>2) Table of Contents page numbers were updated.
06-06-14	1) Section 1.2.3: Editorial change for clarification<br>2) Section 1.2.4: Editorial change for clarification<br>3) Section 1.2.5: The dosing for resiquimod was revised to include an acceptable dose range of 400-600 mg. Instructions about coverage and washing the affected area have been incorporated into the study protocol.<br>4) Section 1.2.8: Reference added<br>5) Table 3: Reformatted<br>6) Table 4: Updated with new data<br>7) Section 4.1.4: Clarification of inclusion criterion and whether repeat scans are needed after stereotactic radiotherapy.<br>8) Section 4.1.5: Clarification of inclusion criterion and the description of required lymph node basins.<br>9) Section 4.1.8: Clarification of inclusion criterion a) specifications for patients with Gilbert's disease and b) re-defined acceptable limit for Hgb-A1C level.<br>10) Section 4.2.11: Addition of the following to allowable autoimmune disorders: Hypothyroidism of any etiology on stable thyroid hormone replacement therapy.<br>11) Section 5.2.1: Arms E and G: Change in mixing instructions for PolyICLC. PolyICLC will be incorporated into the vaccine mixture rather than administered as a separate injection.<br>12) Section 5.2.1: Clarification of vaccine components for Study Arms C, D, F and G. The dose of resiquimod was adjusted from 500 mg to approximately 500 mg. Instructions aoubt coverage and washing the affected area have

	been incorporated into the study protocol.<br>13) Reference List: Updated.
03-25-14	1) Dr. Geoffrey Weiss and Dr. Elizabeth Gaughan have been added to the list of co-investigators.<br>2) The Table of Contents was updated.<br>3) The dose of resiquimod has been corrected throughout the study document. The correct dose is 1000 mcg and the drug will be supplied in multi-dose tubes. Section 1.2.6 also includes information on prior usage with this dose.<br>4) Section 4.1.4: An editorial change was made and also a clarification that the brain metastases in this section refer to treated brain metastases.<br>5) Section 4.1.8: The lab parameters inclusion criteria were reformatted. HIV and HCV requirements were moved to the exclusion criteria (Section 4.2.6).<br>6) Section 4.2.3: The reference to Section 4.2.3 within this section was corrected to Section 4.2.4.<br>7) Section 4.4.1: Information about the interferon education packets was changed to include a statement that participants mus t convice the study team that the answered the questions accurately or understand the correct answers.<br>8) Section 5.4.1: A clarification was made to this section to specify that chronic inflammation is expected in subjects who receive Montanide ISA-51. Also, editorial changes were made to this section.<br>9) Section 6.2.2: “Vaccine site biopsy (3)” was removed from the Day 8 schedule of events as the biopsy procedures were listed twice for this day.<br>10) Sections 6.2.1, 6.2.2, 6.2.3, 6.2.4, 6.2.5, 6.2.6, 6.2.7, 6.2.8: Clarfication was made to the language pertaining to the interval history and physical exam. Examination of vaccine sites was added to each of the study visit days, when applicable.<br>11) Section 7: Editorial correction was made to change “or” to “for”.<br>12) Section 7.4.3: A redundant section related to AEs that do not require expedited reporting. Reporting guidelines are provided in Table 12.<br>13) Section 7.4.3: A clarification was made to the dosing regimen that was used for resiquimod in prior studies.<br>14) Section 7.6.1: A clarification was made to the process of reporting AEs. AEs determined to require expedited reporting to the sponsor, must also be reported to a site’s institution according to local policy and procedures.<br>15) Section 7.6.3: A clarification was made regarding reporting AEs to the sites. Serious and unexpected suspected adverse reactions will be reported to the sites no later than 15 calendar days after the Sponsor determines that the the requirements for an IND safety report have been met.<br>16) Section 7.7.2: The phrase “and at UVA by the UVA melanoma team” was removed from this section as it did not apply to the outside sites. Individual AEs will be reviewed at each site.<br>17) Section 7.7.3: The term “weekly” was removed when describing the melanoma team meetings. The melanoma team meets 20 times a year, as specified in section 7.7.4 of the DSMP. The term “may” was included in the description of the events that are reviewed at each meeting as the agenda varies depending upon study accruals.<br>18) Section 7.7.4 : The DSMP was modified to specify that month conference calls will be held between the Sponsor and the sites.<br>19) Section 9.2.2: “R-package, pocrm” was added for clarification. Additional statistical methods were added to clarify the model of DLT probabilities.

	20) 9.6.3: The endpoints of disease-free survival and overall survival have been defined.<br>21) Appendix 1: editorial change made to subscript "i".<br>22) Appendix 5: Clarifications were made to the descriptions of the peptide preparation and vialing.
10-10-13	1) Cover sheet has been updated<br>2. New study staff members have been added and the formatting for contact information has been corrected.<br>3. Zone information has been added to the précis (Table 1) and Table 5 and the description of the regimen has been modified to reflect changes in the statistical section.<br>4. Table of Contents has been updated.<br>5. List of abbreviations has been updated.<br>6. The timing of the negative control biopsies has been clarified throughout the protocol. The negative control biopsies will be completed on days 8 and 22 in the first 6 patients.<br>7. Table 3: An explanation of the grading criteria are provided.<br>8. Section 1.2.6 and 5.2.1: additional directions for applying the resiquimod have been added.<br>9. Section 4.4.1: Replaced the section that participants will have to complete the interferon packet questions satisfactorily with a statement that answers to questions in the packet will be reviewed with the participant.<br>10. Section 4.4.2: A description of when randomization is to take place in relation to registration and the start of treatment has been added.<br>11. Section 4.4.3: "suture removal" was removed from the evalution following the biopsies as punch biopsies will not require sutures.<br>12. Section 5.2 and 5.4.3: The dosage of and preparation of the vaccines were clarified.<br>13. Section 5.4.6.4: Information on specimen collection delays and the associated documentation are provided.<br>14. Section 5.6.2.6: clarification made to permitted influenza vaccines<br>15. Section 6.2 Treatment: The schedules for the immunologic bloods, vaccines, and biopsies have been moved to this section.<br>16. Section 7.0 Regulatory and Reporting Requirements: This entire section has been revised and reformatted to clarify the reporting requirments for the study. The original reporting tables have been replaced with a template table from the UVA Cancer Center DSMP and an IRB reporting table has been added. In addition, reporting criteria for reporting to the FDA have been replaced.<br>17. Section 7.1.3: A clarification for the definition of hospitalization for expedited AE reporting has been added.<br>18. Section 7.5: Adverse event classifications have been updated and the hospitalization category has been moved to the description for an SAE.<br>19. Section 7.5: AE classifications have been updated to include Hematologic/Metabolic and Non-Hematologic/Non-metabolic categories. Autoimmune has been added to the allergic category.<br>20. Section 7.7.1: Replaced section stating that someone from the research team would be available 24 hours a day 7 days a week for questions with the statement that subjects would be given information on how to contact their providers if they have questions or problems.<br>21. Section 9.1: The table has been modified to correct the description of the adjuvant preparation for each arm/combination.<br>22. Section 9.2: A description of the randomization has been added.

	23. Appendix 1: Administrative changes have been made to the X-page to improve readability.<br>24. Additional administrative changes and typographical errors corrected throughout the document to improve readability.
08-06-13	1) An additional study group has been added in response to the recommendations of the FDA and of our collaborators at MD Anderson Cancer Cente<br>2) The study design has been changed to incorporate an adaptive design in order to minimize data with the least number of patients. This reduces the target sample size by half.<br>3) Section 4.4 of the study protocol has been modified to include additional details regarding the registration and randomization of study patients.<br>4) A section for study drug accountability and distribution has been added (section 5.3)<br>5) Table of Contents has been updated.<br>6) The source of Hiltonol (polyICLC) has been updated, in section 1.2.5.<br>7) Updated section 1.2.10 to specify that the peptides for the vaccines have been synthesized under GMP conditions, rather than GLP.<br>8) Appendices about vaccine preparation, immunologic assays, and details of tissue handling with the biopsies has been removed from the protocol and will be included ina Study Manual and Investigator Brochure.<br>9) Numerous small administrative changes have been made to improve clarity, consistency and readability and to provide appropriate citations.<br>10) Details about the quality assurance studies to be done on the peptides have been added to respond to the FDA after our preIND meeting.<br>11) Skin biopsies have been amended: Patients 1-6 will have 3 skin punch biopsies rather than 2, on day 1. The 3rd sample from skin on day 1 in those patients and from all patients on days 8 and 22 will be stored in RNA later rather than be collected for viable cell studies.<br>12) Signature page and study staff contact information has been added.<br>13) X-page has been corrected with regards to the timing of the normal skin biopsies.
12/05/11	1) Section 4.4 of the study protocol has been modified to include additional details regarding the registration and randomization of study patients.<br>2) A section for study drug accountability and distribution has been added (section 5.3)<br>3) Table of Contents has been updated.

11.0 Reference List

(1) Kenter GG, Welters MJ, Valentijn AR et al. Vaccination against HPV-16 oncoproteins for vulvar intraepithelial neoplasia. *N Engl J Med* 2009;361:1838-1847.

(2) Sabbatini P, Tsuji T, Ferran L et al. Phase I Trial of Overlapping Long Peptides from a Tumor Self-Antigen and Poly-ICLC Shows Rapid Induction of Integrated Immune Response in Ovarian Cancer Patients. *Clin Cancer Res* 2012;18:6497-6508.

(3) Gnjatic S, Sawhney NB, Bhardwaj N. Toll-like receptor agonists: are they good adjuvants? *Cancer J* 2010;16:382-391.

(4) Speiser DE, Lienard D, Rufer N et al. Rapid and strong human CD8+ T cell responses to vaccination with peptide, IFA, and CpG oligodeoxynucleotide 7909. *J Clin Invest* 2005;115:739-746.

(5) Schwartzentruber DJ, Lawson DH, Richards JM et al. gp100 peptide vaccine and interleukin-2 in patients with advanced melanoma. *N Engl J Med* 2011;364:2119-2127.

(6) Ascierto PA, Gogas HJ, Grob JJ et al. Adjuvant interferon alfa in malignant melanoma: an interdisciplinary and multinational expert review. *Crit Rev Oncol Hematol* 2013;85:149-161.

(7) Agarwala SS, Lee SJ, Flaherty LE et al. Randomized phase III trial of high-dose interferon alfa-2b (HDI) for 4 weeks induction only in patients with intermediate- and high-risk melanoma (Intergroup trial E 1697) . [abstract]Agarwala SS, Lee SJ, Flaherty LE et al. *J Clin Oncol* 2011;29 suppl:Abstract 8505

(8) Slingluff CL, Jr., Petroni GR, Chianese-Bullock KA et al. Immunologic and clinical outcomes of a randomized phase II trial of two multipeptide vaccines for melanoma in the adjuvant setting. *Clin Cancer Res* 2007;13:6386-6395.

(9) Slingluff CL, Jr., Chianese-Bullock KA, Bullock TN et al. Immunity to melanoma antigens: from self-tolerance to immunotherapy. *Adv Immunol* 2006;90:243-95.:243-295.

(10) Kirkwood JM, Lee S, Land S et al. E1696: Final analysis of the clinical and immunological results of a multicenter ECOG phase II trial of multi-epitope peptide vaccination for stage IV melanoma with MART-1 (27-35), gp100 (209-217, 210M), and tyrosinase (368-376, 370D) (MGT) +/- IFNa2b and GM-CSF. [abstract]Kirkwood JM, Lee S, Land S et al. *J Clin Oncol (Meeting Abstracts)* 2004;22:7502

(11) Rosenberg SA, Sherry RM, Morton KE et al. Tumor progression can occur despite the induction of very high levels of self/tumor antigen-specific CD8+ T cells in patients with melanoma. *J I* 2005;175:6169-6176.

(12) Slingluff CL, Jr., Petroni GR, Olson WC et al. Effect of GM-CSF on circulating $CD8^+$ and $CD4^+$ T cell responses to a multipeptide melanoma vaccine: Outcome of a multicenter randomized trial. *Clin Cancer Res* 2009;15:7036-7044.

(13) Slingluff CL, Jr., Petroni GR, Yamshchikov GV et al. Immunologic and clinical outcomes of vaccination with a multiepitope melanoma peptide vaccine plus low-dose interleukin-2 administered either concurrently or on a delayed schedule. *J Clin Oncol* 2004;22:4474-4485.

(14) Slingluff CL, Jr., Petroni GR, Olson W et al. Helper T cell responses and clinical activity of a melanoma vaccine with multiple peptides from MAGE and melanocytic differentiation antigens. *J Clin Oncol* 2008;26:4973-4980.

(15) Rosenberg SA, Yang JC, Restifo NP. Cancer immunotherapy: moving beyond current vaccines. *Nat Med* 2004;10:909-915.

(16) Rosenberg SA, Yang JC, Kammula US et al. Different adjuvanticity of incomplete freund's adjuvant derived from beef or vegetable components in melanoma patients immunized with a peptide vaccine. *J Immunother* 2010;33:626-629.

(17) Slingluff CL, Jr., Petroni GR, Smolkin ME et al. Immunogenicity for $CD8^+$ and $CD4^+$ T cells of two formulations of an incomplete Freund's adjuvant for multipeptide melanoma vaccines. *Journal of Immunotherapy* 2010;33:630-638.

(18) Hailemichael Y, Dail Z, Jaffarzad N et al. Persistent antigen at vaccination sites induces tumor-specific CD8+ T cell sequestration, dysfunction and deletion. *J Exp Med* 2013;in press.

(19) Salerno EP, Shea SM, Olson WC et al. Activation, dysfunction and retention of T cells in vaccine sites after injection of incomplete Freund's adjuvant, with or without peptide. *Cancer Immunol Immunother* 2013.

(20) Lawson DH, Lee SJ, Tarhini AA, Margolin KA, Ernstoff MS, Kirkwood JM. E4697: Phase III cooperative group study of yeast-derived granulocyte macrophage colony-stimulating factor (GM-CSF) versus placebo as adjuvant treatment of patients with completely resected stage III-IV melanoma. [abstract]Lawson DH, Lee SJ, Tarhini AA, Margolin KA, Ernstoff MS, Kirkwood JM. *J Clin Oncol* 2010;28:abstract 8504

(21) Song XT, Turnis ME, Zhou X et al. A Th1-inducing adenoviral vaccine for boosting adoptively transferred T cells. *Mol Ther* 2011;19:211-217.

(22) Ly LV, Sluijter M, Versluis M et al. Peptide vaccination after T-cell transfer causes massive clonal expansion, tumor eradication, and manageable cytokine storm. *Cancer Res* 2010;70:8339-8346.

(23) Song S, Zhang K, You H et al. Significant anti-tumour activity of adoptively transferred T cells elicited by intratumoral dendritic cell vaccine injection through enhancing the ratio of CD8(+) T cell/regulatory T cells in tumour. *Clin Exp Immunol* 2010;162:75-83.

(24) Lou Y, Wang G, Lizee G et al. Dendritic cells strongly boost the antitumor activity of adoptively transferred T cells in vivo. *Cancer Res* 2004;64:6783-6790.

(25) Pilon-Thomas S, Mackay A, Vohra N, Mule JJ. Blockade of programmed death ligand 1 enhances the therapeutic efficacy of combination immunotherapy against melanoma. *J Immunol* 2010;184:3442-3449.

(26) Rosenberg SA. A new era for cancer immunotherapy based on the genes that encode cancer antigens. [Review] [79 refs]. *Immunity* 1999;10:281-287.

(27) Cox AL, Skipper J, Chen Y et al. Identification of a peptide recognized by five melanoma-specific human cytotoxic T cell lines. *Science* 1994;264:716-719.

(28) Skipper JC, Kittlesen DJ, Hendrickson RC et al. Shared epitopes for HLA-A3-restricted melanoma-reactive human CTL include a naturally processed epitope from Pmel-17/gp100. *JI* 1996;157:5027-5033.

(29) Salgaller ML, Marincola FM, Cormier JN, Rosenberg SA. Immunization against epitopes in the human melanoma antigen gp100 following patient immunization with synthetic peptides. *Cancer Res* 1996;56:4749-4757.

(30) Parkhurst MR, Salgaller ML, Southwood S et al. Improved induction of melanoma-reactive CTL with peptides from the melanoma antigen gp100 modified at HLA-A*0201-binding residues. *J Immunol* 1996;157:2539-2548.

(31) Slingluff CL, Jr., Petroni GR, Yamshchikov GV et al. Clinical and immunologic results of a randomized phase II trial of vaccination using four melanoma peptides either administered in granulocyte-macrophage colony-stimulating factor in adjuvant or pulsed on dendritic cells. *J Clin Oncol* 2003;21:4016-4026.

(32) Slingluff CL, Jr., Petroni GR, Yamshchikov GV et al. Immunologic and clinical outcomes of vaccination with a multiepitope melanoma peptide vaccine plus low-dose interleukin-2 administered either concurrently or on a delayed schedule. *J Clin Oncol* 2004;22:4474-4485.

(33) Slingluff CL, Jr., Petroni GR, Chianese-Bullock KA et al. Immunologic and Clinical Outcomes of a Randomized Phase II Trial of Two Multipeptide Vaccines for Melanoma in the Adjuvant Setting. *Clin Cancer Res* 2007;13:6386-6395.

(34) Slingluff CL, Jr., Petroni GR, Chianese-Bullock KA et al. A randomized multicenter trial of the effects of melanoma-associated helper peptides and cyclophosphamide on the immunogenicity of a multipeptide melanoma vaccine. *J Clin Oncol* 2011;29:2924-2932.

(35) Kawakami Y, Eliyahu S, Jennings C et al. Recognition of multiple epitopes in the human melanoma antigen gp100 by tumor-infiltrating T lymphocytes associated with in vivo tumor regression. *J Immunol* 1995;154:3961-8.

(36) Kittlesen DJ, Thompson LW, Gulden PH et al. Human melanoma patients recognize an HLA-A1-restricted CTL epitope from tyrosinase containing two cysteine residues: implications for tumor vaccine development. *J Immunol* 1998;160:2099-2106.

(37) Yamshchikov GV, Barnd DL, Eastman S et al. Evaluation of peptide vaccine immunogenicity in draining lymph nodes and peripheral blood of melanoma patients. *Int J Cancer* 2001;92:703-711.

(38) Skipper JC, Hendrickson RC, Gulden PH et al. An HLA-A2-restricted tyrosinase antigen on melanoma cells results from posttranslational modification and suggests a novel pathway for processing of membrane proteins. *J Exp Med* 1996;183:527-534.

(39) Chaux P, Luiten R, Demotte N et al. Identification of five MAGE-A1 epitopes recognized by cytolytic T lymphocytes obtained by in vitro stimulation with dendritic cells transduced with MAGE-A1. *J Immunol* 1999;163:2928-2936.

(40) Slingluff C, Petroni G, Chianese-Bullock K et al. Effects of melanoma-derived helper peptides and cyclophosphamide on the immunogenicity of a multipeptide melanoma vaccine [abstract]Slingluff C, Petroni G, Chianese-Bullock K et al. *Journal of Immunotherapy* 2009;32:976

(41) Huang LQ, Brasseur F, Serrano A et al. Cytolytic T lymphocytes recognize an antigen encoded by MAGE-A10 on a human melanoma. *J Immunol* 1999;162:6849-6854.

(42) Valmori D. Use of HLA-A2/peptide tetramers for the analysis of CD8+ T-cell responses to CT antigens in melanoma patients [abstract]Valmori D. *Cancer Vaccines Meeting (October 2-4, 2000), sponsored by the Cancer Research Institute* 2000;S12

(43) Karbach J, Gnjatic S, Bender A et al. Tumor-reactive CD8+ T-cell responses after vaccination with NY-ESO-1 peptide, CpG 7909 and Montanide ISA-51: association with survival. *Int J Cancer* 2010;126:909-918.

(44) Nicholaou T, Ebert LM, Davis ID et al. Regulatory T-cell-mediated attenuation of T-cell responses to the NY-ESO-1 ISCOMATRIX vaccine in patients with advanced malignant melanoma. *Clin Cancer Res* 2009;15:2166-2173.

(45) Gnjatic S, Altorki NK, Tang DN et al. NY-ESO-1 DNA vaccine induces T-cell responses that are suppressed by regulatory T cells. *Clin Cancer Res* 2009;15:2130-2139.

(46) Bioley G, Guillaume P, Luescher I et al. Vaccination with a recombinant protein encoding the tumor-specific antigen NY-ESO-1 elicits an A2/157-165-specific CTL repertoire structurally distinct and of reduced tumor reactivity than that elicited by spontaneous immune responses to NY-ESO-1-expressing Tumors. *J Immunother* 2009;32:161-168.

(47) Scalzo AA, Elliott SL, Cox J, Gardner J, Moss DJ, Suhrbier A. Induction of protective cytotoxic T cells to murine cytomegalovirus by using a nonapeptide and a human-compatible adjuvant (Montanide ISA 720). *Journal of Virology* 1995;69:1306-1309.

(48) Fernandez IM, Snijders A, Benaissa-Trouw BJ, Harmsen M, Snippe H, Kraaijeveld CA. Influence of epitope polarity and adjuvants on the immunogenicity and efficacy

of a synthetic peptide vaccine against Semliki Forest virus. *Journal of Virology* 1993;67:5843-8.

(49) Ahlers JD, Dunlop N, Alling DW, Nara PI, Berzofsky JA. Cytokine-in-adjuvant steering of the immune response phenotype to HIV-1 vaccine constructs: granulocyte-macrophage colony-stimulating factor and TNF-alpha synergize with IL-12 to enhance induction of cytotoxic T lymphocytes. *J Immunol* 1997;158:3947-58.

(50) Schaefer JT, Patterson JW, Deacon DH et al. Dynamic changes in cellular infiltrates with repeated cutaneous vaccination: A histologic and immunophenotypic analysis. *Journal of Translational Medicine* 2010;8:79.

(51) Lore K, Betts MR, Brenchley JM et al. Toll-like receptor ligands modulate dendritic cells to augment cytomegalovirus- and HIV-1-specific T cell responses. *J Immunol* 2003;171:4320-4328.

(52) Perrot I, Deauvieau F, Massacrier C et al. TLR3 and Rig-like receptor on myeloid dendritic cells and Rig-like receptor on human NK cells are both mandatory for production of IFN-gamma in response to double-stranded RNA. *J Immunol* 2010;185:2080-2088.

(53) Levy H, Salazar A. Interferon inducers. In: Baron S, Copenhaver D, Dianzani F, et al., eds. *Interferon: Principles and Medical Applications*. Galveston, TX: Galveston University, University of Texas Medical Branch; 1992;65-76.

(54) Peng G, Guo Z, Kiniwa Y et al. Toll-like receptor 8-mediated reversal of CD4+ regulatory T cell function. *Science* 2005;309:1380-1384.

(55) Spruance SL, Tyring SK, Smith MH, Meng TC. Application of a topical immune response modifier, resiquimod gel, to modify the recurrence rate of recurrent genital herpes: a pilot study. *J Infect Dis* 2001;184:196-200.

(56) Pockros PJ, Guyader D, Patton H et al. Oral resiquimod in chronic HCV infection: safety and efficacy in 2 placebo-controlled, double-blind phase IIa studies. *J Hepatol* 2007;47:174-182.

(57) Kirkwood JM, Strawderman MH, Ernstoff MS, Smith TJ, Borden EC, Blum RH. Interferon alfa-2b adjuvant therapy of high-risk resected cutaneous melanoma: the Eastern Cooperative Oncology Group Trial EST 1684 [see comments]. *J Clin Oncol* 1996;14:7-17.

(58) Kirkwood JM, Ibrahim JG, Sondak VK et al. High- and low-dose interferon alfa-2b in high-risk melanoma: first analysis of intergroup trial E1690/S9111/C9190. *J Clin Oncol* 2000;18:2444-2458.

(59) Kirkwood JM, Ibrahim JG, Sosman JA et al. High-dose interferon alfa-2b significantly prolongs relapse-free and overall survival compared with the GM2-KLH/QS-21 vaccine in patients with resected stage IIB-III melanoma: results of intergroup trial E1694/S9512/C509801. *J Clin Oncol* 2001;19:2370-2380.

(60) Kawakami Y. Immunobiology of human melanoma antigens MART-1 and gp100 and their use for Immuno-gene therapy. *Int Rev Immunol* 1997;142:173-192.

(61) Dudley ME, Wunderlich JR, Robbins PF et al. Cancer regression and autoimmunity in patients after clonal repopulation with antitumor lymphocytes. *Science* 2002;298:850-854.

(62) Judge JM, Chianese-Bullock KA, Schroen AT, Slingluff CL, Jr. Usefulness of prestudy assessment of patient willingness to undergo tissue biopsy for correlative studies in a melanoma vaccine trial. *Clin Trials* 2013;10:143-150.

(63) Balch CM, Gershenwald JE, Soong SJ et al. Final version of 2009 AJCC melanoma staging and classification. *J Clin Oncol* 2009;27:6199-6206.

(64) Cochran AJ, Morton DL, Stern S, Lana AM, Essner R, Wen DR. Sentinel lymph nodes show profound downregulation of antigen-presenting cells of the paracortex: implications for tumor biology and treatment. *Mod Pathol* 2001;14:604-608.

(65) Szeimies RM, Bichel J, Ortonne JP, Stockfleth E, Lee J, Meng TC. A phase II dose-ranging study of topical resiquimod to treat actinic keratosis. *Br J Dermatol* 2008;159:205-210.

(66) Tam RC, Hong Z, Moran M, Varnavski A, Kim SK. Therapeutic strategies targeting the innnate antiviral immune responses (section heading: TLR agonists as therapeutic IFN inducers). In: Wu-Pong S, Rojanasakul Y, eds. *Biopharmaceutical Drug Design and Development*. 2nd ed. Humana Press; 2008;241.

(67) Glass G, Papin JA, Mandell JW. SIMPLE: a sequential immunoperoxidase labeling and erasing method. *Journal of Histochemistry & Cytochemistry* 2009;57:899-905.

(68) Chianese-Bullock KA, Pressley J, Garbee C et al. MAGE-A1-, MAGE-A10-, and gp100-derived peptides are immunogenic when combined with granulocyte-macrophage colony-stimulating factor and montanide ISA-51 adjuvant and administered as part of a multipeptide vaccine for melanoma. *JI* 2005;174:3080-3086.

(69) Yamshchikov GV, Barnd DL, Eastham S et al. Evaluation of peptide vaccine immunogenicity in draining lymph nodes and blood of melanoma patients. *Int J Cancer* 2001;92:703-711.

(70) Sallusto F, Lenig D, Forster R, Lipp M, Lanzavecchia A. Two subsets of memory T lymphocytes with distinct homing potentials and effector functions. *Nature* 1999;401:708-712.

(71) Betts MR, Nason MC, West SM et al. HIV nonprogressors preferentially maintain highly functional HIV-specific CD8+ T cells. *Blood* 2006;107:4781-4789.

(72) Welters MJ, Kenter GG, de Vos van Steenwijk PJ et al. Success or failure of vaccination for HPV16-positive vulvar lesions correlates with kinetics and phenotype of induced T-cell responses. *Proc Natl Acad Sci U S A* 2010;107:11895-11899.

(73) Alexander J, Sidney J, Southwood S et al. Development of high potency universal DR-restricted helper epitopes by modification of high affinity DR-blocking peptides. *Immunity* 1994;1:751-761.

(74) Del Guercio MF, Alexander J, Kubo RT et al. Potent immunogenic short linear peptide constructs composed of B cell epitopes and Pan DR T helper epitopes (PADRE) for antibody responses in vivo. *Vaccine* 1997;15:441-448.

(75) Bechhofer RESTJaGDM. *Design and analysis of experiments for statistical selection, screening, and multiple comparisons*. New York: John Wiley & Sons, 1995.

(76) Chianese-Bullock KA, Lewis ST, Sherman NE, Shannon JD, Slingluff CL, Jr. Multi-peptide vaccines vialed as peptide mixtures can be stable reagents for use in peptide-based immune therapies. *Vaccine* 2009;27:1764-1770.

(77) Slingluff CL, Jr., Petroni GR, Olson WC, Smolkin ME, Ross MI, Haas NB, Grosh WW, Boisvert ME, Kirkwood JM, Chianese-Bullock KA. Effect of granulocyte/macrophage colony-stimulating factor on circulating CD8+ and CD4+ T-cell responses to a multipeptide melanoma vaccine: outcome of a multicenter randomized trial. Clini Cancer Res 2009; 15: 7036-7044.

(78) Mauldin IS, Wages NA, Stowman AM, Wang E, Olson WC, Deacon DH, Smith KT, Galeassi N, Teague JE, Smolkin ME, Chianese-Bullock KA, Clark RA, Petroni GR, Marincola FM, Mullins DW, Slingluff CL, Jr. Topical treatment of melanoma metastases with imiquimod, plus administration of a cancer vaccine, promotes immune signatrues in the metastases. Cancer Immunol Immunother 2016; 65: 1201-1212.

(79) Mauldin IA, Wages NA, Stowman AM, Wang E, Smolkin ME, Olson WC, Deacon DH, Smith KT, Galeassi NV, Chianese-Bullock KA, Dengel LT, Marincola FM, Petroni GR, Mullins DW, Slingluff CL, Jr. Intratumoral interferon-gamma increases chemokine production but fails to increase T cell infiltration of human melanoma metastases. Cancer Immunol Immunother 2016; 65: 1189-1199.

(80) Siegel, RL, Miller, KD, Jemal, A. Cancer Statistics. CA: A Cancer Journal for Clinicians 2017; 67: 7-30.

(81) O'Quigley, J, Pepe M, Fisher L. Continual reassessment method: a practical design for phase 1 clinical trials in cancer. Biometrics 1990; 46: 33-48.

(82) Wages NA, Conaway MR, O'Quigley, J. Continual reassessment method for partioal ordering. Biometrics 2011; 67: 1555-1563.

(83) Higano, C. S., P. F. Schellhammmer, E. J. Small, P. A. Burch, Nemunaitis J, L. Yuh, N. Provost, and M. W. Frohlich. Integrated data from 2 randomized, double-blind, placebo-controlled, phase 3 trials of active cellular immunotherapy with sipuleucel-T in advanced prostate cancer. *Cancer* 115: 3670-3679.

(84) Eggermont AMM, Chiarion-Sileni V, Grob JJ, et al. Adjuvant ipilimumab versus placebo after complete resection of high-risk stage III melanoma (EORTC 18071): a randomised, double-blind, phase 3 trial. Lancet Oncol 2015; 16: 522–30

(85) Tarhini A, Lee S, Kirwood J, et al. A phase III randomized study of adjuvant ipilimumab (3 or 10 mg/kg) versus high-dose interferon alfa-2b for resected high-risk melanoma (U.S. Intergroup E1609): Preliminary safety and efficacy of the ipilimumab arms. Abstract. J Clin Oncol 2017; 35.
