# Supplementary material for: Phase I/II trial of a long peptide vaccine (LPV7) plus toll-like receptor (TLR) agonists with or without incomplete Freund’s adjuvant (IFA) for resected high-risk melanoma
Source: J Immunother Cancer. 2021 Aug 19;9(8):e003220. doi: 10.1136/jitc-2021-003220 (PMC8378357; doi:10.1136/jitc-2021-003220)
Supplement: Supplementary data [file jitc-2021-003220supp002.pdf]

Supplemental Table 1. Clinical Trial Study Arms and Treatments

Zone	Study Arm	Peptide vaccine	Adjuvant	Dose of TLR agonist	Route vax injection	Admin of TLR agonist
1	A	LPV7 + tet	IFA	---	Id/sq	--
1	B	LPV7 + tet	PolyICLC	1 mg	Id/sq	Id/sq
1	C	LPV7 + tet	Resiquimod	1000 mcg	Id/sq	Topical
2	D	LPV7 + tet	PolyICLC + resiquimod	1 mg/ 1000 mcg	Id/sq	Id/sq (PolyICLC) & topical (resiquimod)
2	E	LPV7 + tet	IFA + PolyICLC	1 mg	Id/sq	Id/sq
2	F	LPV7 + tet	IFA + resiquimod	1000 mcg	Id/sq	Topical
3	G	LPV7 + tet	IFA + PolyICLC + resiquimod	1 mg/ 1000 mcg	Id/sq	Id/sq (PolyICLC) & topical (resiquimod)

Supplemental Table 2. Patient demographics

	Arm A IFA	Arm B pICLC	Arm C Resiq	Arm D pICLC + Resiq	Arm E IFA + pICLC +	Arm B. IFA + Resiq	Arm G IFA + pICLC + Resiq	Overall
N	5	7	4	6	16	6	6	50
Age on study (years)								
Mean	48	57	62	49	54	55	60	55
Median	52	64	64	53	60	56	59	58
Gender								
F	2	3	2	1	9	0	3	20
M	3	4	2	5	7	6	3	30
Race								
White	5	7	4	6	15	6	6	49
American Indian or Alaska Native	0	0	0	0	1	0	0	1
Ethnicity								
Hispanic	0	0	0	0	0	0	0	0
Institution								
Institution 1 (UVA)	4	5	3	5	7	3	4	31
Institution 2 (MDACC)	1	2	1	1	9	3	2	19
ECOG PS at registration								
0	4	7	3	6	14	6	5	45
1	1	0	1	0	2	0	1	5
HLA*								
A1+	3	5	2	2	7	.	2	21
A2+	3	4	.	3	5	4	1	20
A3+	3	1	2	2	7	2	4	21
B35+	2	1	.	1	2	3	1	10
B51+	.	1	1	.	.	.	.	2
Primary site								
Skin, non-acral	3	7	3	5	15	5	6	44
Unknown	2	.	1	1	1	1	.	6
Stage at Registration*								
IIB-IIC	0	1	0	0	0	1	1	3
III	5	5	4	3	14	5	5	41
III, NOS	0	0	0	0	1	0	1	2
IIIA	1	1	2	1	5	0	1	11
IIIB/C	4	4	2	2	8	5	3	28
IV	0	1	0	3	2	0	0	6

Supplemental Table 3. Treatment-related adverse events:

MEL 60 Maximum Grade Toxicities (Related)		Arms																								% with this AE
		N=5 Arm A IFA			N=7 Arm B PolyICLC			N=4 Arm C Resiquimod			N=6 Arm D PolyICLC + resiquimod			N=16 Arm E IFA + PolyICLC			N=6 Arm F IFA + resiquimod			N=6 Arm G IFA + PolyICLC + resiquimod			N=50 ----- Total			
Toxicity Category	Toxicity Description	G1	G2	G3	G1	G2	G3	G1	G2	G3	G1	G2	G3	G1	G2	G3	G1	G2	G3	G1	G2	G3	G1	G2	G3	
Participant Highest Grade	*Any*	1	4		3	4		2	1		4	2		6	9	1	2	4		1	5		19	29	1	98%
BLOOD/LYMPHATIC	OTHER													1									1			2%
EAR/LABYRINTH	TINNITUS	1																					1			2%
GASTROINTESTINAL	CONSTIPATION													1			1						2			4%
	DIARRHEA		1		1												2			1			4	1		10%
	MUCOSITIS ORAL													1									1			2%
	NAUSEA		1					1			2			5			1	1		2			11	2		26%
	OTHER																			1			1			2%
	VOMITING													1			1						2			4%
GENERAL AND ADMINISTRATION SITE	CHILLS	1			2						3			10	2		3			2	1		21	3		48%
	FATIGUE	2	2		5						2	1		8	4		2	1		4			23	8		62%
	FEVER				1						2			6	2		1	1		2			12	3		30%
	FLU LIKE SYMPTOMS				1						3			1			1			2			8			16%
	INJECTION SITE REACTION	2	3		2	4			1		4	2		8	6	1	3	3		3	3		22	22	1	90%
	LOCALIZED EDEMA													1						1			2			4%
	OTHER	1			2									6			3			1			13			26%
	PAIN										1			1			1			2			5			10%
IMMUNE SYSTEM	AUTOIMMUNE DISORDER													1									1			2%
INJURY/POISONING/PROCEDURAL	BRUISING													1									1			2%
INVESTIGATIONS	LYMPHOCYTE COUNT DECREASED																				1			1		2%
	WEIGHT LOSS																1						1			2%
	WHITE BLOOD CELL DECREASED													1	1								1	1		4%
METABOLISM/NUTRITION	ANOREXIA	1												2			1						4			8%
MUSCULOSKELETAL/CONNECTIVE TISSUE	ARTHRALGIA	1			2						1			6						4			14			28%
	GENERALIZED MUSCLE WEAKNESS													1									1			2%
	MYALGIA	3			2						2			6	1		1						14	1		30%
	OTHER													1									1			2%
	PAIN IN EXTREMITY		1		1						1			1									3	1		8%
NERVOUS SYSTEM	DIZZINESS	1									2			2						1			6			12%
	DYSGEUSIA													1									1			2%
	HEADACHE	2			2						3			6			1			1			15			30%
PSYCHIATRIC	ANXIETY																1						1			2%
RESPIRATORY/THORACIC/MEDIASTINAL	ALLERGIC RHINITIS	1			1									2			1						5			10%
	COUGH				1									1						1	1		3	1		8%
	DYSPNEA										1												1			2%
	SORE THROAT				1									1			1			2			5			10%
SKIN/SUBCUTANEOUS TISSUE	HYPERHIDROSIS				1									7			1			1			10			20%
	PAIN OF SKIN																			1			1			2%
	PRURITUS	1															1						2			4%
	RASH MACULO-PAPULAR				1						1									1			3			6%
	SCALP PAIN	1																					1			2%
	SKIN HYPERPIGMENTATION													2									2			4%
	SKIN HYPOPIGMENTATION																1						1			2%
	SKIN INDURATION	1	3		1	1		2						11	3		1	1		2	3		18	11		58%
	SKIN ULCERATION													1						2			3			6%
VASCULAR	FLUSHING													5						1			6			12%
	HOT FLASHES							1			2			1			2			2			8			16%
	OTHER														1									1		2%

Supplemental Table 4. Expected and observed $CD8^+$ T cell response rates to MEPs included in LPV7 vaccines

Peptide	Amino acid sequence	HLA restric-tion	Mel44 trial (Arm A)		Current Trial		Difference (90% CI)
			# evaluable with relevant MHC I	$CD8^+$ IRR with IFA	# evaluable with relevant MHC I	CD8+ IRR	
Tyrosinase (240-251)	DAEKSDICTDEY	A1	12	42%	21	19%	23% (-5%, 50%)
MAGE-A10 (254-262)	GLYDGMEHL	A2	23	39%	20	0%	39% (22%, 56%)
gp100 (209-217)	IMDQVPFSV	A2	23	78%	20	0%	78% (64%, 92%)
Tyrosinase (369-377)	YMDGTMSQV	A2	23	4%	20	0%	4% (-3%, 11%)
gp100 (17-25_	ALLAVGATK	A3	15	53%	21	5%	49% (26%, 71%)
MAGE-A1 (96-104)	SLFRAVITK	A3	15	53%	21	5%	49% (26%, 71%)

Supplemental Table 5. Immune responses based on Institution

	Institution 1*					Institution 2*			
Study arm	N	LPV7	CD8/MEP	Tetanus		N	LPV7	CD8/MEP	Tetanus
A	4	2 (50%)	2 (50%)	4 (100%)		1	0 (0%)	0 (0%)	0 (0%)
B	5	2 (40%)	1 (20%)	3 (60%)		2	0 (0%)	0 (0%)	0 (0%)
C	3	0 (0%)	0 (0%)	0 (0%)		1	0 (0%)	0 (0%)	0 (0%)
D	5	1 (20%)	0 (0%)	1 (20%)		1	0 (0%)	0 (0%)	0 (0%)
E	7	5 (71%)	4 (57%)	5 (71%)		9	0 (0%)	0 (0%)	3 (33%)
F	3	1 (33%)	1 (33%)	1 (33%)		3	0 (0%)	0 (0%)	0 (0%)
G	4	4 (100%)	1 (25%)	3 (75%)		2	0 (0%)	0 (0%)	0 (0%)
IFA+ (A, E-G)	18	12 (67%)	8 (44%)	13 (72%)		15	0 (0%)	0 (0%)	3 (20%)
No IFA (B-D)	13	3 (23%)	1 (8%)	4 (31%)		4	0 (0%)	0 (0%)	0 (0%)
All	31	15 (48%)	9 (29%)	17 (55%)		19	0 (0%)	0 (0%)	3 (16%)

* Values shown are the number of participants with a T cell response to LPV7, CD8 minimal epitope, and Tet (and the proportion of those evaluable). Institution 1 = University of Virginia; Institution 2 = MD Anderson Cancer Center.

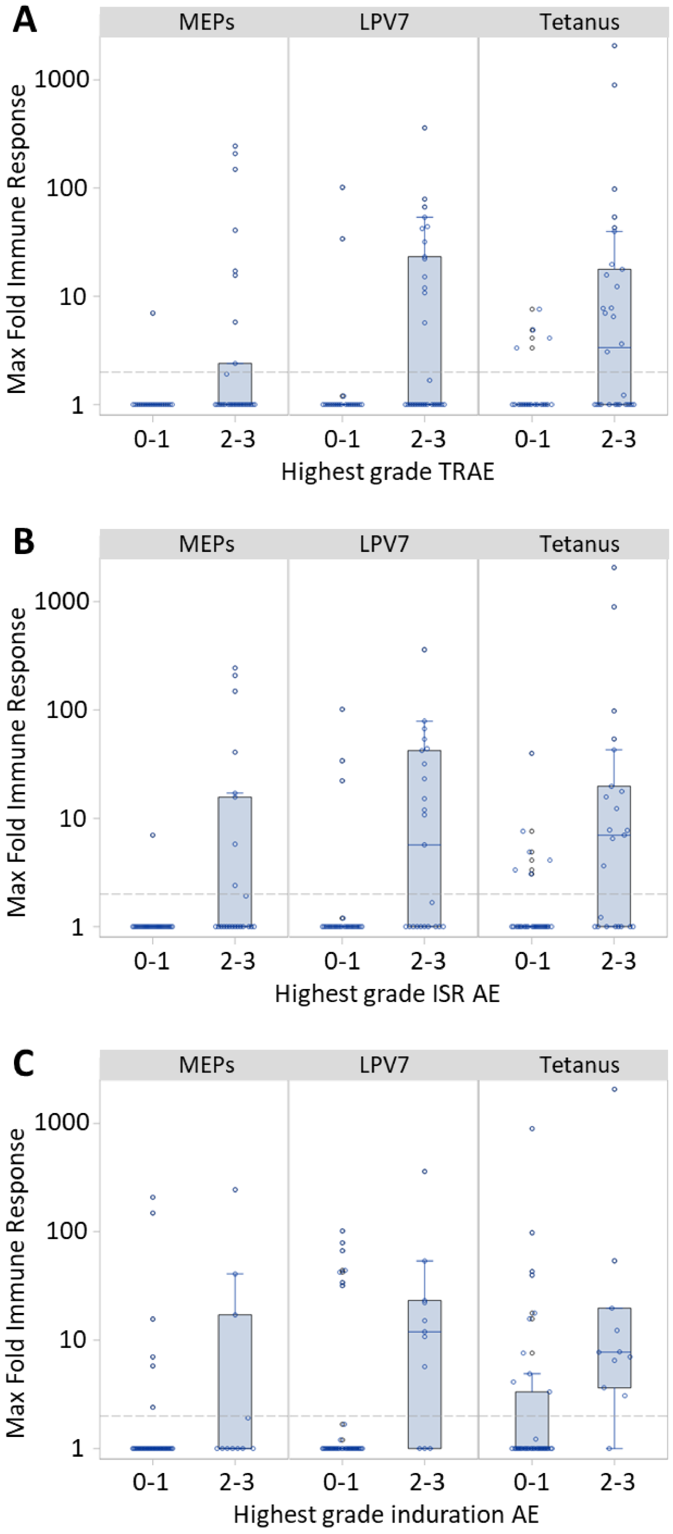

Supplemental Figure 1. Immune response to minimal epitope peptides (MEPs) for CD8 T cells, long peptides (LPV7) or tetanus helper peptide (Tetanus), as fold increase over background as a function of the maximum severity of TRAEs (A), ISR AEs (B), and vaccine site induration AEs (C), comparing participants with maximum grade 2-3 AEs vs those with grade 0-1 AEs.

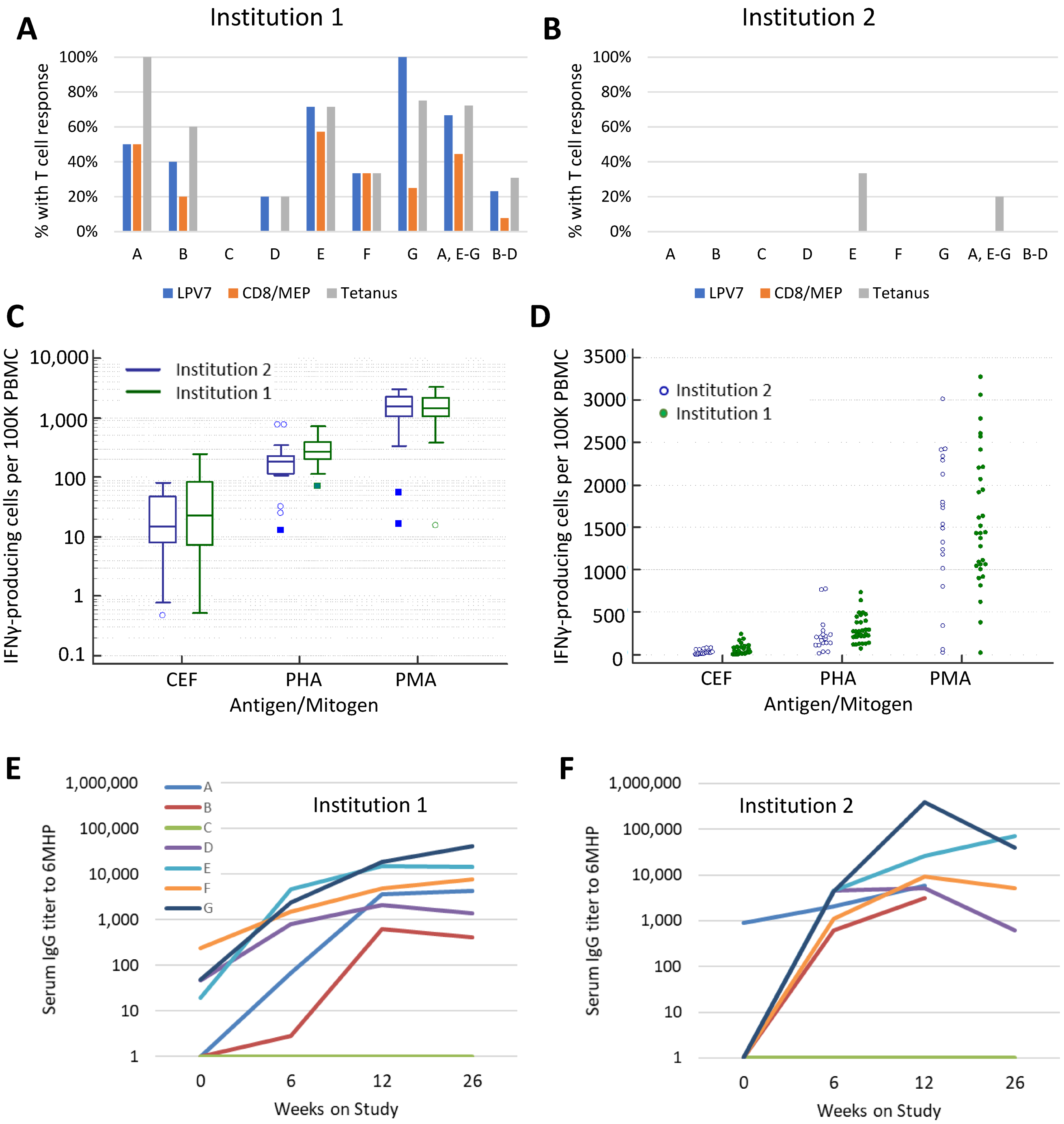

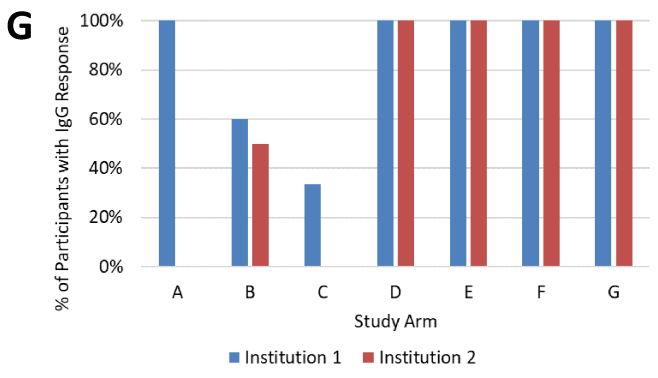

Supplemental Figure 2. Immune responses by Institution. T cell response rates to peptides in the vaccine at Institution 1 (A) and 2 (B) by study arm. Cellular immune responses (for each participant, mean for all samples) to positive control antigens (CEF), or mitogens shown on log scale (C) and linear scale (D). Serum IgG response to 6MHP at Institution 1 (E) and 2 (F). Proportion of participants with Ab response to 6MHP by site (G): including 4, 5, 3, 5, 7, 3, 4 at Institution 1 and 1, 2, 1, 1, 9, 3, 2 at Institution 2, respectively, for Arms A-G.

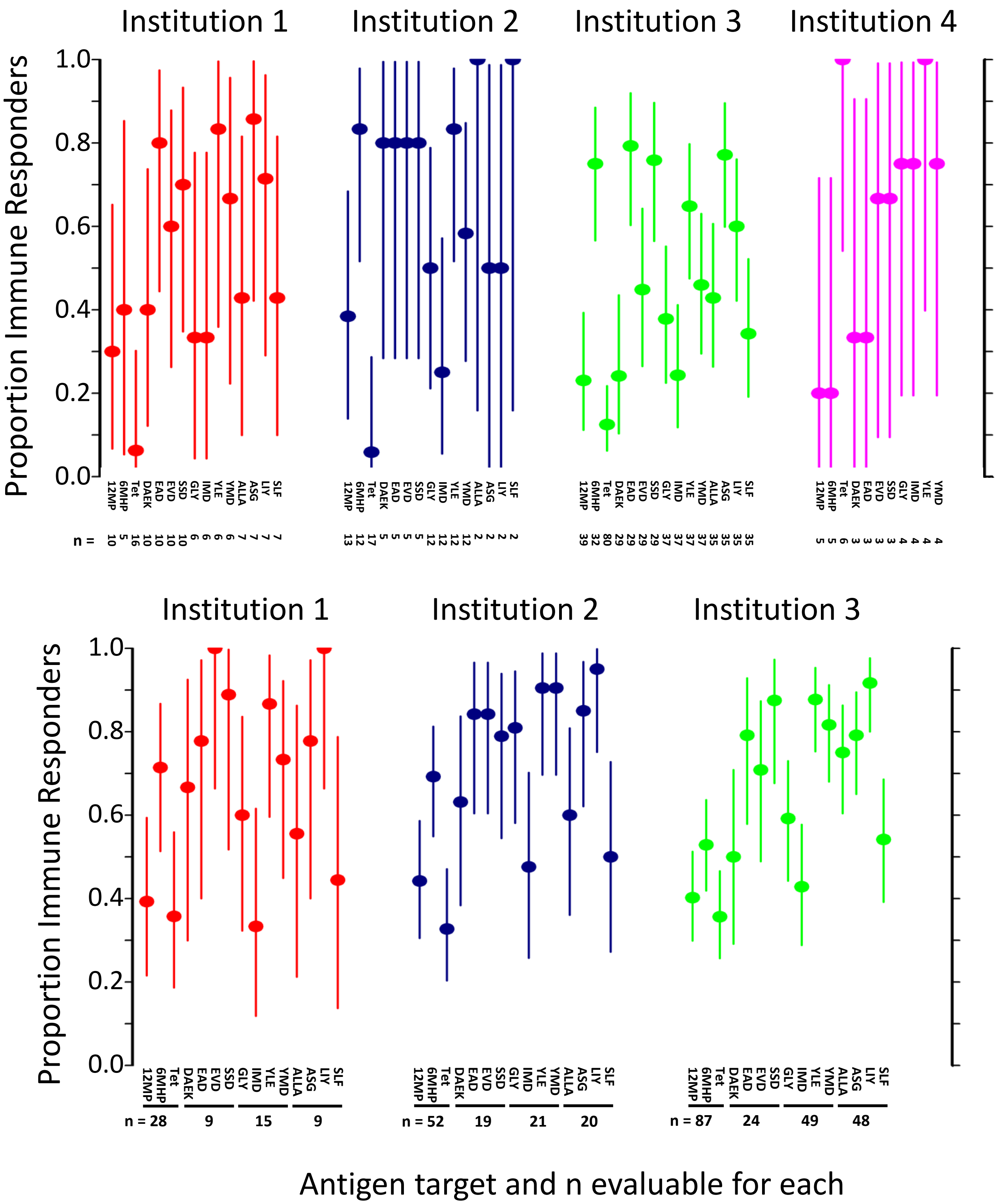

Supplemental Figure 3. Immune response rates as a function of participating institution. Immune response rates (circles) and 90% CI are shown for the mixture of 12 short melanoma peptides (12MP), mixture of 6 melanoma helper peptides (6MHP), tetanus peptide (Tet), and to individual short peptides DAEK to SLF) from two prior multicenter clinical trials, Mel43 (A), and Mel44 (B). Mel43 enrolled participants from 5 institutions, one of which was excluded because n=1; and Mel44 enrolled participants from 3 institutions. In both, institutions 2 and 3 are the institutions in the current manuscript.

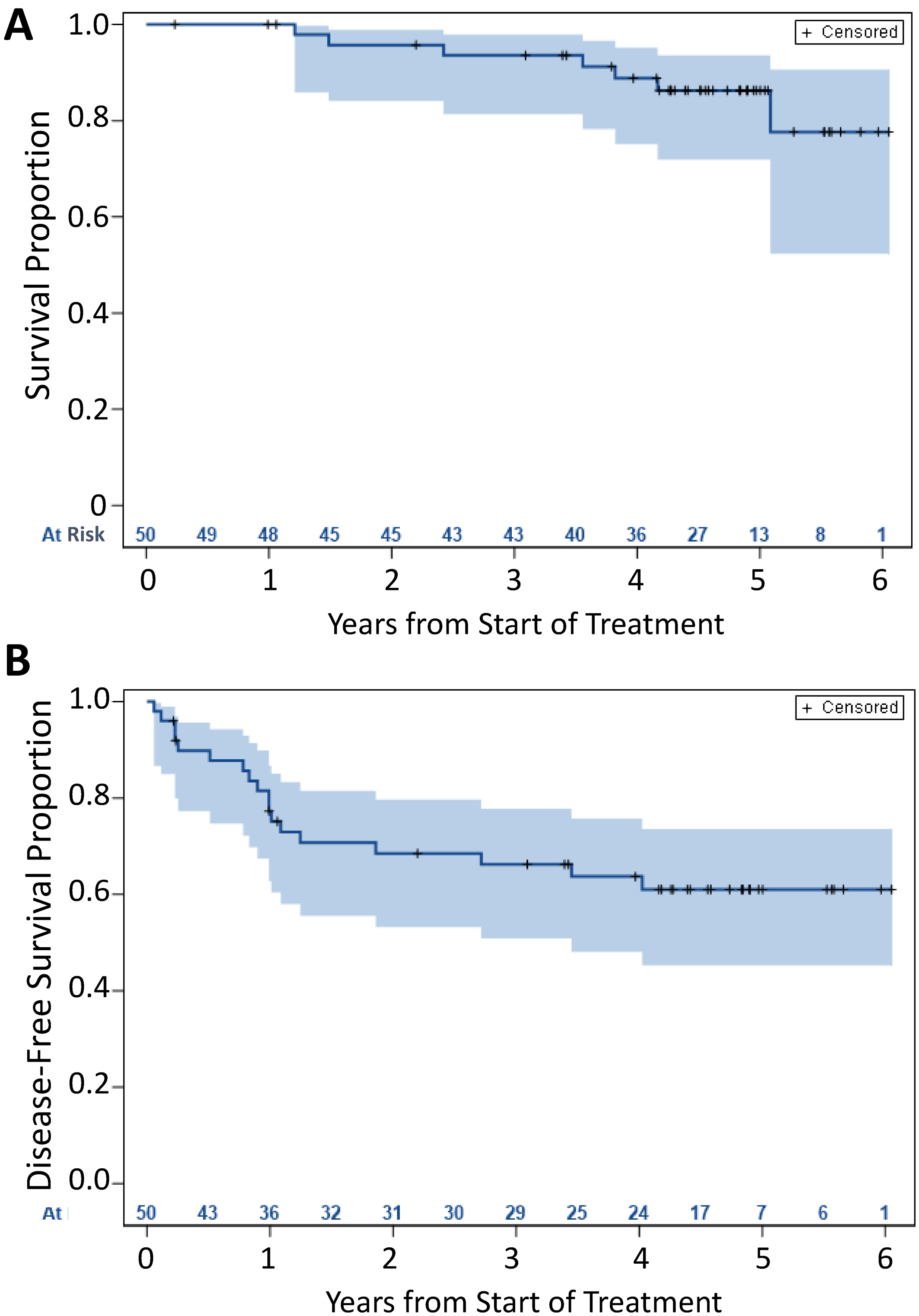

Supplemental Figure 4. Overall Survival (A) and Disease-Free Survival (B) for the 50 patients across all 7 study arms on the Mel60 trial. The shaded region represents 95% confidence limits.
