# Supplementary figures and images for: Phase I/II trial of a long peptide vaccine (LPV7) plus toll-like receptor (TLR) agonists with or without incomplete Freund’s adjuvant (IFA) for resected high-risk melanoma
Source: J Immunother Cancer. 2021 Aug 19;9(8):e003220. doi: 10.1136/jitc-2021-003220 (PMC8378357; doi:10.1136/jitc-2021-003220)

CONSORT FLOW DIAGRAM

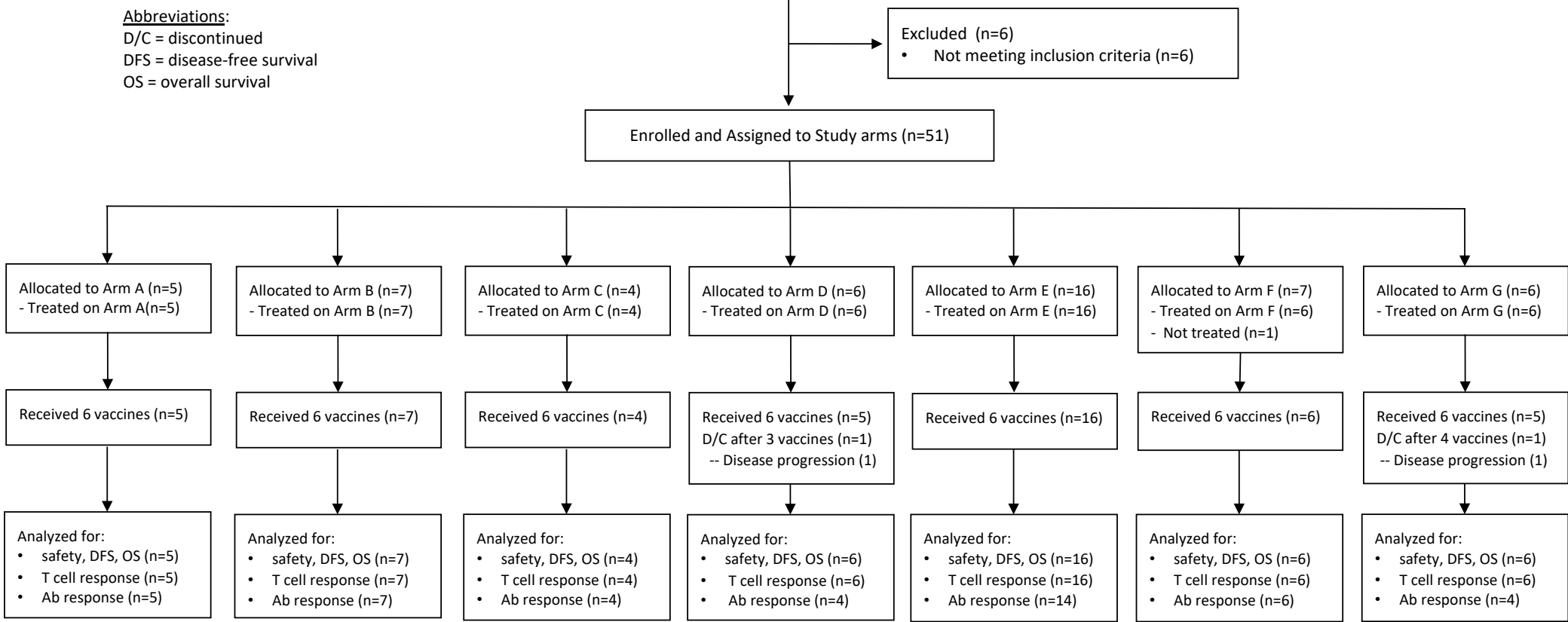

Supplement: Supplementary data [file jitc-2021-003220supp001.pdf]
